# OCRL1 Deficiency Affects the Intracellular Traffic of ApoER2 and Impairs Reelin-Induced Responses

**DOI:** 10.3390/biom14070799

**Published:** 2024-07-05

**Authors:** Luz M. Fuentealba, Héctor Pizarro, María-Paz Marzolo

**Affiliations:** Laboratorio de Tráfico Intracelular y Señalización, Facultad de Ciencias Biológicas, Pontificia Universidad Católica de Chile, Santiago 7810128, Chile; luz.fuentealba@gmail.com (L.M.F.); hrpizarro@uc.cl (H.P.)

**Keywords:** lowe syndrome, endosomal pathway, LRP8, Reelin, Golgi

## Abstract

Lowe Syndrome (LS) is a rare X-linked disorder characterized by renal dysfunction, cataracts, and several central nervous system (CNS) anomalies. The mechanisms underlying the neurological dysfunction in LS remain unclear, albeit they share some phenotypic characteristics similar to the deficiency or dysfunction of the Reelin signaling, a relevant pathway with roles in CNS development and neuronal functions. In this study, we investigated the role of OCRL1, an inositol polyphosphate 5-phosphatase encoded by the *OCRL* gene, mutated in LS, focusing on its impact on endosomal trafficking and receptor recycling in human neuronal cells. Specifically, we tested the effects of OCRL1 deficiency in the trafficking and signaling of ApoER2/LRP8, a receptor for the ligand Reelin. We found that loss of OCRL1 impairs ApoER2 intracellular trafficking, leading to reduced receptor expression and decreased levels at the plasma membrane. Additionally, human neurons deficient in OCRL1 showed impairments in ApoER2/Reelin-induced responses. Our findings highlight the critical role of OCRL1 in regulating ApoER2 endosomal recycling and its impact on the ApoER2/Reelin signaling pathway, providing insights into potential mechanisms underlying the neurological manifestations of LS.

## 1. Introduction

Lowe Syndrome (LS) is a rare genetic disorder linked to the X chromosome [1,2,3]. It manifests as a constellation of clinical features encompassing renal dysfunction, cataracts, and a spectrum of neurological abnormalities, including intellectual disabilities, seizures, and hypotonia [4,5,6,7]. While the renal manifestations of LS have been extensively studied, the underlying mechanisms contributing to the neurologic dysfunction remain elusive [8,9]. A limitation of the study of this condition has been the murine animal models for LS [8,10,11]; some of them do not show an apparent phenotype and do not recapitulate the complex neurological phenotype of the disease. Therefore, using relevant LS human cellular models such as neuronal cell lines and iPSCs-derived neurons is essential to find which processes are affected by this disease.

Mutations in LS occur in *OCRL* [12,13,14,15,16,17,18], the gene encoding for the inositol polyphosphate 5-phosphatase OCRL1 [17,19,20]. Many of the functions of this protein have been investigated in kidney-derived cells [18,21,22,23,24] and fibroblasts [18,25,26,27,28]. OCRL1 participates in several cellular functions, from maintaining the compartmentalization of membrane domains to determining cell polarity, controlling cytoskeletal rearrangements, and regulating signaling pathways and intracellular trafficking [17,18,21,22,23,24,25]. Defects in the function of OCRL1 prompt the accumulation of its substrates, predominantly phosphatidyl inositol 4,5 biphosphate (PI(4,5)P_2_) [21,29], disturbing actin polymerization and dynamics [21,30,31,32]. In addition, LS cells show spreading defects [27,33]. At the endolysosomal level, OCRL1 has several functions; it binds to clathrin and to the adaptor complex AP-2 to allow the uncoating of vesicles [6], participates in maintaining autophagic flux [23] and regulates lysosome positioning to the cell periphery [34]. The dysregulation of OCRL1 halts various receptors, such as Megalin/LRP2, Transferrin Receptor (TfR), Epidermal Growth Factor Receptor (EGFR), and CI-Mannose-6-Phosphate Receptor (CI-MPR) in enlarged sorting endosomes characterized by the presence of the tethering protein Early Endosome Antigen 1 (EEA1); these receptors are accumulated and do not proceed to their destination to the plasma membrane in the recycling pathway, to late endosomes for degradation, or the Golgi complex in the retrograde pathway [21,27].

ApoER2/LRP8 belongs to the Low-Density Lipoprotein Receptor-Related Protein (LRP) family as Megalin [35]. ApoER2 is predominantly expressed in the brain [36,37] but is also found in the peripheral nervous system [38] and endocrine organs [39]. At a cellular level, ApoER2 is constitutively internalized [40]. From here, ApoER2 is recognized by endosomal sorting protein 17 (SNX17), which leads the recycling of ApoER2 either by Rab11 recycling endosomes or directly to the plasma membrane, escaping lysosomal degradation [41].

The primary role of ApoER2 in the central nervous system (CNS) is as a receptor for Reelin, a secreted glycoprotein mainly involved in brain development, learning, and memory [42]. Reelin binds to ApoER2, inducing its clustering and the recruitment and phosphorylation of the adaptor protein Dab1 [42,43]. Then, PI-3K is activated, followed by the activation of AKT [44,45] and Rho GTPases as Rac1 and Cdc42 [38,46], required for the orientation and migration of multipolar cortical neurons, Golgi deployment [47,48], and growth and branching of dendrites [45,49]. ApoER2/Reelin also participates in the regulation of gene expression through the activation (phosphorylation) of ERK and CREB [50,51], with a relevant role of the ApoER2 intracellular domain [52,53], which acts on specific enhancers forming a complex with pCREB [50].

The absence or reduction of the ApoER2/Reelin signaling pathway has been associated with cognitive dysfunction [54], neuropsychiatric disorders [55,56], and neurodegenerative diseases [57]. Mutations in *RELN*, as well as the reduction of Reelin expression, are associated with intellectual disability, learning, and memory deficits [58,59,60], and neurodegeneration [61,62,63]. Moreover, homozygous null mutations of *RELN* lead to lissencephaly, a disease characterized by the absence of regular convolutions in the cerebral cortex and by cerebellar hypoplasia, neonatal hypotonia, and mental retardation [64]. Interestingly, the CNS abnormalities in LS include hypotonia, seizures, and cognitive problems, such as intellectual disability, maladaptive behaviors, and developmental delay [4,7,65]. Therefore, there are similarities in many of the manifestations associated with a decrease in Reelin signaling and the phenotypes described for LS patients.

Remarkably, the inhibition in ApoER2 recycling and surface expression induced by SNX17 knock-down significantly impairs Reelin function [41]. Accordingly, the link between ApoER2 intracellular trafficking and its ability to respond as a Reelin signaling receptor is essential for the pathway functioning. Thus, as with Megalin and other receptors, OCRL1 activity as a modulator of endosomal recycling to the plasma membrane could positively regulate the ApoER2 pool capable of responding to Reelin.

In this work, we proposed that an impairment in Reelin-induced responses due to a defect in ApoER2 trafficking would partially explain the neuronal phenotype of LS patients. We found that the loss of OCRL1 affected ApoER2 intracellular trafficking, leading to reduced protein total and surface levels, directly impacting Reelin-induced responses measured in human cortical neurons.

## 2. Materials and Methods

### 2.1. Cell Culture

The human neuroglioma cell line H4 (American Type Culture Collection (Manassas, VA, USA)) was provided by Dr. Patricia Burgos (USS, Santiago, Chile). Cells were cultured in high-glucose DMEM (Gibco^TM^, Waltham, MA, USA, 12100046) supplemented with 10% fetal bovine serum (FBS, Biological Industries, Beit HaEmek, Israel, 04-127-1A), 1X GlutaMAX (Gibco^TM^, 35050079), and antibiotics (Gibco^TM^, 15140163). The cells were maintained until they reached 80–90% confluency, after which they were seeded onto 12 or 24-mm diameter coverslips for imaging experiments or 35-mm diameter wells for biochemical experiments.

The human isogenic iPSC line containing an inducible Neurogenin 2 transgene for the conversion to glutamatergic cortical neurons (i3 neurons) was generated from a wild-type genetic background human iPSC line WTC11 [66] and donated by Dr. Michael Ward (NIH, Bethesda, MD, United States) [67]. Cells were cultured and differentiated to i3 neurons using a previously described protocol [67,68]. Briefly, the plates for culturing iPSCs were coated with Matrigel (Corning, New York, USA, 354277) in DMEM/F12 (Gibco^TM^, 11320033) for 30 min at 37 °C. iPSCs were cultured in Essential 8 medium (Gibco^TM^, A1517001) supplemented with antibiotics (Gibco^TM^, 15140163) and 10 µM Y-27632 dihydrochloride (ROCK inhibitor, 1254, Tocris Bioscience, Bristol, UK). The ROCK inhibitor was included only when the confluency of the iPSCs was less than 40%. When the iPSCs reached 70–80% confluency, they were passaged using StemPro Accutase (Gibco^TM^, A1110501) and seeded onto Matrigel-coated dishes with Induction Medium (DMEM/F12 with HEPES (Gibco, 11330032), N2 supplement (Gibco, 17502048), non-essential amino acids (NEAA, Gibco, 11140050), GlutaMAX (Gibco^TM^, 35050079)), and supplemented with ROCK inhibitor and 2 µg/mL Doxycycline (DOX, Sigma, Burlington, MA, USA D9891). The pre-differentiated cells were maintained for 3 days under these conditions.

Pre-differentiated cells were dissociated using StemPro Accutase, counted, and seeded onto PLO-coated dishes in the presence of Cortical Medium. Cell culture plates were incubated overnight with poly-L-ornithine (PLO, Sigma, Burlington, MA, USA P3655) and washed three times with water to prepare PLO-coated dishes. Cortical Medium consisted of BrainPhys Neuronal medium (STEMCELL Technologies, Vancouver, BC, Canada, 05790) supplemented with B27 (Gibco^TM^, Waltham, MA, USA, 17504044), 10 ng/mL BDNF (PeproTech, Cranbury, NJ, USA, 450-02), 10 ng/mL NT-3 (PeproTech, Cranbury, NJ, USA 450-03), and 1 µg/mL Laminin (Gibco^TM^, Waltham, MA, USA, 23017015). Throughout the culture period, half of the media was removed every 3–4 days or once a week, and fresh media containing DOX was added to sustain the i3 cortical neurons. These cells were maintained in culture until they were ready for experimentation.

### 2.2. CRISPR/Cas9 OCRL Knock-Out

CRISPR/Cas9 was employed to generate *OCRL* KO H4 and iPSCs. First, two specific guide RNAs (sgRNA1: 5′-GCTGTTCCTTCTCATGCAAC and sgRNA2: 5′-GCTGCAAAATTCGGGTTCAG GGG) targeting the *OCRL* gene were designed using CHOPCHOP (https://chopchop.cbu.uib.no/ (accessed on 21 February 2021)) [69,70] and Broad Institute platforms (https://portals.broadinstitute.org/gpp/public/analysis-tools/sgrna-design (accessed on 21 February 2021)) [71]. The sgRNAs were cloned into the pX-458 vector at the Bbs I restriction site, following the protocol described by Ran et al. (2013) [72]. Lipofectamine Stem (Invitrogen, Waltham, MA, USA, STEM00001) was used to transfect the sgRNAs into iPSCs, and Lipofectamine 2000 (Invitrogen, Waltham, MA, USA, 11668019) was used for H4 cells. 24 h after transfection, GFP-positive cells were sorted using FACSAria II (BD Biosciences, San Jose, CA, USA) and plated into 96-well plates as two cells per well, collecting at least two 96-well plates per sgRNA. Few wells without cells or with two cells were discarded. Cells were cultured in a complete medium for 1–2 weeks, allowing them to form colonies. Subsequently, individual colonies were transferred to 6-well dishes and cultured until they reached confluency. Clones lacking the protein OCRL1 were identified by western blot. The selected clones were expanded and stored for further experimentation.

For iPSC *OCRL* KO cells, promising clones were subjected to PCR amplification of the region containing the target sequence of the sgRNA, which produced a 585 bp fragment. 10% of the volume from PCR was analyzed by electrophoresis on a 1% agarose gel, and the positive fragments were subsequently sequenced in ABI PRISM 3130 XL (Applied Biosystems, Foster City, CA, USA). A clone with a one-base addition resulting in an early stop codon was selected.

### 2.3. Transfection of HA-ApoER2

The plasmids pcDNA3 encoding for HA-ApoER2 and RAP [40] were transfected in H4 cells using Lipofectamine 2000 (Invitrogen, Waltham, MA, USA), according to the table provided by the supplier. After waiting the corresponding time for complex formation (30 min at RT), the Liposomes/DNA were added dropwise directly to the cell medium. The medium was changed 4 h later.

### 2.4. Western Blot

H4 and human i3 neurons were lysed in PBS containing 1% Triton X-100 and phosphatase/protease inhibitors (1 mM glycerophosphate, 1 mM sodium orthovanadate, 5 mM sodium fluoride, 2 mM PMSF, 1 mM pepstatin, 2 μM antipain, 1 μM leupeptin, and 0.3 μM aprotinin). Extracts were centrifuged at 14000 rpm for 10 min and protein concentration was determined in the supernatant. Samples were boiled in Laemmli sample buffer (62.5 mM Tris-HCl, pH 6.8; 2% *w*/*v* SDS, 10% *v*/*v* glycerol, and 5% ß-mercaptoethanol) and then separated by SDS-PAGE under reducing conditions. Gels were transferred to PVDF membranes (Thermo Fisher, Waltham, MA, USA). The membranes were blocked in TBS containing 0.1% Tween-20 and 5% non-fat milk and subjected to incubation overnight with primary antibodies and for 2 h with secondary antibodies. Blots were developed with ECL, signal was acquired with a UVITEC system and analyzed with Fiji 2.15.1 [73]. A Table with a detailed list of the antibodies used in western blot is in Supplemental Table S1.

### 2.5. Immunofluorescence

H4 cells, human iPSCs, and human i3 neurons were washed with PBS and fixed with 4% PFA and 4% Sucrose in PBS (PFA/sucrose) for 20 min at room temperature. Cells were washed three times with 1X PBS and then permeabilized for 10 min with 0.2% *v*/*v* Triton X-100 at room temperature. Then, cells were blocked for 1 h with PBS containing 3% BSA. The antibodies were diluted in the same blocking reagent and incubated in a wet chamber at 4 °C overnight. Next, coverslips were washed three times with 1X PBS before incubation with the corresponding Alexa Fluor secondary antibody. After washing with 1X PBS, coverslips were mounted with Fluoromount-G (495802, Invitrogen, Waltham, MA, USA). A Table with a detailed list of the antibodies used in immunofluorescence is in Supplemental Table S2.

### 2.6. Shell Analysis

We followed the steps described by Williamson et al. (2022) [74] to perform shell analysis. Briefly, H4 cells were labeled for LAMP1 and imaged using confocal microscopy. Images were processed in Fiji [73] to generate maximum-intensity projections and draw lines connecting the nucleus and cell edge. The average line length was calculated and then divided by 8, which is the number of shells used in our analysis. This determined the gap width between consecutive shells, and the analysis was adjusted using this parameter. The data from the Results window were stored in a spreadsheet. Further analysis was performed with Matlab R2022b. The lysosomal signal within each shell was determined by subtracting the lysosomal area of each consecutive ROI from the ROI before it. The fraction of total lysosomes within each shell was calculated by dividing the area per shell by the total lysosomal area.

### 2.7. Spreading Assay

The spreading assay was performed as reported [18] with a few modifications. Briefly, iPSCs were lifted with 20 mM EDTA for 15 min, centrifuged at 200× *g* for 5 min, resuspended in supplemented E8 medium, and set in a rotator for 45 min. Then, cells were seeded on Matrigel-coated coverslips in a supplemented E8 medium and allowed to attach and spread for 30 min at 37 °C. Coverslips containing attached cells were gently washed, fixed, and stained with Alexa Fluor 647-coupled phalloidin (A22287 Invitrogen, Waltham, MA, USA) and DAPI. Images were captured by epifluorescence microscopy; cell boundaries were traced with the Freehand selection tool from Fiji and the area was determined for each cell [73].

### 2.8. Neurite Length Measurements

Human i3 neurons were transfected with pEGFP after three days of differentiation, using Lipofectamine 3000 (L3000001, Invitrogen) according to manufacturer instructions. 48 h later, neurons were fixed with PFA/sucrose for 20 min and subjected to immunofluorescence staining to identify dendrites using a chicken anti-MAP2 antibody. Widefield microscopy, specifically a Nikon Ti microscope with a 20× objective, was used to capture individual cell images of MAP2 and GFP-positive cells. For each experimental condition, a total of 10 random images were acquired. The length of neuronal processes was obtained using the Neuron J plugin https://imagej.net/plugins/neuronj, which tracks the process manually.

### 2.9. ApoER2 Surface Levels

H4 cells were transfected with plasmids encoding for HA-ApoER2 and RAP, and 24 h later, the cells were washed three times with cold 1X PBS on ice. Cells were incubated for 1 h at 4 °C with anti-HA coupled to Alexa Fluor 488 in cold DMEM/HEPES/BSA (DMEM high glucose, 20 mM HEPES, 2% BSA). Cells were washed with 1X PBS, fixed with PFA/sucrose, washed with 1X PBS, and then permeabilized with 0.2% *v*/*v* Triton X-100. Fixed cells were blocked with 3% BSA in PBS 1X and incubated with chicken Anti-HA primary antibody at 4 °C overnight, and subsequently incubated with anti-chicken coupled with Alexa Fluor 647, washed, and mounted.

### 2.10. Endocytosis/Internalization

H4 cells were plated in 12 mm coverslips and transfected with plasmids encoding HA-ApoER2 and RAP. Cells were placed on ice, washed with cold DMEM/HEPES/BSA, and incubated with chicken anti-HA antibody at 4 °C for 45 min. Cells were washed with cold DMEM/HEPES/BSA, and then 400 µL of warm DMEM/HEPES/BSA was added. Plates were transferred to 37 °C, at different times, finishing by quickly plating the coverslips on ice and washing them three times with cold PBS. The coverslips were washed three times with an ice-cold acidic solution (0.1 M NaCl, 0.1 M Glycine, pH 3.0) to detach the non-internalized antibody and three times with cold PBS. After fixation with PFA/sucrose, cells were washed with PBS, permeabilized with PBS/Triton, blocked with PBS/BSA, and incubated with primary antibodies overnight. The coverslips were washed with PBS and incubated with secondary antibodies. Finally, the cells were mounted, including DAPI in the mounting media.

### 2.11. ApoER2 Recycling

H4 cells expressing HA-ApoER2 were incubated on ice with anti-HA coupled with Alexa fluor 488 (anti-HA-488) at 4 °C for 45 min. Not-bound antibody was washed, and cells were allowed to internalize the surface proteins for 30 min at 37 °C, then transferred to ice. Non-internalized HA-488 was quenched with anti-Alexa fluor 488 (anti-488) at 4 °C for 45 min, and a coverslip was fixed to measure internalized ApoER2. After washing the non-bound quencher antibody, the coverslips were placed at 37 °C for an additional 30 min, allowing the recycling of internalized ApoER2. The ApoER2 bound to anti-HA-488 that returned to the cell surface was quenched with anti-488 for 45 min at 4 °C. The coverslips were washed, fixed, and mounted. Overnight dried preparations were imaged in a widefield microscope with a 60× objective. The mean intensity was measured for each cell with Fiji [73], then the percentage of recycled ApoER2 was obtained as follows:

% recycled protein = (ai − ar)/ai

where ai is the average of internalized intensity and ar was the average of recycled intensity. The average intensity was obtained from at least 50 cells measured.

### 2.12. ApoER2 Half Life

ApoER2 half-life was determined in differentiated i3 neurons cultured on PLO-coated 6-well plates. After 21 days of differentiation, the neurons were treated with 25 μM cycloheximide for specified durations in complete cortical medium (CM) for up to 24 h. At the end of the incubation period, cells were washed once with PBS and lysed using a lysis buffer containing PBS with 1% Triton X-100, 1 mM glycerophosphate, 1 mM sodium orthovanadate, 5 mM sodium fluoride, and a mixture of protease inhibitors. The lysed samples were then subjected to SDS-PAGE and visualized by western blotting to determine the ApoER2 levels.

### 2.13. Preparation of Recombinant Reelin

Media containing mouse Reelin was obtained from HEK293 cells expressing the full-length protein. The cells were cultured as previously described to produce a Reelin-conditioned medium, RCM [75]. The mock-conditioned medium (MCM) was prepared from HEK293, expressing the empty vector using the same protocol. Cells were cultured in high-glucose DMEM supplemented with 10% FBS, penicillin, streptomycin, and 0.5 mg/mL G418 at 37 °C until they reached 80% confluency. After two washes with PBS, the cells were cultured in high-glucose DMEM without serum at 37 °C for 24 h. The cell medium was collected and centrifuged at 2000 rpm for 10 min, and the resulting supernatant was stored at 4 °C. This procedure was repeated two more times. The collected medium was concentrated using Amicon ultra-15 centrifugal filter units with a 100 kDa filter membrane. Reelin concentration was determined after running a sample of RCM and different amounts of BSA in SDS-PAGE, detecting protein with Coomassie blue, and quantifying the bands using Fiji 2.15.1 [73].

### 2.14. ApoER2/Reelin Signaling

As part of Reelin responses, two branches of the pathway were studied, the one that included AKT phosphorylation and the other the activation of ERK. Human i3 neurons with 21 days of differentiation were depleted of supplements for 1 h with HBSS. 10 nM of Reelin conditioned medium or mock was added to the cultures and incubated for 10, 20, and 40 min at 37 °C. Protein samples from human i3 neurons were subjected to SDS-PAGE and western blot to detect pAKT, pERK, and the corresponding total proteins. Data was plotted and analyzed with GraphPad.

### 2.15. Reelin-Induced Golgi Deployment

Human i3 neurons were differentiated for 12 days. Cells were starved for 2 h with HBSS followed by treatment with 10 nM Reelin or mock for 30 min. The i3 neurons were then fixed and stained with anti-GM130, anti-MAP2, and DAPI. Confocal images were analyzed using Fiji [73]. For measuring Reelin-induced Golgi deployment, a straight line was drawn from the outermost edge of the nucleus, extending to the Golgi membrane complex, using the straight-line tool. The length of the line was measured for each condition and plotted accordingly.

### 2.16. Microscopy and Image Analysis

Unless otherwise stated, images were captured with a confocal microscope Nikon Timelapse equipped with a Plan Apochromat 63× objective provided by UMA-PUC. First, overnight dried coverslips from controls were scanned and lasers were set at a minimum to detect a good signal vs. noise ratio. Settled parameters were used to capture images of random fields from all conditions. A theoretical Point Spread Function was calculated based on the Richards and Wolf 3D Optical Model [76] for deconvolution of images. Images were deconvolved using the Deconvolution lab with Tikhonow-Miller’s algorithm [77].

The colocalization analysis was performed by drawing a ROI per cell and calculating Mander’s colocalization coefficient using the JaCoP [78] colocalization plugin in Fiji [73]. ROIs were generated with a macro that opened the image and the ROI Manager tool; then, the edges of a cell were traced using the freehand selection tool and added to the ROI Manager. All ROIs for each image were saved, and cropped cells were used for further analysis. The acquired data were plotted using Prism 9 software. For widefield microscopy, images were obtained using a Nikon Eclipse Ti2 inverted microscope (Nikon, Tokyo, Japan) and a Leica DM2000 microscope (Leica, Wetzlar, Germany) equipped with an Axiocam 202 mono Zeiss microscope camera (Zeiss, Oberkochen, Germany).

### 2.17. Statistical Analysis

The immunofluorescence images and immunoblots were analyzed using Fiji [73]. Each data set was normalized to the wild-type (control) condition for comparison and analyzed with GraphPad Prism 9. The results in bar plots are presented as mean ± SEM (standard error of the mean). The scattered dot plots show a line at mean and lines at ± SEM. Mann–Whitney’s test and *t*-test were employed to determine the statistical significance between the two conditions. As indicated in the legends, ANOVA was used for multiple comparisons with specific tests.

## 3. Results

### 3.1. Characterization of OCRL Knock-Out Neuronal Cells

**Figure 1:**
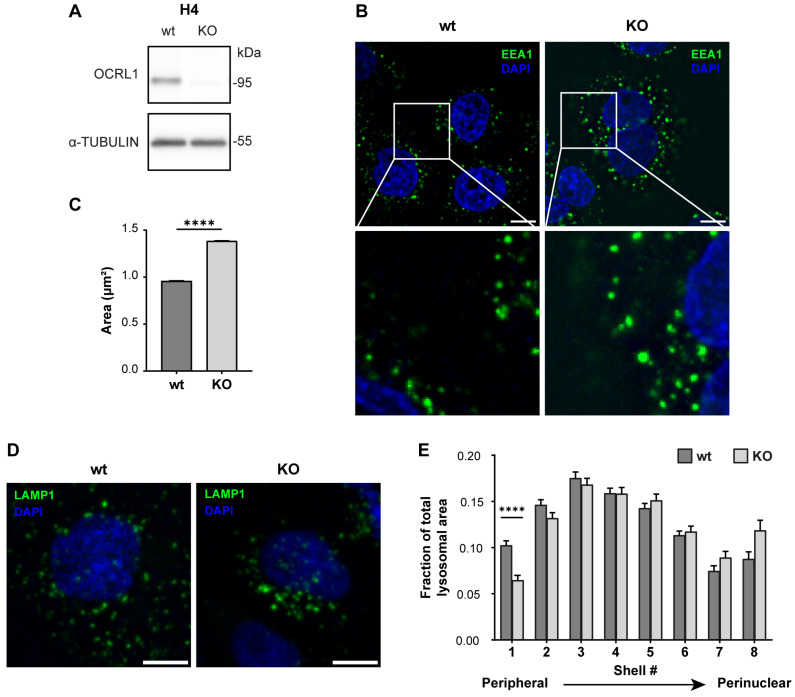
H4 *OCRL* KO cells recapitulate LS cellular phenotype. H4 cells were transfected with a plasmid containing Cas9 and specific sgRNA to target OCRL, selected, and propagated to perform further experiments. (**A**) Whole-cell lysate protein samples from wild-type or *OCRL* KO cells were separated by SDS-PAGE followed by western blot to detect OCRL1 and tubulin. (**B**,**D**) 48 h after seeding, the cells were fixed and stained for (**B**) EEA1 or (**D**) LAMP1. Scale bar = 10 µm. (**C**) EEA1 signal from (**B**) was analyzed with Fiji to measure the relative size of EEA1-positive endosomes. Data from 3 experiments was analyzed with GraphPad. Error bars indicate SEM. Mann–Whitney test, **** *p* < 0.0001. (**E**) LAMP1 signal from (**D**) was used to perform shell analysis with Fiji. At least 50 cells were analyzed from three experiments. Error bars indicate SEM. Significant differences were estimated using ANOVA, and multiple comparisons were calculated using Dunnett’s test. **** *p* < 0.0001. Original images can be found in Supplementary Materials.

As OCRL1 is the protein mutated in LS, and to generate an LS model in an ApoER2-expressing cell, we made a CRISPR KO of *OCRL* in the human neuroglioma cell line H4. First, we confirmed the effectiveness of our KO by measuring the OCRL1 levels by western blot. As shown in Figure 1A, we did not detect OCRL1. Other groups have reported several endosomal alterations as part of the LS phenotype [18,26]. We found an increase in the relative size of EEA1-positive endosomes, consistent with an increment of EEA1 at the early endosomal level (Figure 1B,C).

In addition, we performed a shell analysis to assess the intracellular distribution of lysosomes (LAMP1-positive structures) from the plasma membrane to the nucleus [74]. Lysosomes were dispersed along the cell with an evident peripheral localization in H4 wild-type cells. Meanwhile, *OCRL* KO H4 cells showed decreased peripheral LAMP1-positive structures (Figure 1D,E), suggesting a lysosomal trafficking disruption [34].

**Figure 2:**
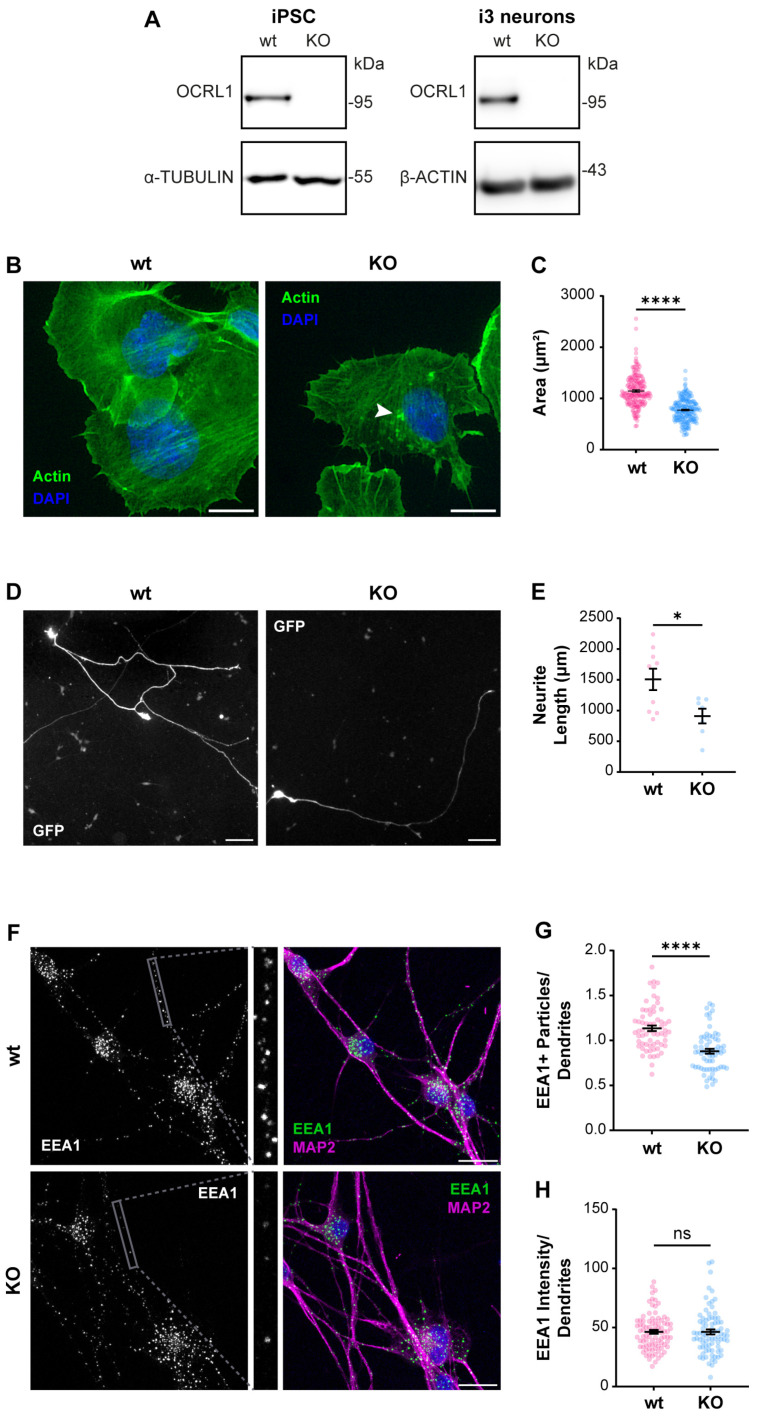
Characterization of *OCRL* KO iPSC and i3 neurons. iPSCs were transfected with a plasmid containing Cas9 and sgRNA directed to *OCRL*, selected, and then differentiated for 21 days to obtain i3 neurons. (**A**) Protein extracts from wild-type and *OCRL* KO cells were subjected to SDS-PAGE and western blot to detect OCRL1 and tubulin or β-actin. (**B**) Wild-type and *OCRL* KO iPSC at 70% confluency were detached and seeded. Following an incubation of 30 min, the cells were fixed and stained with phalloidin and DAPI. Images were captured using a wild-field microscope. Arrowheads indicate actin accumulations. Scale bar = 20 µm. (**C**) The total area of each cell, determined by phalloidin labeling, was quantified. At least 50 cells from three independent experiments were analyzed. Statistical significance was calculated using a *t*-test (**** *p* < 0.0001). (**D**) Wild-type and *OCRL* KO iPSC were differentiated for three days, transfected to express GFP, and fixed 48 h later. Images were captured using a wild-field microscope. Scale bar = 100 µm. (**E**) Neurite length was measured from the GFP signal using Neuron J. Data from seven cells per experimental condition were analyzed, and statistical significance was calculated using a *t*-test (* *p* < 0.05). (**F**) Wild-type and *OCRL* KO i3 neurons were differentiated for 14 days, fixed, and stained for EEA1 (green), MAP2 (magenta) and DAPI (blue). Scale bar = 20 µm. (**G**) EEA1-positive particles were measured in dendrites (MAP2-positive structures, grey box). Data from 50 ROIs from three independent experiments were analyzed and plotted. Statistical significance was calculated using a *t*-test. **** *p* < 0.0001. (**H**) EEA1 signal intensity was measured in dendrites (MAP2-positive structures). Data from 50 ROIs from three independent experiments were analyzed and plotted. Statistical significance was calculated using a *t*-test. ns: *p* > 0.05. Original images can be found in Supplementary Materials.

As we recently reported, an appropriate physiologic model to study ApoER2 function is the human cortical neuron i3 [75]. We generated a CRISPR KO of *OCRL* in human iPSC that can be differentiated to cortical neurons [67,75], not detecting OCRL1 positive signal in any *OCRL* KO cells, either iPSCs or cortical neurons (Figure 2A). In KO iPSCs, we detected a reduction in the spreading area (Figure 2B,C), probably associated with an alteration in Rac1/RhoA activation balance [27,28]. Furthermore, upon five days of differentiation to cortical neurons, the OCRL1 deficient i3 neurons exhibited a significant decrease in neurite length (Figure 2D,E). Likewise, a notable reduction in neurite length was observed in rat hippocampal neurons in primary culture, treated for 24 or 48 h with the OCRL1 inhibitor YU142670 (Figure S1) [79]. Strikingly, in *OCRL* KO i3 neurons, there was a reduction in the number of EEA1-positive particles in a MAP2-positive area (Figure 2F,G). A reduction in early endosomes was previously reported in a zebrafish model [80]. Our results confirm alterations consistent with a phenotype of LS in our *OCRL* KO models.

### 3.2. OCRL KO Cells Show Significant Alterations in ApoER2 Protein Levels and Trafficking

The endosomal traffic of several receptors is affected in LS [21,22,23]. In human neurons, there is no information about alterations of relevant receptors and their associated signaling pathways that could underlie the neurological phenotype of LS patients. Thus, we evaluated protein levels and localization of ApoER2 as a crucial receptor of Reelin involved in neuronal development and function [81].

**Figure 3:**
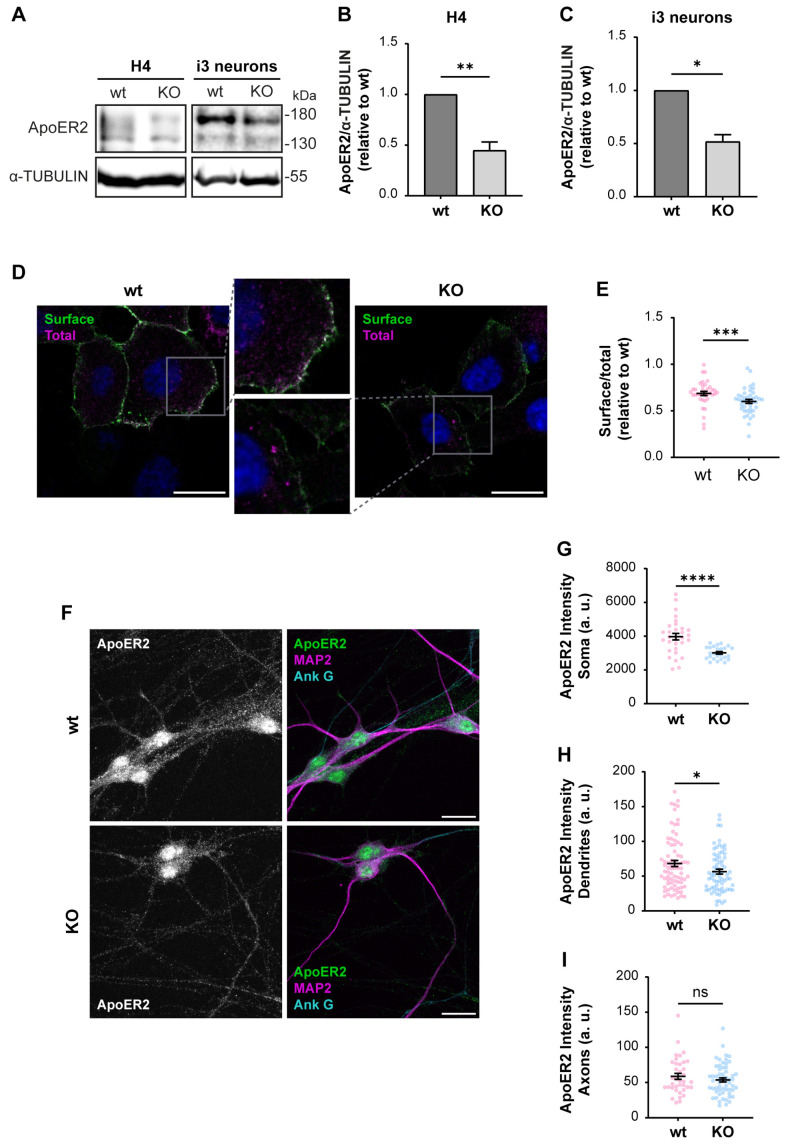
Decreased ApoER2 protein levels in *OCRL* KO cells. Wild-type and *OCRL* KO H4 and i3 neurons were grown for indicated days; then cell lysates were prepared. (**A**) Proteins were separated by SDS-PAGE, followed by western blot, and visualized by the UVITEC photo-documentation system to detect ApoER2 and α-tubulin. (**B**) Signal intensities from ApoER2 and α-tubulin in H4 cells (**A**, left) and (**C**) i3 neurons differentiated for 21 days (**A**, right) were measured by Fiji as the area below the curve for each band. The ratio of ApoER2 bands relative to α-tubulin was normalized to wild-type, plotted, and analyzed with GraphPad. Significant differences were calculated with a *t*-test for three independent experiments * *p* < 0.05, ** *p* < 0.01. (**D**) H4 cells were transfected with plasmids for HA-ApoER2 and RAP expression. 24 h later, the surface receptor (green) was stained, incubating the cells with Alexa fluor 488-coupled anti-HA for 45 min at 4 °C, followed by fixation, permeabilization, and staining of total ApoER2 (magenta) with chicken anti-HA and corresponding secondary antibody and DAPI (blue). Confocal images were deconvolved and analyzed with Fiji. Scale bar = 20 µm. (**E**) Ratios of surface vs. total signals were calculated from at least 20 cells per experiment. Data were normalized to wild-type ratios and plotted with GraphPad. Significant differences were calculated with a Mann–Whitney test from three different experiments. *** *p* < 0.001. (**F**) Wild-type and *OCRL* KO i3 neurons were differentiated for 14 days, fixed, and stained for ApoER2 (green), ANK G (cyan), and MAP2 (magenta). Scale bar = 20 µm (**G**–**I**) ApoER2 intensities were measured in (**G**) Soma, (**H**) Dendrites (MAP2-positive structures) and (**I**) Axons (MAP2-negative structures). Data from 25 neurons from two independent experiments were analyzed and plotted. Statistical significance *t*-test with Welch’s correction. ns: *p* > 0.05, * *p* < 0.05, **** *p* < 0.0001. Original images can be found in Supplementary Materials.

First, we determined if ApoER2 protein levels were altered in *OCRL* KO H4 cells and i3 neurons. We found a marked decrease in ApoER2 in both OCRL1 deficient cellular models (Figure 3A–C), while ApoER2 mRNA expression in i3 neurons was not modified (Figure S2A). Moreover, ApoER2 surface levels, evaluated by antibody binding in non-permeabilized cells (Figure 3D,E) or cell surface biotinylation (Figure S2B,C), were reduced in OCRL1 deficient cells. In *OCRL* KO i3 neurons, ApoER2 intensity in dendrites (MAP2-positive areas) and the soma were remarkably decreased, but not in the axon (MAP2-negative regions) (Figure 3F–I). As expected for ApoER2 in neurons, nuclear staining should correspond to the receptor intracellular domain [35]. Thus, the loss of OCRL1 reduces ApoER2 total and cell surface levels and its somatodendritic localization.

**Figure 4:**
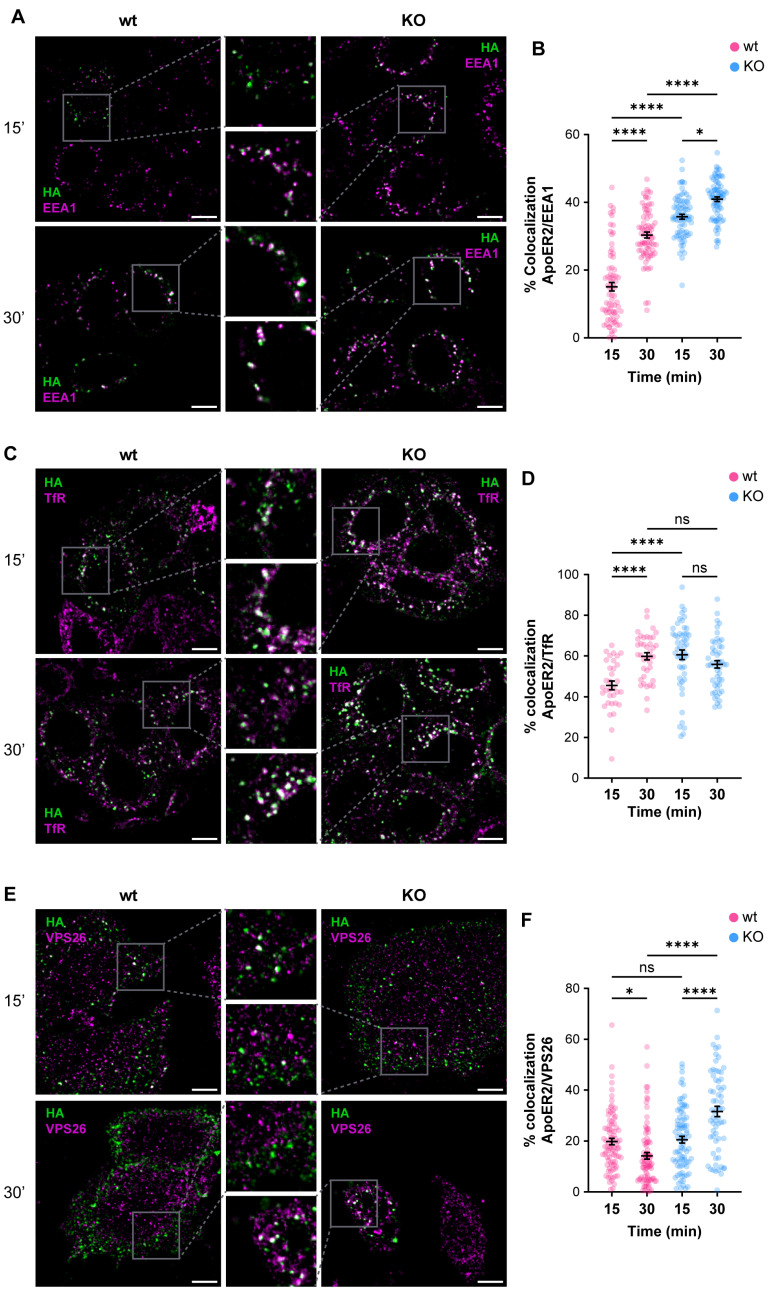
OCRL1 is necessary for ApoER2 intracellular traffic. H4 cells expressing HA-ApoER2 were incubated with anti-HA (green) at 4 °C for 45 min, followed by internalization at 37 °C for indicated times. Cells were acid-washed, fixed, and stained for indicated proteins (magenta): (**A**) EEA1; (**C**) TfR; (**E**) VPS26. Confocal images were deconvolved and analyzed with JACoP from Fiji. Scale bar = 10 µm. (**B**,**D**,**F**) Manders coefficients were analyzed for HA and each protein (corresponding images on the left). ANOVA with Sidak’s multiple comparisons test was calculated from three different experiments. ns: *p* > 0.05, * *p* < 0.05, **** *p* < 0.0001.

Given the alterations of ApoER2 in the absence of OCRL1, we determined if the phosphatase deficiency affects the receptor’s endocytic traffic. Consequently, we measured the colocalization of internalized ApoER2 by following the transfected HA-ApoER2. In wild-type H4 cells, after 15 min of internalization, ApoER2 colocalized less than 20% with EEA1; later (30 min), the percentage increased to 30%. In contrast, in *OCRL* KO cells, the receptor was significantly increased in EEA1-positive structures, compared to the control cells, with 35% and 41% after 15 and 30 min of internalization, respectively (Figure 4A,B).

To better characterize the changes in the intracellular traffic of the receptor upon OCRL1 loss, we colocalized the internalized ApoER2 with TfR, another receptor affected in LS [21], that at steady state is present in early/recycling endosomes. In wild-type H4 cells, 45% of internalized ApoER2 was present in TfR-positive structures after 15 min, whereas at 30 min post internalization, the colocalization increased to 60% (Figure 4C,D). Meanwhile, in *OCRL* KO cells, 60% of ApoER2 colocalized with TfR at 15 min after internalization, and the proportion was maintained at 30 min post-internalization (Figure 4C,D). Moreover, as expected for a recycling receptor [21], the presence of TfR in EEA1-positive endosomes was significantly increased in *OCRL* KO cells (Figure S3A–D).

The Retromer is a physiologically relevant recycling complex responsible for sorting and trafficking different cargoes from the early endosome to the trans-Golgi network and the cell surface [82,83]. We determined the colocalization of internalized ApoER2 with the vacuolar protein sorting-associated protein (VPS26), a specific Retromer complex subunit [84,85]. In wild-type H4 cells, we found that 20% of ApoER2 was present in VPS26-positive structures after 15 min of internalization, whereas the percentage of colocalization decreased to 14% 30 min after internalization (Figure 4E,F). Strikingly, we detected a rise in colocalization of ApoER2 and VPS26 after 30 min of endocytosis (Figure 4E,F) together with increased detection of EEA1 and VPS26 (Figure S3E–H), indicating a loss of identity of the endosomal compartments in the absence of OCRL1 in H4 cells, as was described in kidney cells [22].

So far, our results support the idea that the loss of OCRL1 perturbs the intracellular traffic of receptors, including ApoER2, which accumulates in EEA1- and Retromer-positive endosomes.

**Figure 5:**
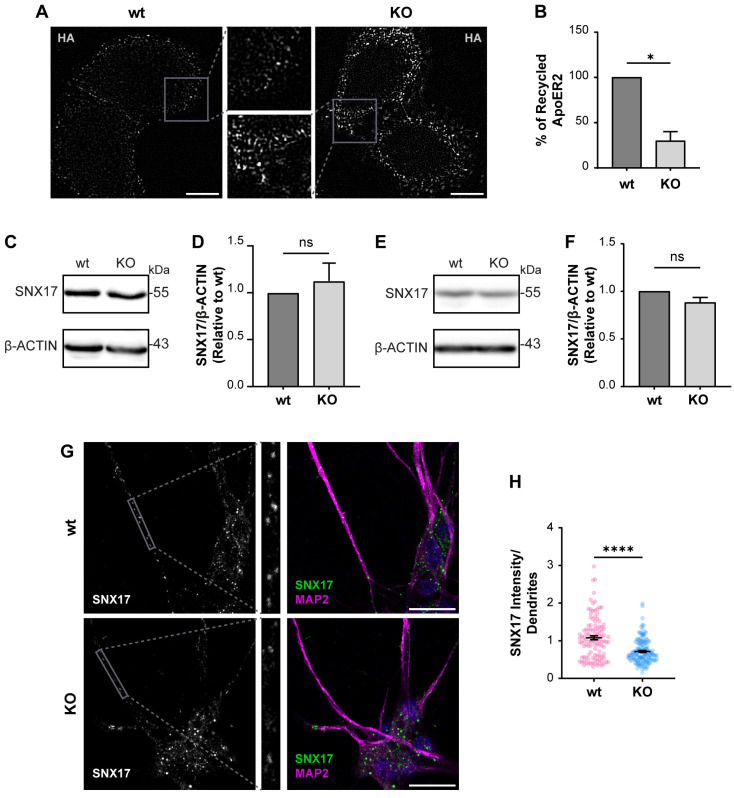
The loss of OCRL1 decreases ApoER2 recycling. (**A**) H4 cells expressing HA-ApoER2 were incubated with anti-HA antibody coupled with Alexa-488 (white) at 4 °C for 45 min; cells were allowed the internalization process by incubating at 37 °C for 30 min, then incubated with anti-Alexa-488 antibody at 4 °C for 45 min to quench non-internalized anti-HA. Cells were allowed to proceed with the recycling process at 37 °C for 30 min, followed by quenching with anti-Alexa-488 antibody at 4 °C for 45 min. Cells were fixed to perform immunodetection. Scale bar = 10 µm. (**B**) The intensities from whole-cell ROIs were measured for internalized and recycled steps from at least 50 cells per experiment. Recycled ApoER2 was calculated as a percentage of the internalized receptor. Statistical significance was calculated using a *t*-test with Welch’s correction. * *p* < 0.05. Cell lysates from wild-type and OCRL1 deficient H4 cells (**C**) and I3 neurons differentiated for two weeks (**E**) were separated by SDS-PAGE followed by western blot to detect endogenous SNX17 (**D**,**F**). The signal intensities corresponding to SNX17 and β-actin were measured by Fiji as the area below the curve for each band. The ratios of SNX17 bands relative to β-actin were normalized to wild-type, plotted, and analyzed with GraphPad. *T*-test was calculated from three independent experiments ns: *p* > 0.05. (**G**) i3 neurons were differentiated for 14 days, fixed, and stained for SNX17 (green), MAP2 (magenta) and DAPI (blue). Scale bar = 20 µm (**H**) SNX17 intensity was measured in dendrites (MAP2-positive structures, gray box) from 25 neurons from two experiments. Statistical significance was calculated using a *t*-test with Welch’s correction. **** *p* < 0.0001. Original images can be found in Supplementary Materials.

Considering what has been described in other OCRL1 deficient cells, we measured the levels of recycled ApoER2 using a chase protocol [41]. We detected a 70% reduction in recycled ApoER2 in KO vs. wild-type cells (Figure 5A,B). A less efficient recycling explains both the increased endosomal localization of the receptor and its reduced cell surface expression. It was previously demonstrated that endosomal-associated protein SNX17 binds to ApoER2, promoting its recycling towards the cell surface [41]. Total levels of SNX17 did not change by western blot in wild-type vs. *OCRL KO* H4 cells (Figure 5C,D), which supports that the recycling impairment is primarily due to alterations caused by the lack of OCRL1. Further, we also analyzed the expression of SNX17, wild-type and OCRL1 deficient i3 neurons, finding that the total levels of SNX17 remain the same (Figure 5E,F). However, in *OCRL* KO i3 neurons, we found a decrease in the intensity of SNX17 in dendrites (Figure 5G,H), suggesting a specific alteration in the dendritic localization of the protein.

**Figure 6:**
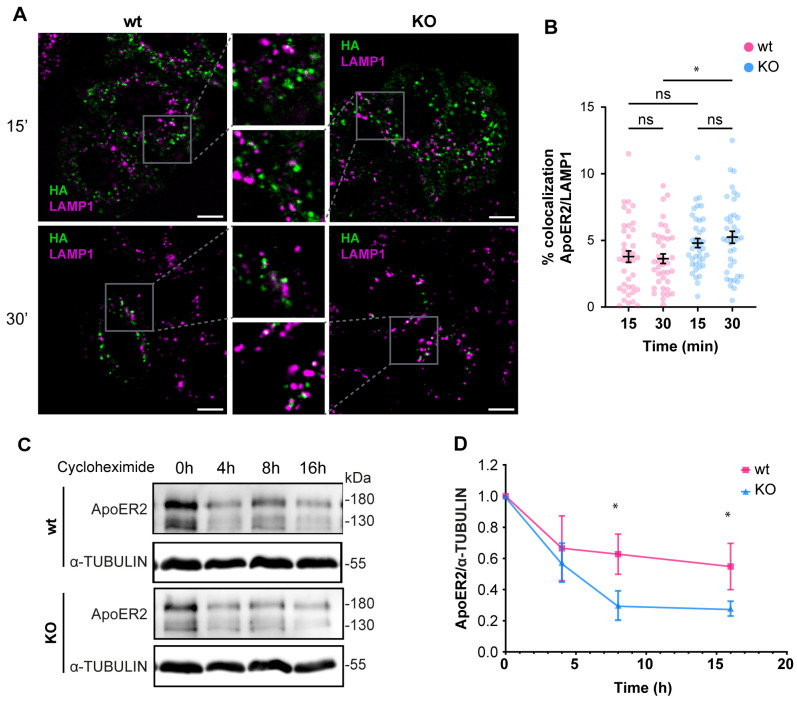
The loss of OCRL1 increases ApoER2 degradation. Wild-type and OCRL1-deficient H4 cells were transfected with a plasmid to express HA-ApoER2. (**A**) After anti-HA (green) internalization, cells were fixed and stained for LAMP1 (magenta). Confocal images were deconvolved and analyzed with Fiji. Scale bar = 10 µm. (**B**) Manders coefficients were analyzed for HA and LAMP1. ANOVA with Sidak’s multiple comparisons test was calculated from two different experiments. ns: *p* > 0.05, * *p* < 0.05. (**C**) To measure the half-life of ApoER2, wild-type, and OCRL1 deficient i3 neurons were differentiated for 21 days and then treated with 25 μM cycloheximide for specified times in a complete cortical medium. Protein samples were subjected to SDS-PAGE and western blot to determine the ApoER2 levels. (**D**) The relative expression of ApoER2, calculated as the ratio of ApoER2 and matching tubulin signals, was normalized to time 0, plotted, and analyzed with GraphPad. ANOVA with Sidak’s multiple comparisons test was calculated for data from three independent experiments. * *p* < 0.05. Original images can be found in Supplementary Materials.

Since the deficiency of OCRL1 induced a defect in recycling and in the protein levels of ApoER2 (Figure 3), we determined if part of the receptor could be diverted to the degradation pathway. For internalized ApoER2, 3% was present in LAMP1-positive structures in wild-type H4 cells. In the *OCRL* KO cells, 5% of the internalized ApoER2 colocalized with LAMP1 after 30 min of internalization (Figure 6A,B), and this difference was significant. It has been shown that in the absence of Reelin as a ligand, most of the receptor recycles and is poorly directed to the degradation pathway, explaining the low presence of ApoER2 in late endosomes/lysosomes [41]. To evaluate the behavior of ApoER2 in a more physiologic model that also expresses endogenous Reelin [67], we determined the receptor half-life in i3 neurons (differentiated for 21 days) using a cycloheximide treatment. ApoER2 protein levels decreased significantly faster in *OCRL* KO than in wild-type i3 neurons, with half-lives of 5 h and 16 h, respectively (Figure 6C,D). Our results suggest that the loss of OCRL1 disrupts ApoER2 intracellular trafficking, affecting its availability at the cell surface and decreasing its half-life, explaining the significant reduction of the receptor in the absence of the phosphatase.

### 3.3. Neuronal Responses to Reelin Are Affected in OCRL KO Cells

**Figure 7:**
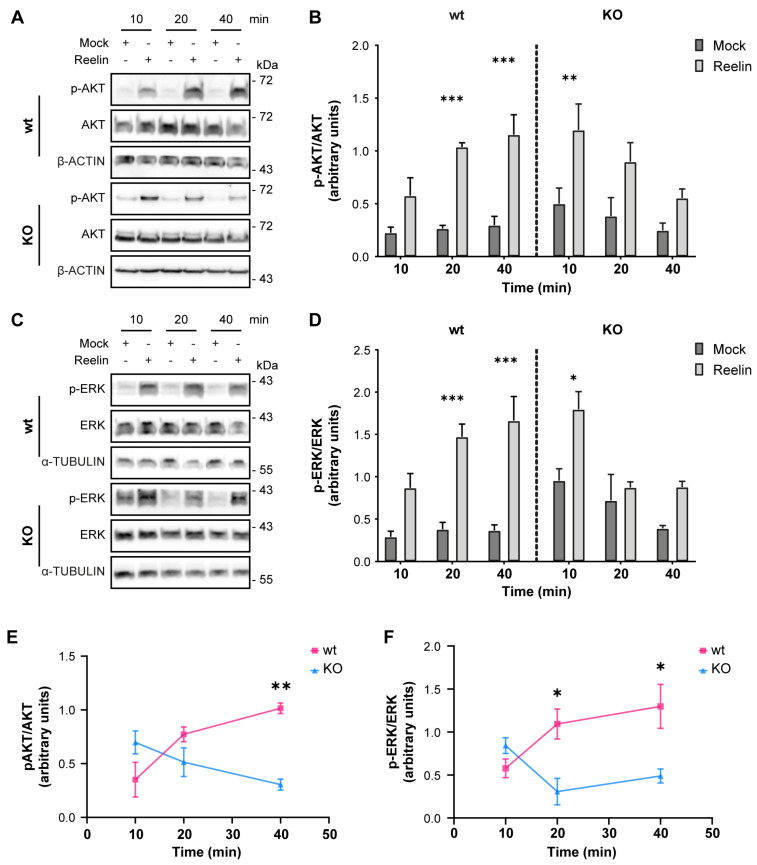
AKT and ERK activation induced by Reelin are decreased in *OCRL* KO i3 neurons**.** Wild-type and OCRL1 deficient i3 neurons were cultured in differentiation media for 21 days, depleted for 1 h, and incubated with 10 nM Reelin or Mock for the indicated times. (**A**,**C**) i3 neurons were lysed with phosphatase inhibitors, and proteins were separated by SDS-PAGE, followed by a western blot. Phosphorylated and total levels of AKT (**A**) and ERK (**C**) were determined (**B**,**D**). Signal intensity was measured with Fiji as the area below the curve. Phosphorylated values were normalized to their respective total levels and plotted. Three-way ANOVA with Tukey’s multiple comparisons test was calculated for three independent experiments * *p* < 0.05, ** *p* < 0.01, *** *p* < 0.001. (**E**,**F**) For each time point, the mock value was subtracted from the Reelin value in (**B**,**D**) to create the curves presented in (**E**,**F**). Two-way ANOVA with Sidak’s multiple comparisons test was calculated from 3 independent experiments * *p* < 0.05, ** *p* < 0.01. Original images can be found in Supplementary Materials.

**Figure 8:**
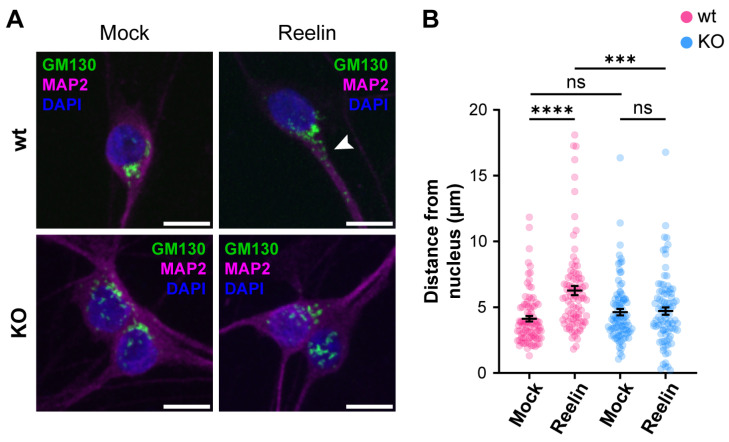
The absence of OCRL1 interferes with ApoER2/Reelin-induced Golgi deployment**.** I3-neurons, wild-type, and *OCRL* KO were differentiated for 12 days, starved for 2 h, and treated with 10 nM Reelin or mock for 30 min. (**A**) Fixed neurons were stained with anti-GM130 (green), anti-MAP2 (magenta), and DAPI (blue). The arrowhead highlights Golgi complex into the dendrite. Scale bar = 10 µm. (**B**) The distance between the outermost Golgi and the nucleus was measured in Fiji using a straight-line tool. At least 50 cells were measured from three experiments. Two-way ANOVA with Sidak’s multiple comparisons test was calculated with GraphPad ns: *p* > 0.05, *** *p* < 0.001, **** *p* < 0.0001.

We previously reported that decreased levels of cell surface ApoER2 led to a reduction of the Reelin/ApoER2 signaling pathway [41,75]. Hence, our next aim was to determine if Reelin responses are affected by the lack of OCRL1. Two branches of the signaling pathway that involve ApoER2 were measured in i3 neurons differentiated for 21 days. For both AKT and ERK activation, we detected significantly reduced responses in the absence of OCRL1 (Figure 7). Furthermore, we evaluated whether the deficiency in OCRL1 affects the deployment of Golgi to the primary dendrite, a developmental relevant ApoER2/Reelin response that depends on the activation of PI3-K and Cdc42 [48]. We found a significant induction of deployment of Golgi in the wild-type neurons (Figure 8). In contrast, Golgi appeared more fragmented in the soma of *OCRL* KO i3 neurons, and Reelin did not induce the entrance of the Golgi membrane into the main MAP2-positive process. Taken together, our results show that OCRL1 is required for ApoER2 functions as a Reelin receptor, suggesting that this signaling pathway could be less active in neurons from LS individuals.

## 4. Discussion

Lowe syndrome is a multisystemic disorder that affects the function of the kidneys, eyes, and brain of the patients [6]. Even though the neurological manifestations can vary in severity among individuals, impacting significantly cognitive abilities and daily functioning of the patients, the precise mechanisms behind these symptoms are still being studied. Here, we report in two human neuronal cellular models that the loss of OCRL1 impacts the intracellular trafficking and signaling function of ApoER2, providing the first documented evidence that links Reelin signaling, essential for CNS development and function, to the pathogenesis of LS.

At the cellular level, the deficiency in OCRL1 causes enlargement of early endosomes [21,22,80,86], persistent perinuclear localization of lysosomes [34], reduction in spreading area [18,25,28], and defective actin dynamics [31,32], possibly reflecting an irregular balance of Rac1/RhoA activation [27,28,87]. Our neuronal models recapitulate the cellular phenotype of LS, with some differences depending on the model.

In OCRL1 deficient H4 cells, we detected a partial but significant alteration in the recycling of ApoER2, explaining its reduction at the cell surface. The impaired recycling is supported by the increased presence of ApoER2 in endosomal compartments labeled by EEA1, TfR, and the Retromer complex. Besides, we found a small but significant increase in ApoER2 in LAMP1-positive late endosomes, which correlates with faster degradation and lower total levels of ApoER2 in the absence of OCRL1.

Different PIPs localized at specific membranes allow the recruitment of various proteins, giving identity to the compartment [88,89]. Accordingly, we report a decrease in the number of EEA1-positive particles in *OCRL* KO i3 neurons, where we also found a decreased presence of the endosomal protein SNX17 in dendrites. SNX17 is the cargo recognition protein associated with the Retriever complex [90]. A significant part of this protein is present in EEA1 endosomes [91,92] since SNX17 binds preferentially to PI3P [93]. Then, a reduction in SNX17 in dendrites with fewer EEA1 endosomes is expected. Furthermore, since SNX17 is needed to recycle ApoER2 and, thus, activate the Reelin-triggered signaling properly [41], the reduction of SNX17 endosomal structures in dendrites may contribute to the decrease in ApoER2 levels and Reelin signaling we detected in LS neurons. Besides, as the SNX17, probably as part of the Retriever, is in charge of recycling other neuronal proteins, including LRP1 [92,94], VLDL-R [95], and APP [96], we speculate that the function of these receptors could be affected in LS due to this pathway. Nevertheless, more specific research is needed to comprehend the effect of the loss of function of OCRL1 in the molecular mechanisms that regulate the sorting of proteins.

The evident alterations in ApoER2 trafficking and protein levels in our neuronal cellular models with loss of OCRL1 indicate a widespread disruption of intracellular traffic with a change in the identity of the endosomal compartments, as we found in kidney cells [22]. OCRL1 deficient cells significantly increased colocalizations of TfR with EEA1 and ApoER2, suggesting that other receptors could be affected in neurons, including Megalin/LRP2, which is also present in CNS and accomplishes relevant roles in the development [97,98,99]. Further, many neuronal cargoes are recycled via the Retromer complex [83]. In OCRL1 deficient cells, we found that the Retromer subunit VPS26 [83,84,85] showed an increased colocalization with internalized ApoER2 and EEA1. Interestingly, SNX27, the endosomal protein in charge of cargo recognition for retrieval with the Retromer complex [100,101], binds to PI3P via its PX domain [93] and to phosphoinositides bi- and tri-phosphates via its FERM domain [102], a domain required for the cargo recycling to the cell surface [103]. In vitro, SNX27 has increased affinity for PI4,5P_2_ and PI3,4,5P_3_ rather than PI3P [104], and it is transiently recruited to the immune synapse by PI3,4,5P_3_ [102]. Since the loss of function of OCRL1 leads to increased PI4,5P2, this could explain the increased presence of the Retromer complex to EEA1 in association with SNX27. Conversely, in other LS models, there is a well-documented alteration of the PIPs from different intracellular compartments [6], affecting the recycling of cargoes to the TGN and the late endosomes/lysosomes for degradation [21].

Regarding the signaling triggered by the binding of Reelin to ApoER2, the AKT-mediated pathway is known to be crucial for dendritic growth and branching [49]. Similarly, OCRL1-deficient kidney cells exposed to insulin have decreased AKT activation [22]. Also, zebrafish embryos deficient in OCRL1 have lower basal AKT activation and total AKT levels [105]. Although we did not measure AKT levels under basal conditions, our results in LS show alterations in this pathway, suggesting a deficiency in Reelin-mediated neuronal development as part of the LS phenotype. In contrast, the same study in zebrafish found no differences in ERK activation and total levels between the LS model and the control [105]. Therefore, unlike the changes observed in AKT, the reduction in ERK signaling could be specific to the response triggered by Reelin, as we recently described in neurons deficient in AP-4 adaptor complex [75], in which ERK and CREB activation, involved in the regulation of synaptic function [106,107] were reduced. Our results show a decrease in ERK activation, so some of the cognitive problems presented by some LS patients may be due to this deficient response.

Another process that we found affected in the OCRL1 deficient neurons is Golgi deployment, a fundamental process for CNS development, as it precedes neuronal polarization and migration [81,108]. It is known that Reelin is capable of activating the Golgi deployment [109] through regulation of the small GTPases Cdc42 and Rac1 modulated by the GEF αPIX [48]. In non-neuronal LS models, an imbalance in the signaling of Rho GTPases has been reported [27,87]. In this line, and unlike the reduced activation of AKT and ERK, Reelin’s ability to induce Golgi deployment was almost absent in OCRL1 deficient neurons; this could imply an evident developmental alteration in LS patients. In this regard, in the *OCRL* KO neurons, we noticed an apparent dispersion or fragmentation of the Golgi complex in the soma. Previous studies in different LS models, but not neurons, have extensively demonstrated a Golgi fragmentation in LS [17,18,26,80], with a strong influence of the 5-phosphatase domain of OCRL1 [17,18]; however, the mechanism and significance for the disease are unclear. Additionally, Golgi mobilization is required for Golgi outpost generation. These compartments are believed to have local roles in microtubule nucleation, protein trafficking, and secretion [109], which would potentially be affected in LS.

All the decreases discussed in ApoER2/Reelin signaling found in human i3 neurons may contribute to understanding some of the neurological signs in LS; as mentioned patients with lissencephaly due to *RELN* mutations have a severe delay in cognitive development, hypotonia, and seizures [64,110]. Moreover, substantial work has linked subtle alterations in ApoER2/Reelin signaling to neuropsychiatric disorders [111,112,113,114]. For instance, the levels of Reelin are severely reduced in postmortem brain extracts from patients with chronic psychosis like schizophrenia and autism, behavioral disorders, and epilepsy [115,116].

Regarding neurodegeneration, murine models of Alzheimer’s disease (AD) show an early decline of Reelin in the hippocampus and cortex [117,118], and human brains of AD patients have lower Reelin levels [117,119]. Several studies have shown that Reelin prevents AD-related dysfunctions [57,63]. At the same time, recently, a *RELN-COLBOS* variant, a gain of function of Reelin, was expressed in a male carrying a *PSEN1* mutation responsible for autosomal dominant AD, delaying the appearance of AD symptoms in 30 years [120].

Besides Reelin, ApoER2 interacts with other ligands with different functions [121]. Remarkably, Apolipoprotein E4 (ApoE4) is an ApoER2 ligand strongly associated with the risk of AD [122]. One of the proposed mechanisms for the influence of ApoE4 on the pathogenesis of AD is the binding of ApoE4 to ApoER2, significantly decreasing its presence in the neuronal membrane, which in turn prevents the interaction of the receptor with Reelin at the synapse [123]. This does not occur if the ligand is ApoE2 or ApoE3 [123]. One of the most critical functions of Reelin at the synapse is the activation of the Src family kinases [124], which in turn phosphorylate the NMDA receptor, decreasing its recycling and strengthening the synapse [125,126]. In this context, our results in LS suggest a similar mechanism of decreased ApoER2 in dendrites, which would partially explain the cognitive and learning problems LS patients face. Then, it is possible to hypothesize that LS patients expressing the ApoE4 isoform could be more prone to developing cognitive impairments. In this context, it was demonstrated that a reversal of the recycling blockage of ApoER2 restores Reelin regulation of synaptic function and signaling [62,127].

Conversely, studies of several mutations of OCRL1 have shown that some patients with the same mutation responsible for LS can develop Dent-2 disease instead, which affects only the kidneys [6]. In this context, while heterozygous polymorphisms in *RELN* alone are insufficient to cause severe problems [128,129,130,131], they may be sufficient to increase the severity of neurological issues in LS patients. In the same direction, transcriptomic analysis of iPSC-derived neuronal progenitors from LS patients shows an increase in Reelin mRNA [132], possibly needed as a compensatory mechanism to activate the signaling since our findings show a direct decrease in ApoER2 [133].

Our work supports the idea that the ApoER2/Reelin pathway is not entirely disrupted yet significantly affected in LS. Although the other Reelin receptor VLDL-R [57] has been less studied in terms of its endocytic trafficking than ApoER2, we cannot discard that its eventual recycling could be impaired by a direct effect of OCRL1 dysfunction at the endosomes as well as by a reduction of SNX17 in dendrites already discussed. These alterations would have an impact on the neurological development of LS patients. It is important to consider that we obtained our results from a complete KO of *OCRL.* However, there are a variety of mutations in OCRL1 causing LS, including nonsense, missense, frameshifts, splice sites, and gross genomic deletions of *OCRL* [6], and the alterations can occur in different domains of the protein [17,18], adding complexity to the phenotype. Therefore, we propose that determining Reelin signaling and ApoER2 levels in iPSCs-derived neurons from LS patients [29,32,132] would be a relevant tool to explain the phenotypic differences in affected individuals.

## Data Availability

Data are contained within the article and Supplementary Materials.

## Supplementary Materials

The following supporting information can be downloaded at https://www.mdpi.com/article/10.3390/biom14070799/s1, File S1: Western Blotting Figures; Table S1: Western blot Antibodies; Table S2: Immunofluorescence Antibodies; Figure S1: Pharmacological inhibition of OCRL1 in rat hippocampal neurons reduces neurite length; Figure S2: mRNA and surface ApoER2 levels in OCRL KO i3 neurons; Figure S3: Loss of OCRL1 disturbs intracellular traffic.
