# Supplementary material for: OCRL1 Deficiency Affects the Intracellular Traffic of ApoER2 and Impairs Reelin-Induced Responses
Source: Biomolecules. 2024 Jul 5;14(7):799. doi: 10.3390/biom14070799 (PMC11274606; doi:10.3390/biom14070799)
Supplement: Supplementary file 1 [file biomolecules-14-00799-s001.zip › biomolecules-3057835-supplementary.pdf]

## SUPPLEMENTAL MATERIALS AND METHODS

*Primary cultures of rat hippocampal neurons*

For primary cultures of rat hippocampal neurons, hippocampi were extracted from rat embryos at E18 and deposited in trypsinization solution (0.05% trypsin, 40 μg/mL DNase I in HBSS). The tissue was incubated for 15 minutes at 37°C. Subsequently, the trypsin was inactivated with an adhesion medium (DMEM, 10% horse serum and 100 U/mL penicillin/streptomycin). The tissue was gently dissociated with Pasteur pipettes, and the obtained cells were counted in a Neubauer chamber. 350,000 cells were seeded onto a 60mm plate with 10 covers coated with poly-lysine. After 2 hours, the medium was changed to Neurobasal medium supplemented with Glutamax, B27, and antibiotics.

*Biotinylation*

Cell surface biotinylation was performed in wild-type and *OCRL* KO human i3 neurons differentiated for 21 days. Cells were washed with ice-cold PBS and then biotinylated using a solution of 0.2 mg/mL biotin (Thermo Fisher, #21335) at 4°C for 15 min. Subsequently, biotin was quenched by incubating the cells twice with 50 mM Tris pH 7.5 and 100 mM NaCl for 10 min on ice. The cells were then lysed with lysis buffer containing PBS, 2% Triton-X100, and protease inhibitors. After centrifugation, the lysates were incubated in rotation at 4°C for 2 h with streptavidin beads (Pierce, #20349) washed with lysis buffer. The beads were washed three times, and any remaining wash buffer was removed before resuspending the beads in the loading buffer. The biotinylated proteins were separated using SDS-PAGE followed by a western blot.

*qPCR*

RNA extraction and qPCR assay were performed as described (Caracci et al., 2024). Human i3 neurons were differentiated for 21 days on poly-L-ornithine-coated 6-well plates (4x105 cells/well). Total RNA was extracted in RNAse-free conditions using TRIzol reagent (Invitrogen) and 1 μg of RNA was reverse transcribed with RevertAid First Strand cDNA Synthesis Kit (Thermo Scientific #K1622). Real-time PCR was performed in a QuantStudio 3 Real-Time-PCR-System (Applied Biosystems, Thermo Fisher Scientific) using Hot FirePol Evagreen qPCR Mix, ROX (Solis Biodyne, #08-24-00001) with 200nM of ApoER2 primers. The qPCR conditions were denaturation at 95 °C for 15 s, annealing at 55 °C for 30 s and extension at 72 °C for 30 s. The expression levels of ApoER2 were normalized to GAPDH expression using the delta–delta Cq method (2−ΔΔCq).

**Supplemental Table S1: Western blot Antibodies**

| **Antibody** | **Host** | **Dilution** | **Catalog** | **Source** |
|---|---|---|---|---|
| OCRL-1 | Rabbit | 1:1500 | - | Polyclonal antibody against human OCRL was described before (Vicinanza et al., 2011)[21] |
| $\alpha$-Tubulin | Rat | 1:20000 | ab6160 | Abcam |
| β-actin-HRP | Mouse | 1:20000 | ab49900 | Abcam |
| ApoER2 | Rabbit | 1:5000 | A3481 | Sigma-Aldrich |
| Phospho-Akt (Ser473) | Rabbit | 1:1000 | OMA103061 | Thermo Fisher |
| Akt (pan) | Mouse | 1:1000 | 2920S | Cell Signalling |
| Phospho-p44/42 MAPK | Rabbit | 1:2000 | 9101S | Cell Signalling |
| ERK (pan) | Mouse | 1:1000 | 610124 | BD Biosciences |

**Supplemental Table S2: Immunofluorescence Antibodies**

| Antibody | Host | Dilution | Catalog | Source |
|---|---|---|---|---|
| EEA1 | Mouse | 1:500 | 610456 | BD Biosciences |
| Lamp1 | Mouse | 1:500 | H4A3 | DSHB |
| HA | Chicken | 1:500 | AB3254 | Millipore |
| HA | Rabbit | 1:1000 | C29F4 | Cell Signalling |
| ApoER2 | Rabbit | 1:3000 | A3481 | Sigma-Aldrich |
| MAP2 | Chicken | 1:5000 | ab5392 | Abcam |
| Ankyrin-G | Mouse | 1:100 | sc12719 | Santa Cruz |
| TfR | Mouse | 1:500 | G1/221/12 | DSHB |
| SNX17 | Rabbit | 1:100 | HPA043867 | Protein Atlas |
| VPS26 | Rabbit | 1:1000 | 1280-1-AP | Proteintech |
| GM130 | Mouse | 1:500 | 610822 | BD Biosciences |
| Phalloidin-Alexa Fluor 647 | - | 1:1000 | A22287 | Thermo Fisher |
| HA-Alexa Fluor 488 | Goat | 1:100 | A21287 | Thermo Fisher |
| Anti-mouse-IgG-Alexa Fluor 647 | Goat | 1:1000 | A21235 | Thermo Fisher |
| Anti-mouse-IgG-Alexa Fluor 555 | Donkey | 1:1000 | A31570 | Thermo Fisher |
| Anti-chicken-IgG-Alexa Fluor 647 | Goat | 1:1000 | A21436 | Thermo Fisher |
| Anti-chicken-IgG-Alexa Fluor 488 | Goat | 1:1000 | A11039 | Thermo Fisher |
| Anti-rabbit-IgG-Alexa Fluor 555 | Donkey | 1:1000 | A31572 | Thermo Fisher |

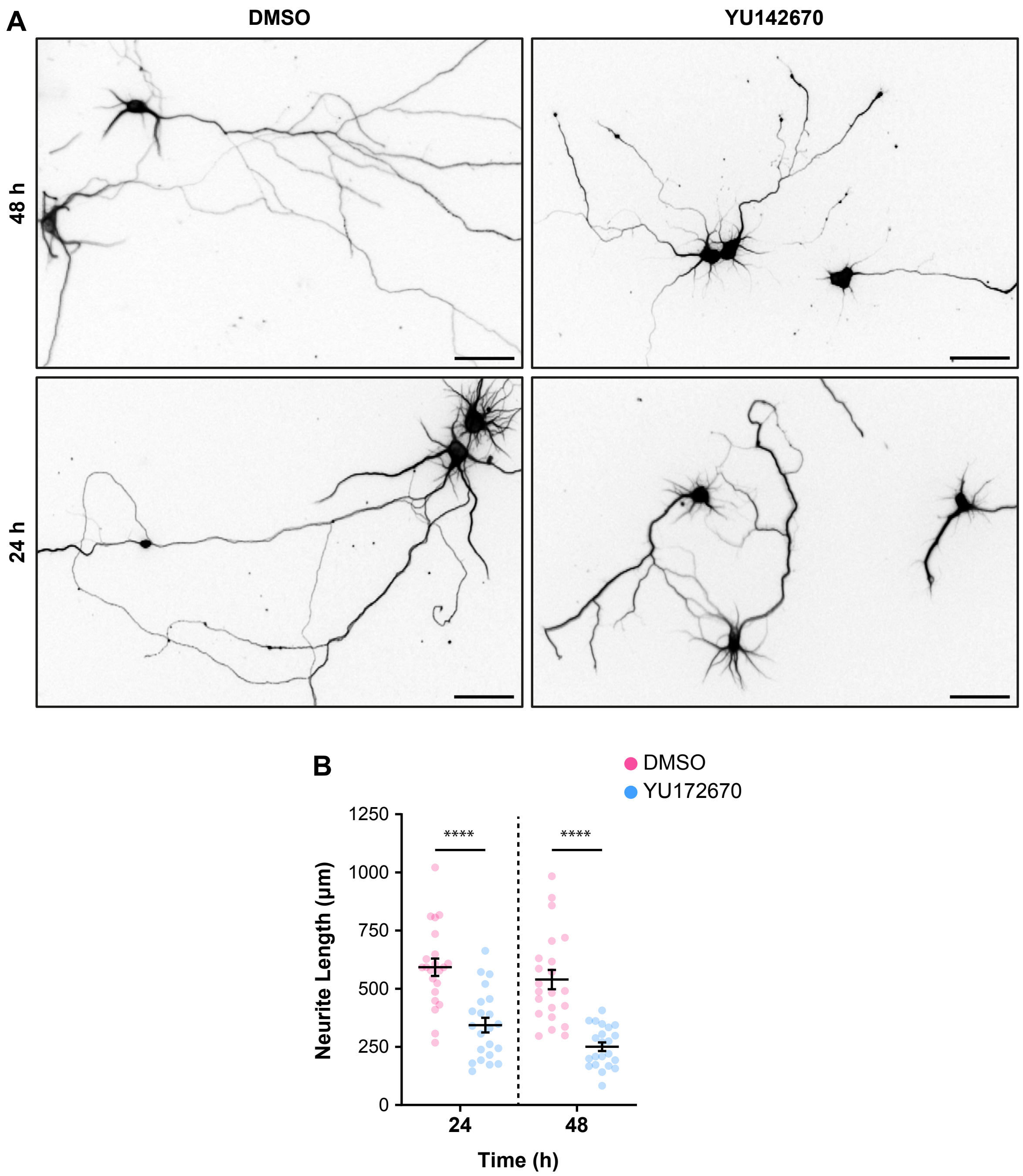


**Figure S1. Pharmacological inhibition of OCRL1 in rat hippocampal neurons reduces neurite length.** Primary cultures of rat embryonic (E18) hippocampal neurons were seeded on coverslips coated with poly-L-lysine. (**A**) After 3 days in vitro, neurons were treated with DMSO or 50uM YU1426701 for 24 or 48 h, fixed and stained for βIII tubulin. Images were captured using a wide-field microscope and analyzed with Fiji. Scale bar = 50 µm. (**B**) Neurite length was measured from βIII tubulin signal using Neuron J. Data from 22 neurons cells from 3 experiments were analyzed, and statistical significance was calculated using ANOVA with Sidak's multiple comparisons test. **** $p < 0.0001$.

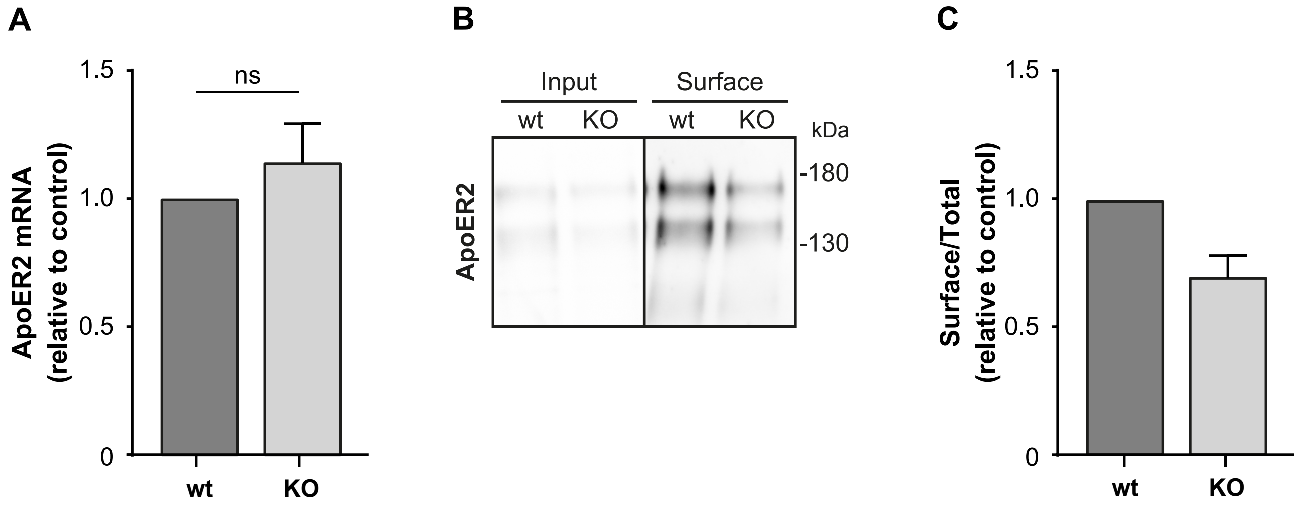


**Figure S2. mRNA and surface ApoER2 levels in *OCRL* KO i3 neurons.** (**A**) Relative expressions of ApoER2 mRNA from wt and *OCRL* KO i3 neurons differentiated for 21 days. Expression levels were normalized to GAPDH expression using the delta-delta Cq method (2-ΔΔCq). Data are presented as the mean±SEM, n=3, Mann-Whitney t-test.Biotinylation was performed after 21 days of differentiation. (**B)** i3 neurons (wt or *OCRL* KO) were biotinylated at 4ºC for 15 min. Proteins bound to biotin were separated with streptavidin beads. Input and Surface proteins were analyzed by western blot. (**C**) The signal from ApoER2 was measured with Fiji, and the ratio of surface vs input was determined and normalized to wt. Since only two experiments were performed, no statistic analysis was calculated.

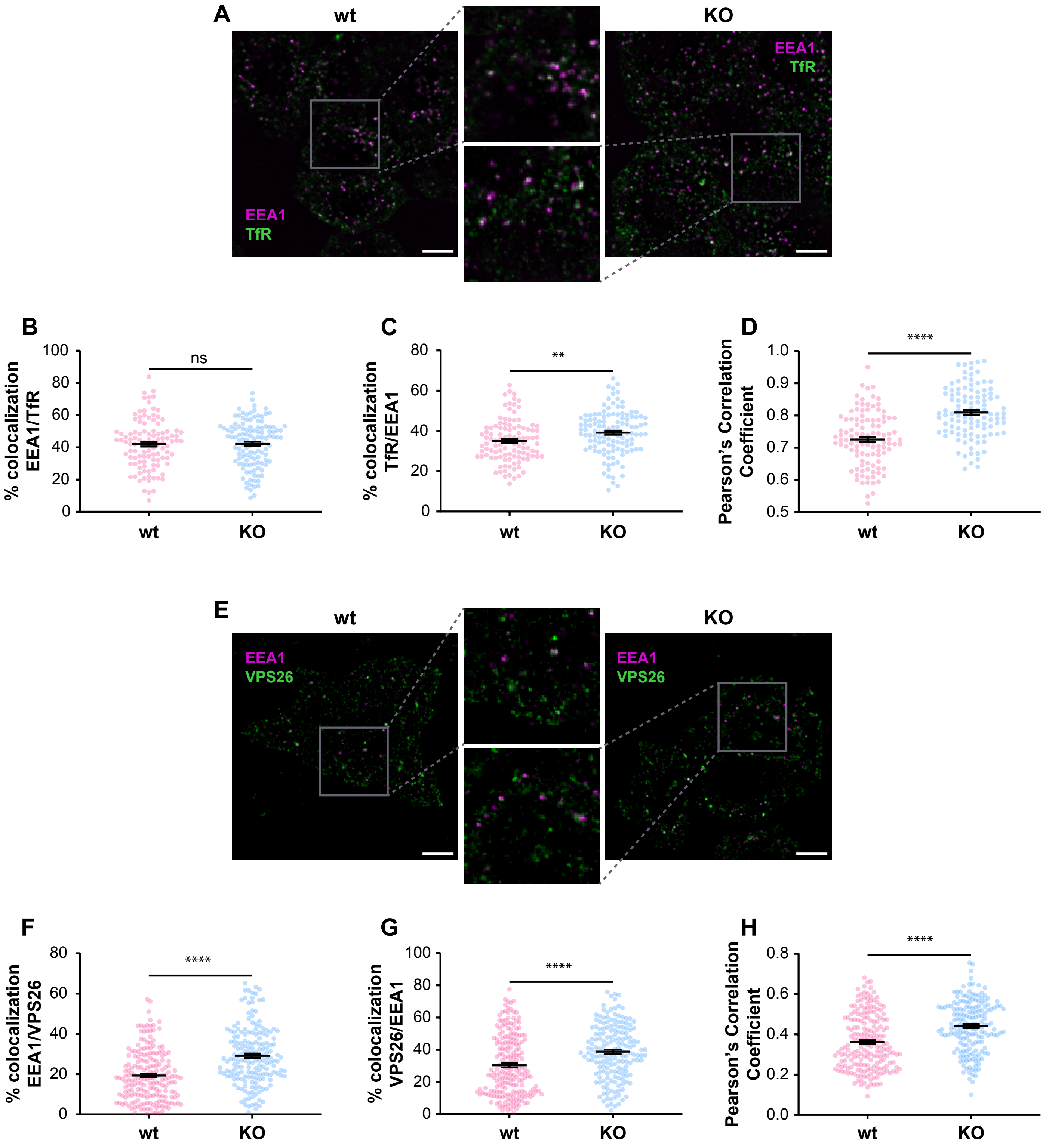


**Figure S3. Loss of OCRL1 disturbs intracellular traffic.** H4 cells were fixed, and stained for EEA1 (magenta) and indicated proteins (green): (**A**) Transferrin Receptor (TfR); (**E**) VPS26. Confocal images were deconvolved and analyzed with ImageJ. Scale bar indicates 10 μm. (**B, C, F, G**) Manders coefficients were analyzed for EEA1 and each protein (corresponding images above). (**D, H**) Pearson's Correlation Coefficient (PCC) were analyzed for EEA1 and each protein (corresponding images above). Error lines represent mean±SEM. t-student's test was calculated from 100 cells from 2 experiment. ns $p > 0.05$, ** $p < 0.01$, **** $p < 0.0001$.
