# Supplementary material for: OCRL1 Deficiency Affects the Intracellular Traffic of ApoER2 and Impairs Reelin-Induced Responses
Source: Biomolecules. 2024 Jul 5;14(7):799. doi: 10.3390/biom14070799 (PMC11274606; doi:10.3390/biom14070799)

OCRL1 (upper) and tubulin (lower)
H4 cells, figure 1A

1. MW St
2.
3.
4.
5. H4 wt showed in figure 1A
6. H4 OCRL KO showed in figure 1A
7.
8. MW St

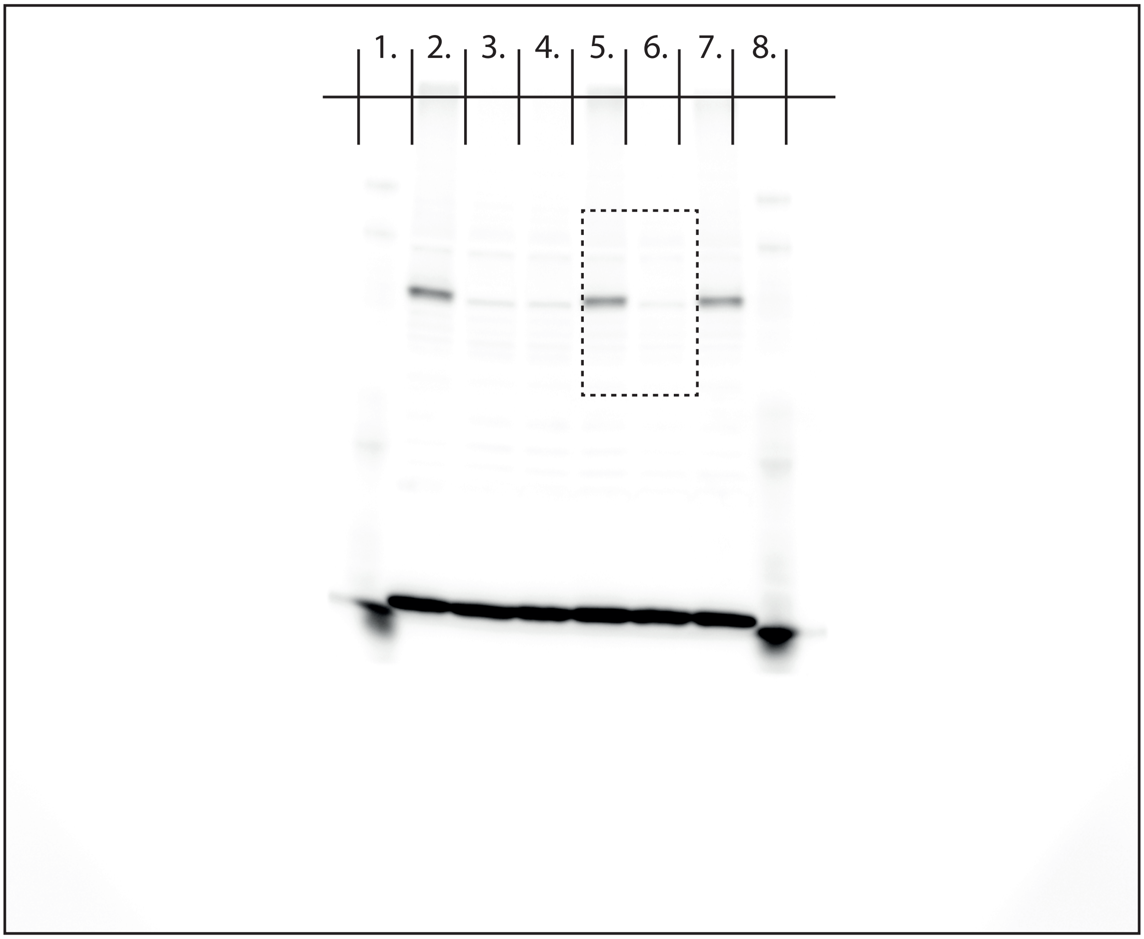


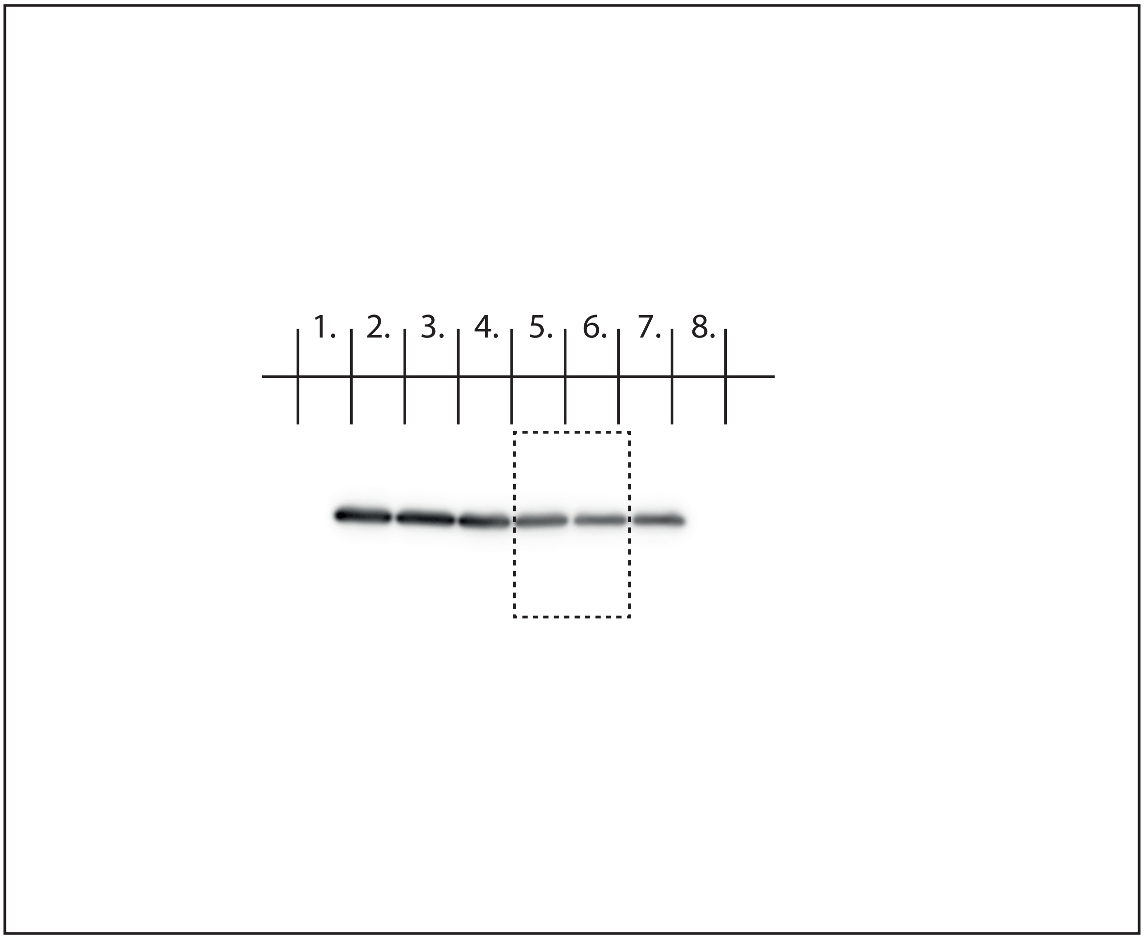

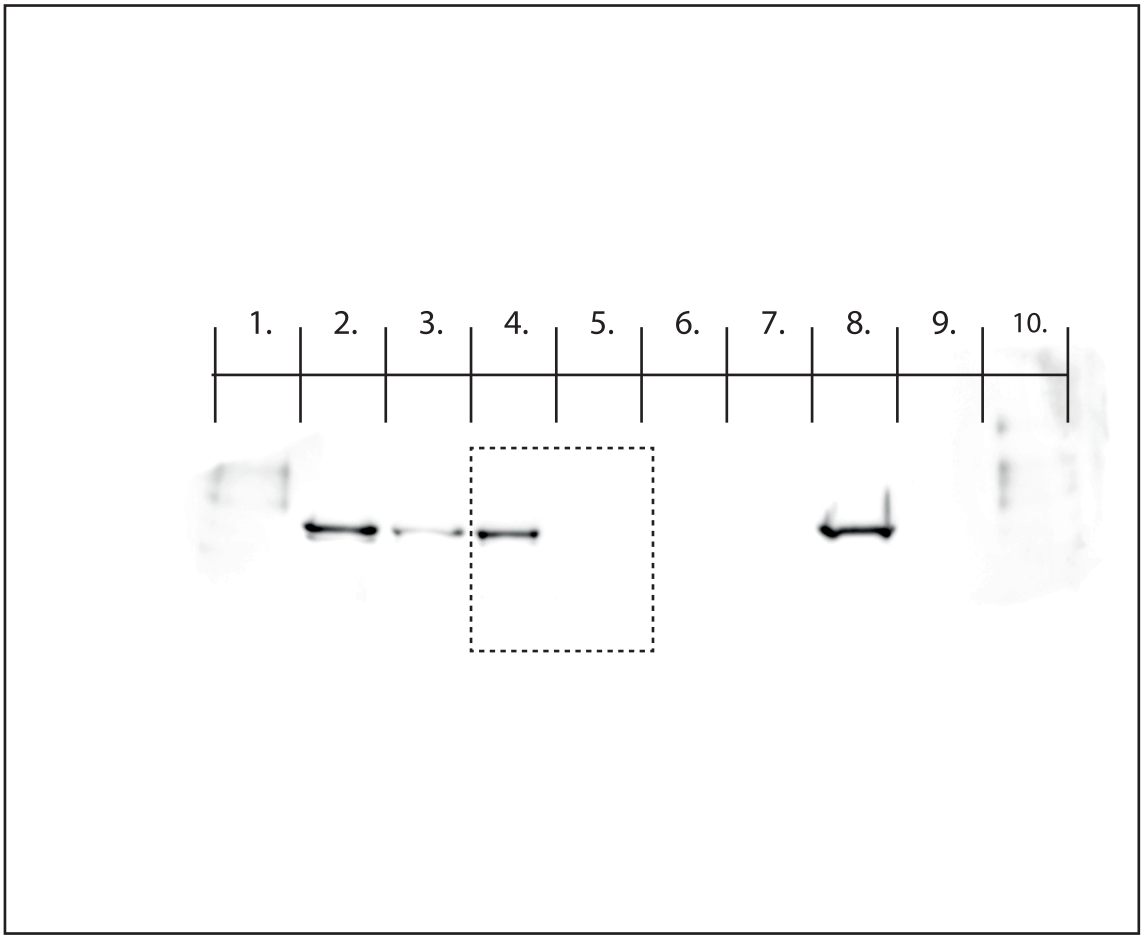


OCRL1 (upper) and tubulin (lower)
iPSC, figure 2A (left)

1. MW St
2.
3.
4. iPSC wt showed in figure 2A (left)
5. iPSC OCRL KO showed in figure 2A (left)
6.
7.
8.
9.
10. MW St

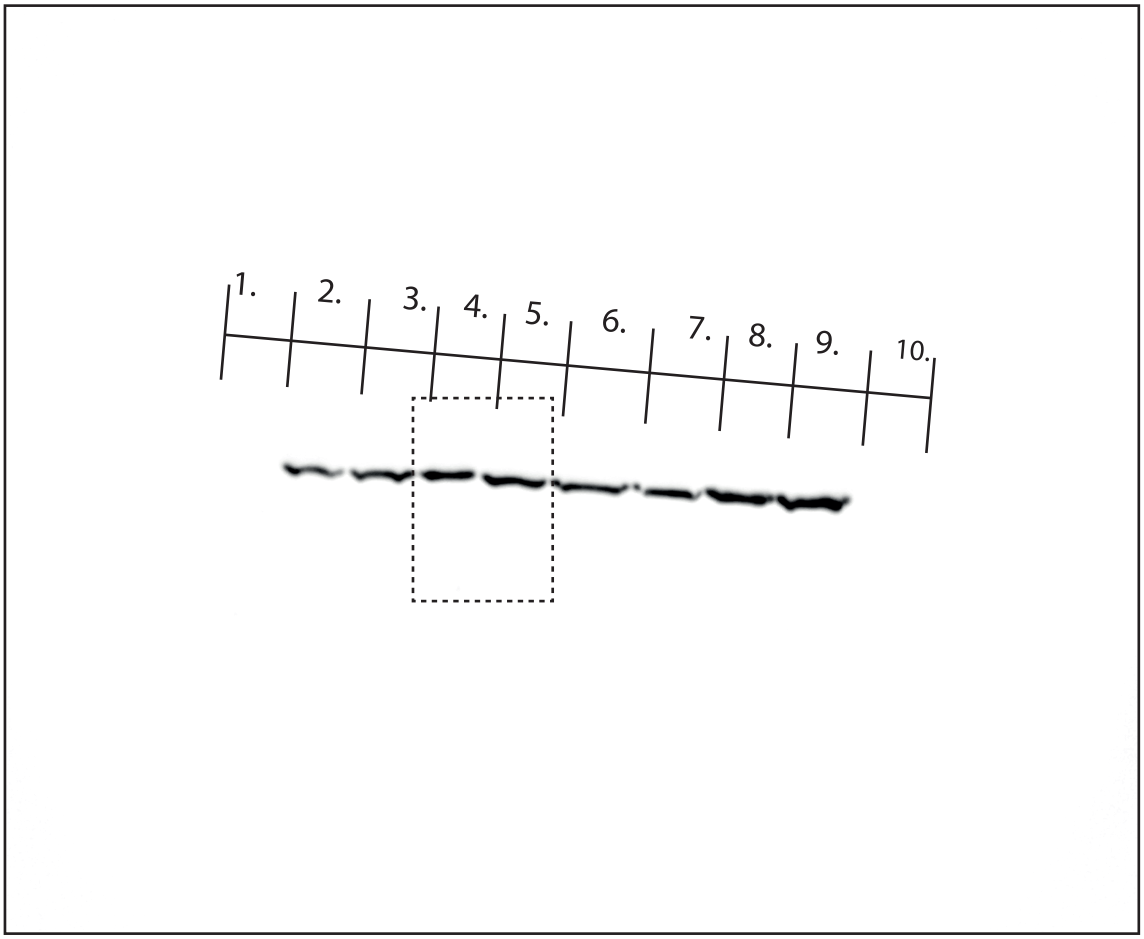

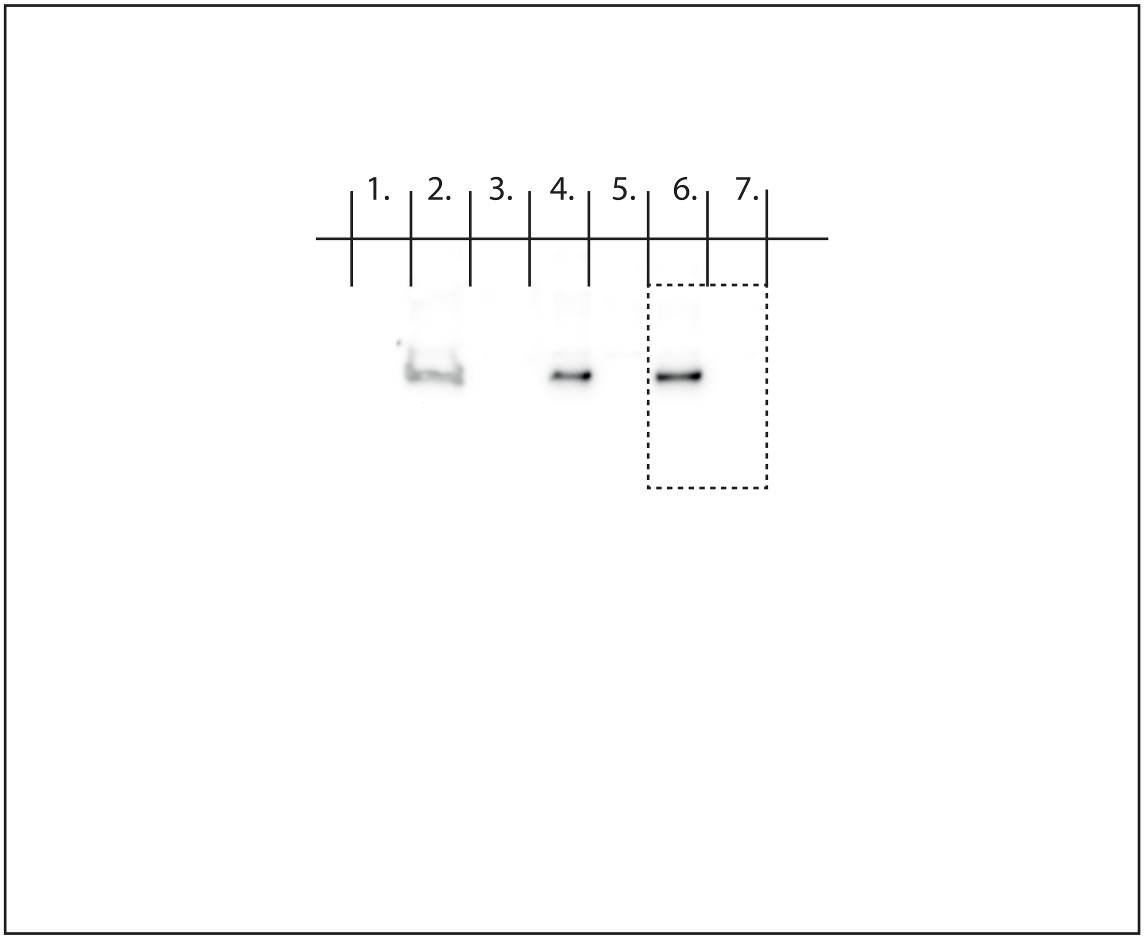


OCRL1 (upper) and tubulin (lower)
i3 neurons, figure 2A (right)

1. MW St
2.
3.
4.
5.
6. i3 neurons wt showed in figure 2A (right)
7. i3 neurons OCRL KO showed in figure 2A (right)

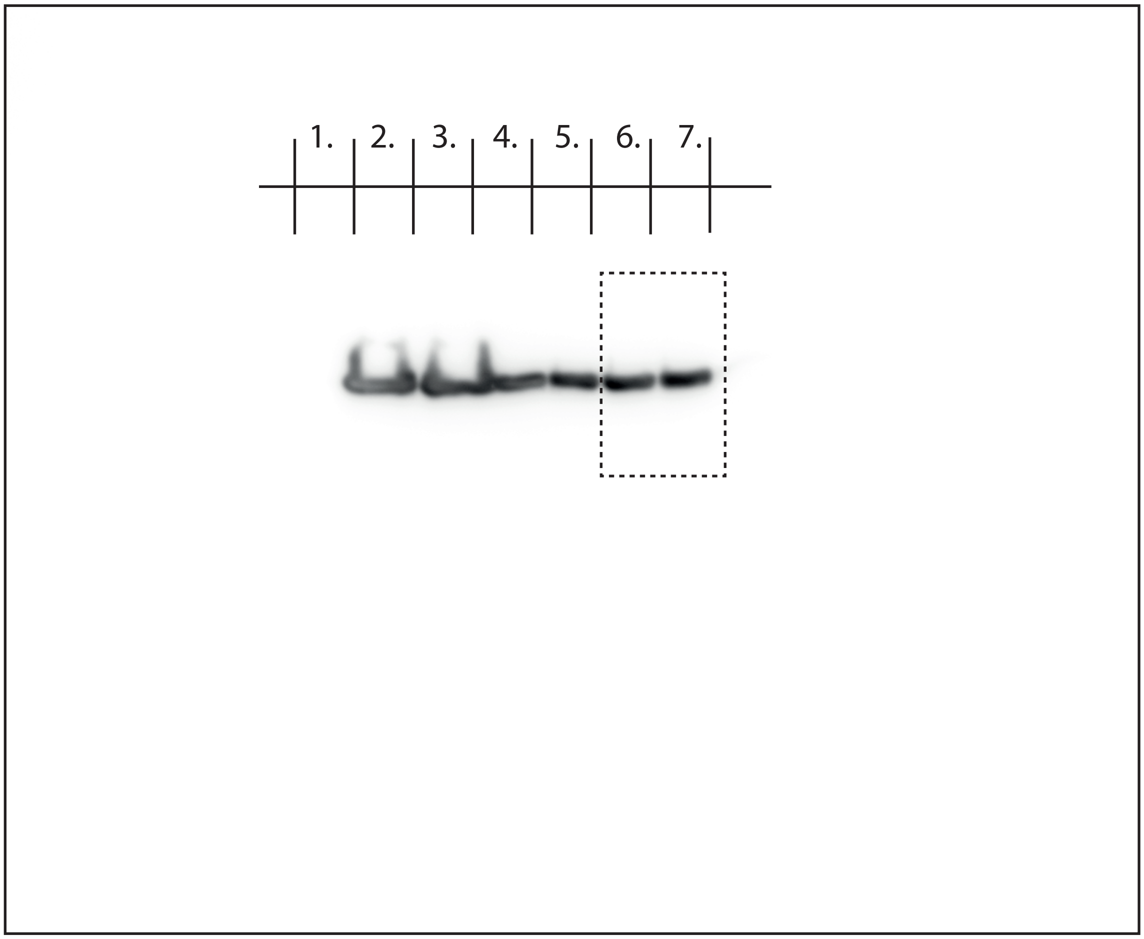

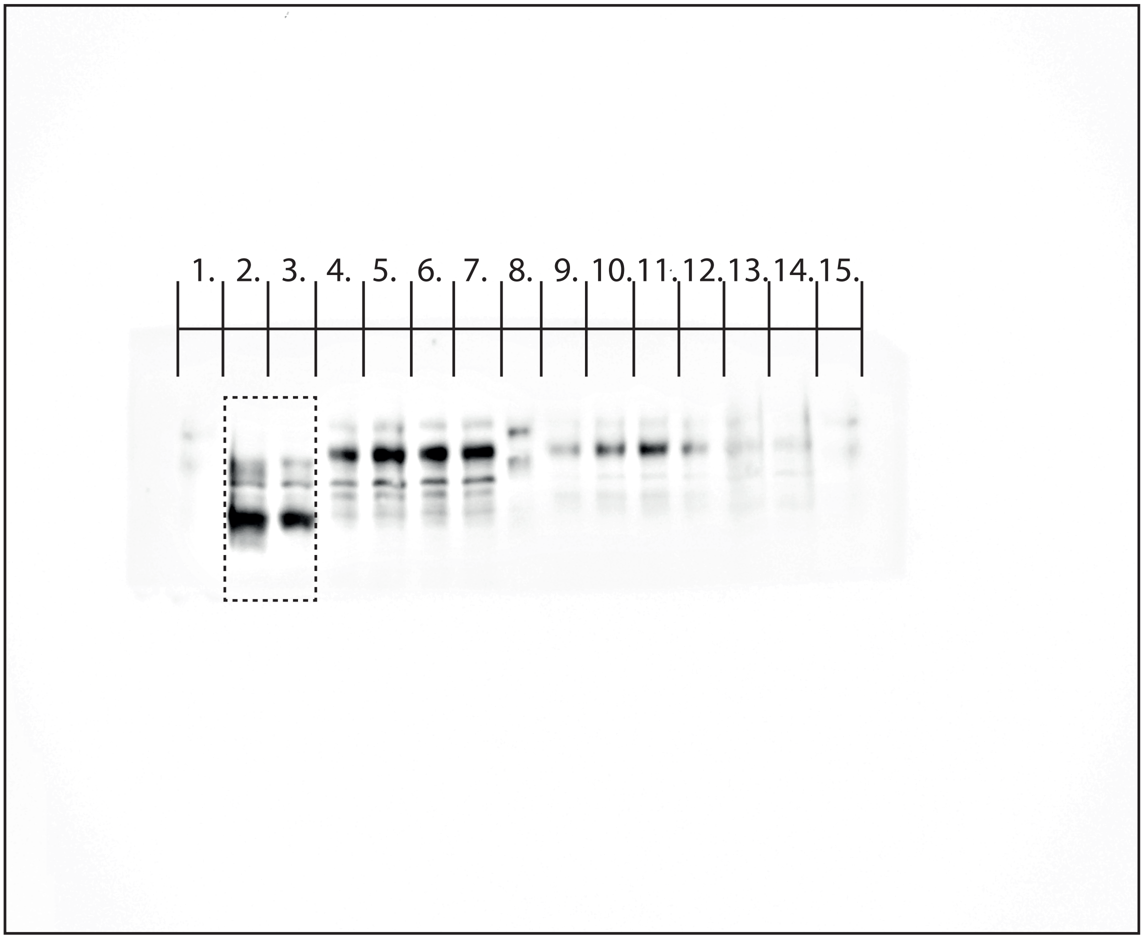


ApoER2 (upper) and tubulin (lower)
H4 cells, figure 3A (left)

1. MW St + irrelevant sample
2. H4 wt showed in figure 3A (left)
3. H4 OCRL KO showed in figure 3A (left)
4.
5.
6.
7.
8. MW St + irrelevant sample
9.
10.
11.
12.
13.
14.
15.

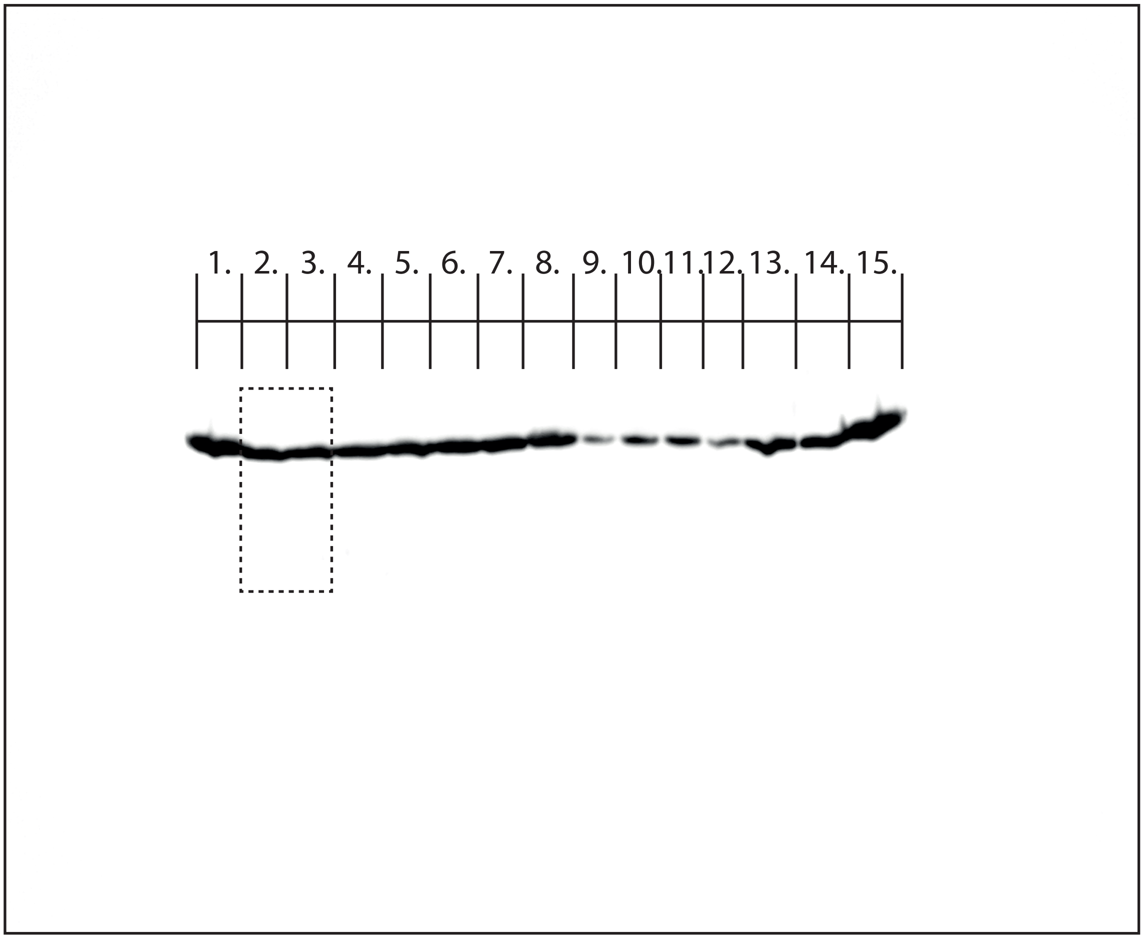

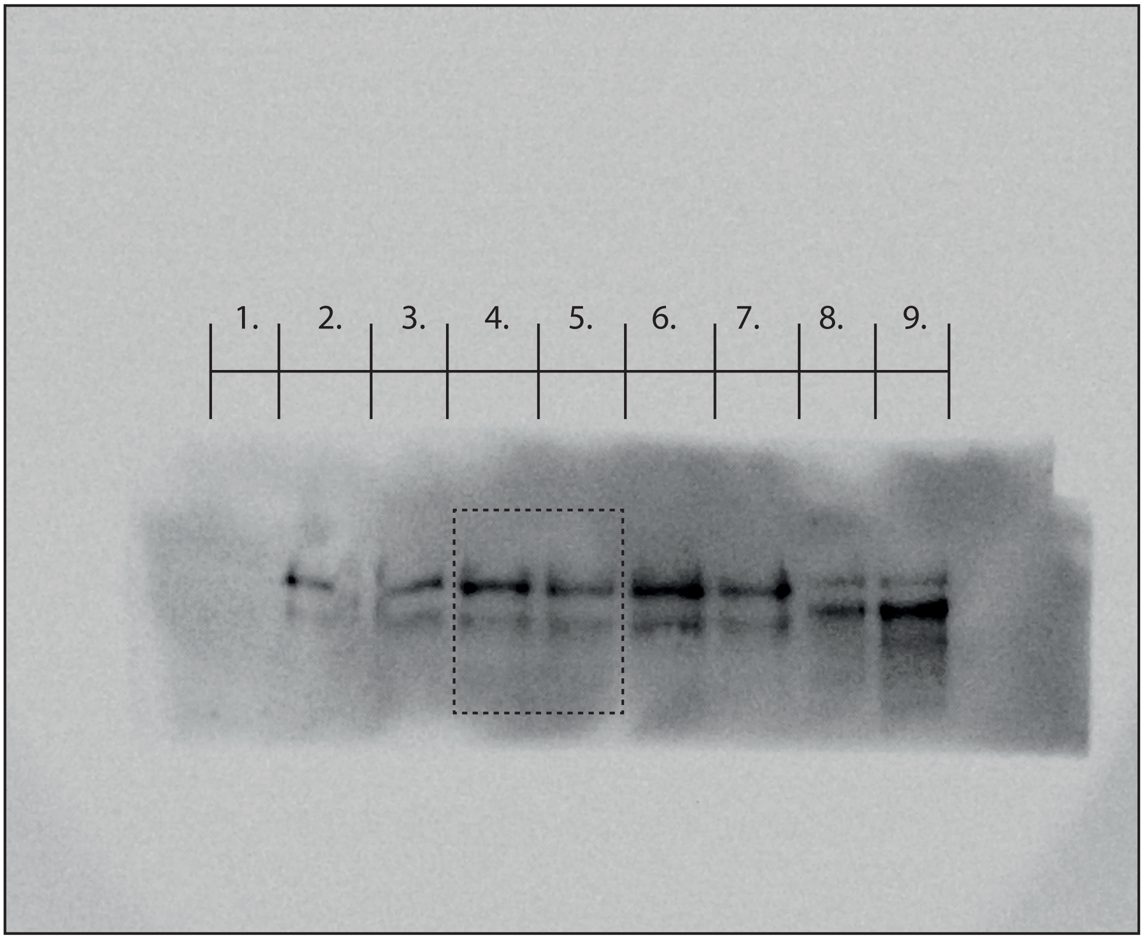


ApoER2 (upper) and tubulin (lower)
i3 neurons, figure 3A (right)

1. MW St
2.
3.
4. i3 neurons wt showed in figure 3A (right)
5. i3 neurons OCRL KO showed in figure 3A (right)
6.
7.
8.
9.

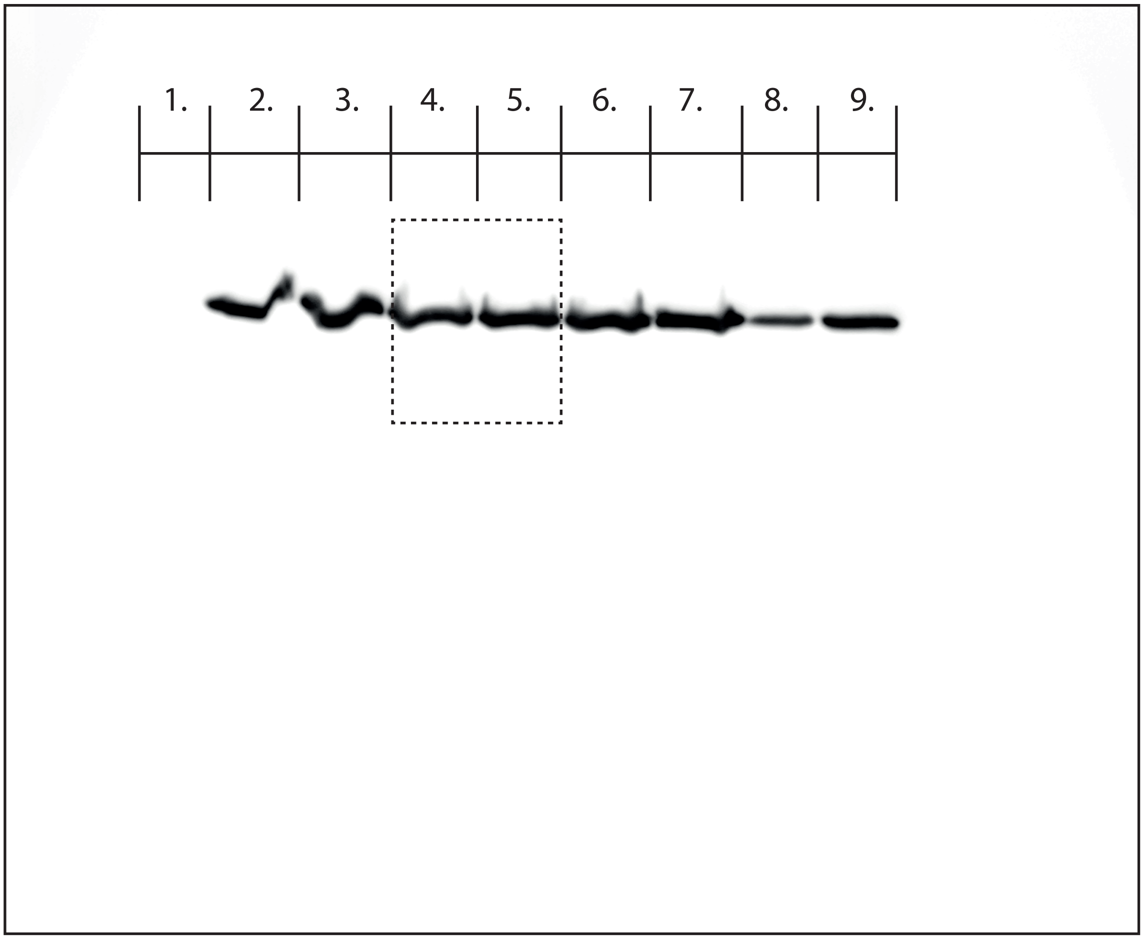

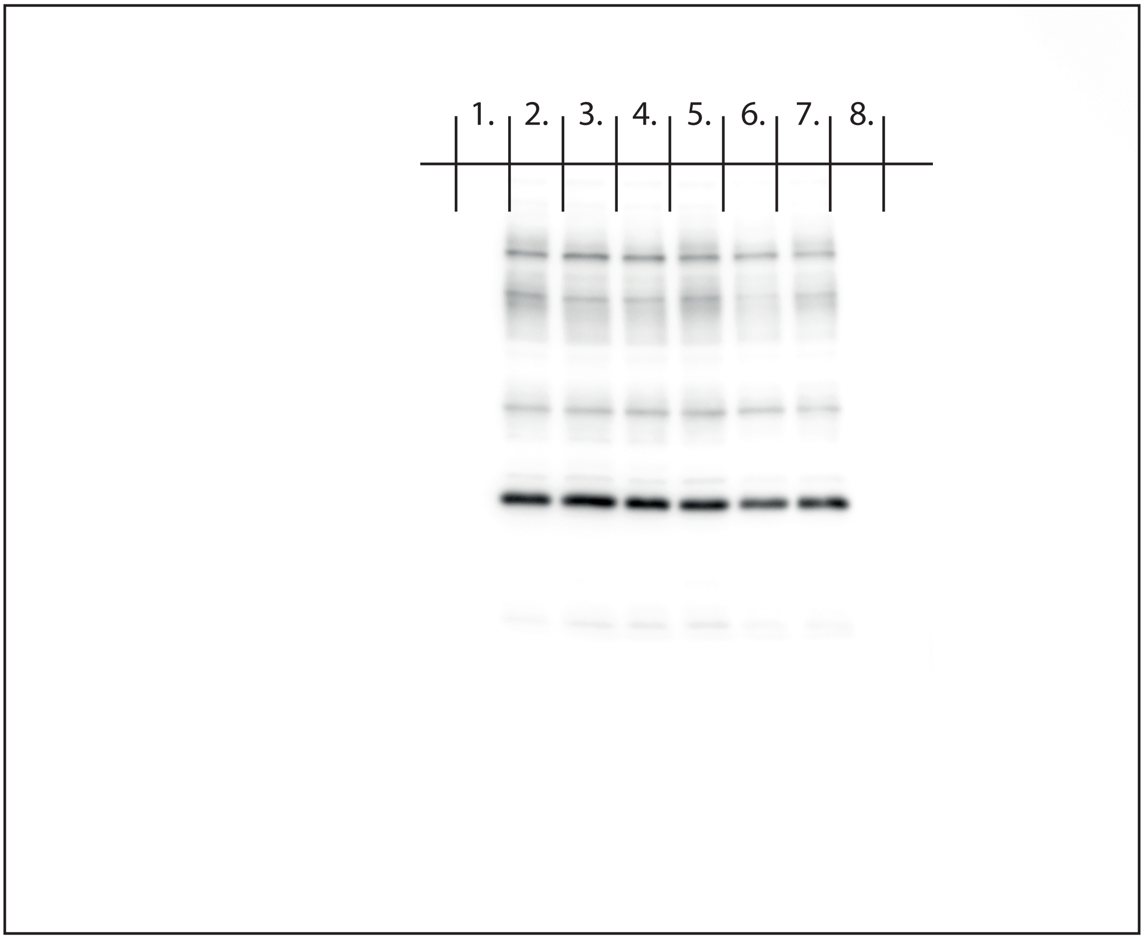


ApoER2 (upper) and tubulin (lower)
H4 cells, figure 3B (chart)

1. MW St
2.
3.
4.
5. H4 wt analyzed in figure 3B (experiment number 1)
6. H4 OCRL KO analyzed in figure 3B (experiment number 1)
7.
8. MW St

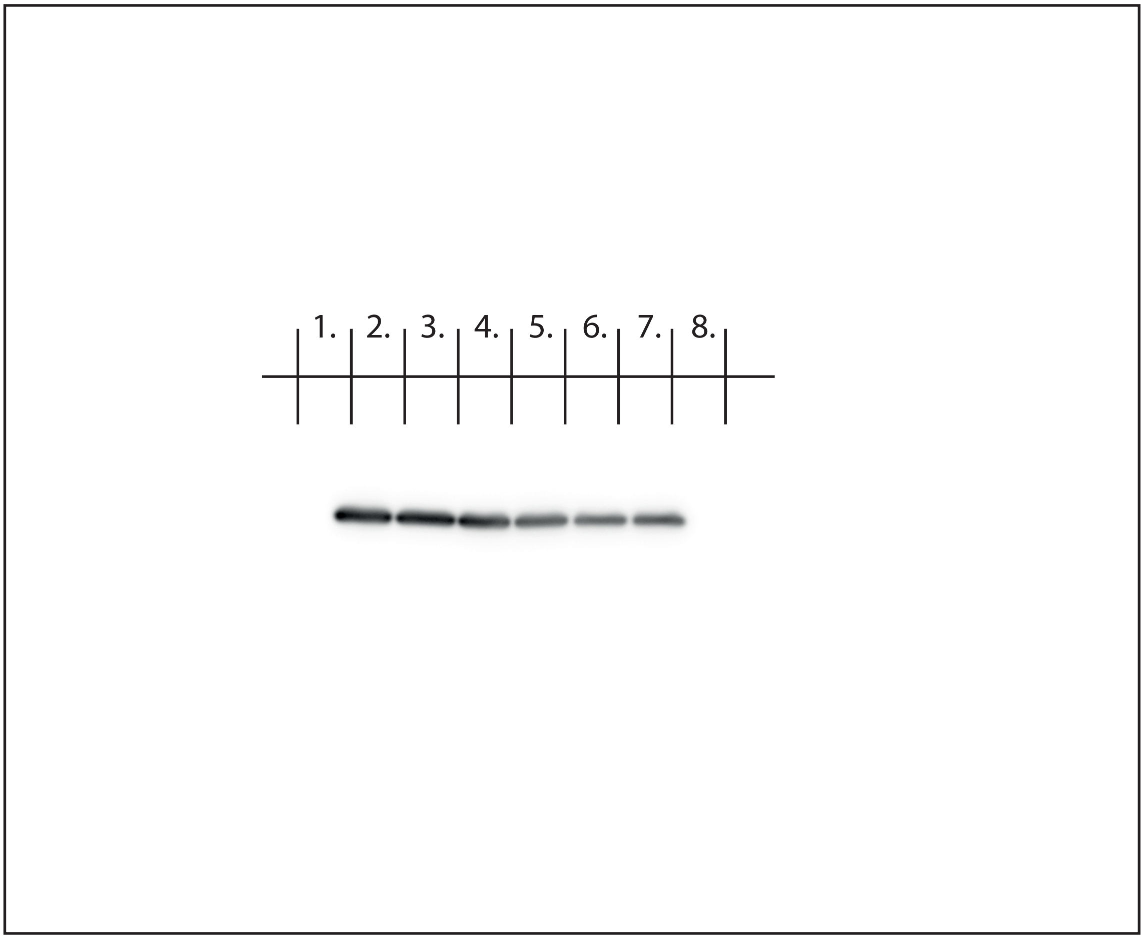

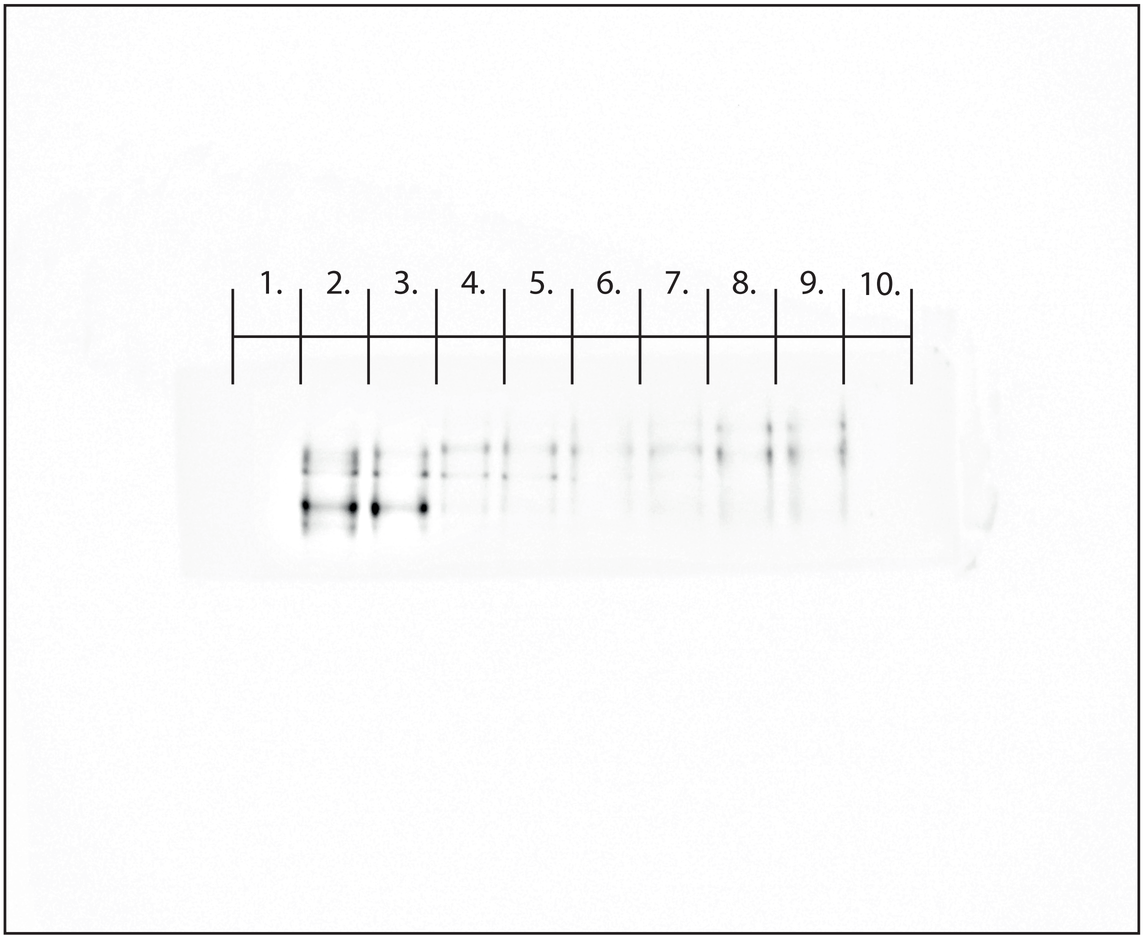


ApoER2 (upper) and tubulin (lower)
H4 cells, figure 3B (chart)

1. MW St
2.H4 wt analyzed in figure 3B (experiment number 2)
3. H4 OCRL KO analyzed in figure 3B (experiment number 2)
4.
5.
6.
7.
8.
9.
10. MW St

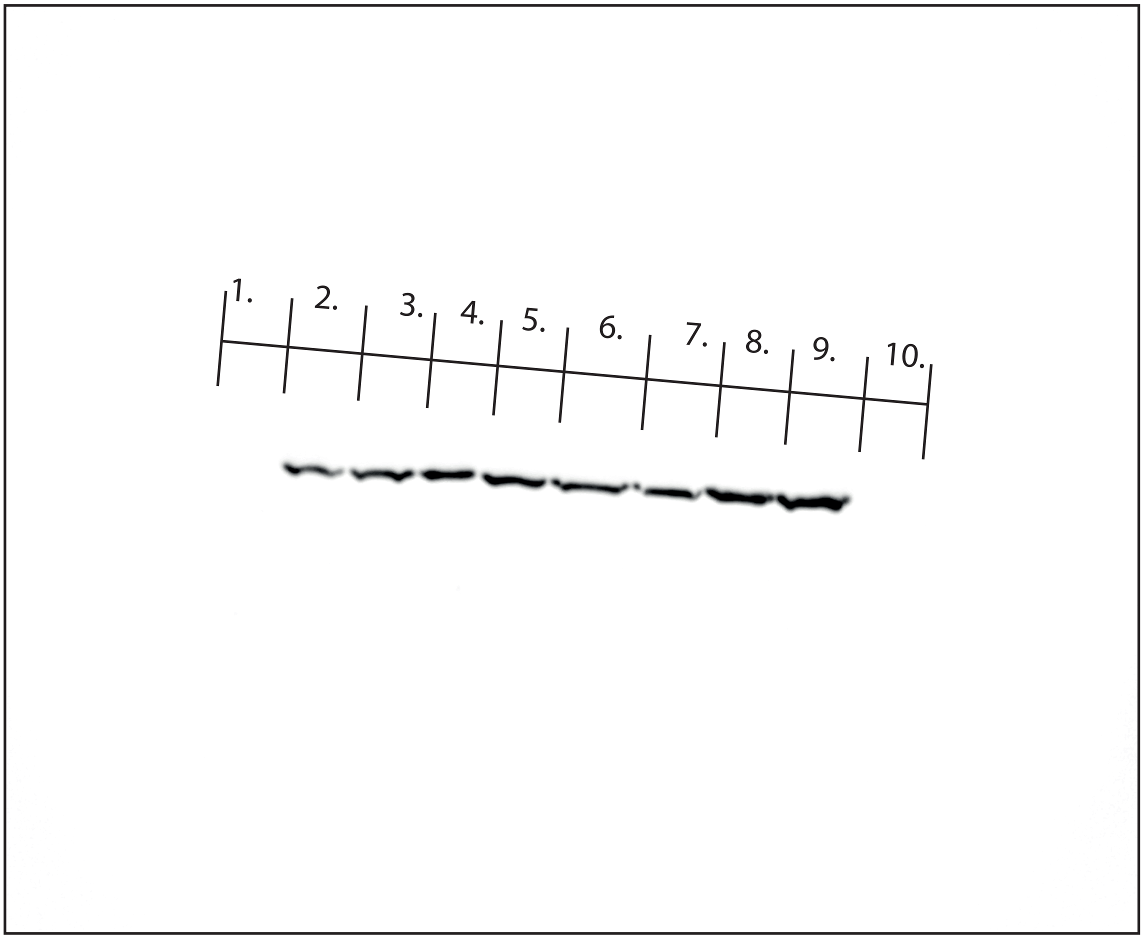

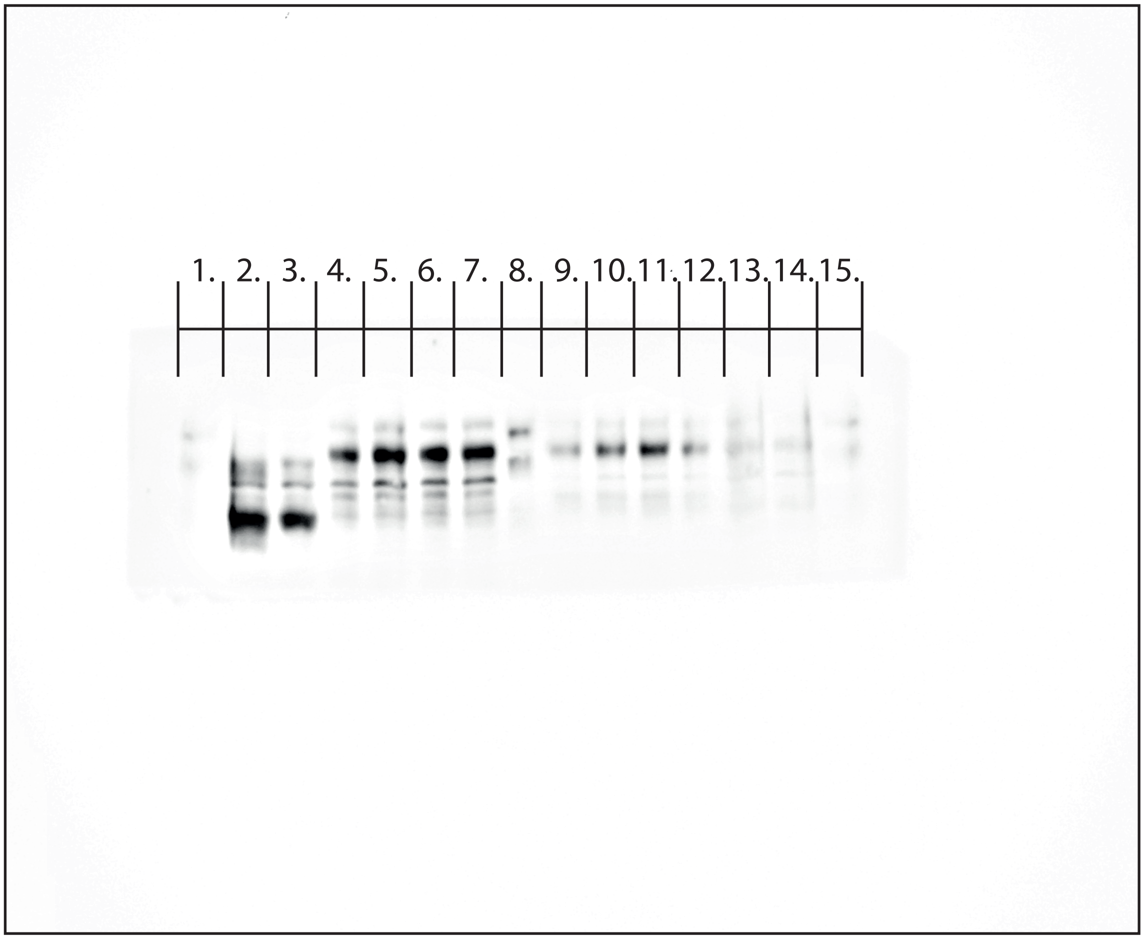


ApoER2 (upper) and tubulin (lower)
H4 cells, figure 3B (chart)

1. MW St + irrelevant sample
2. H4 wt analyzed in figure 3B (experiment number 3)
3. H4 OCRL KO analyzed in figure 3B (experiment number 3)
4.
5.
6.
7.
8. MW St + irrelevant sample
9.
10.
11.
12.
13.
14.
15.

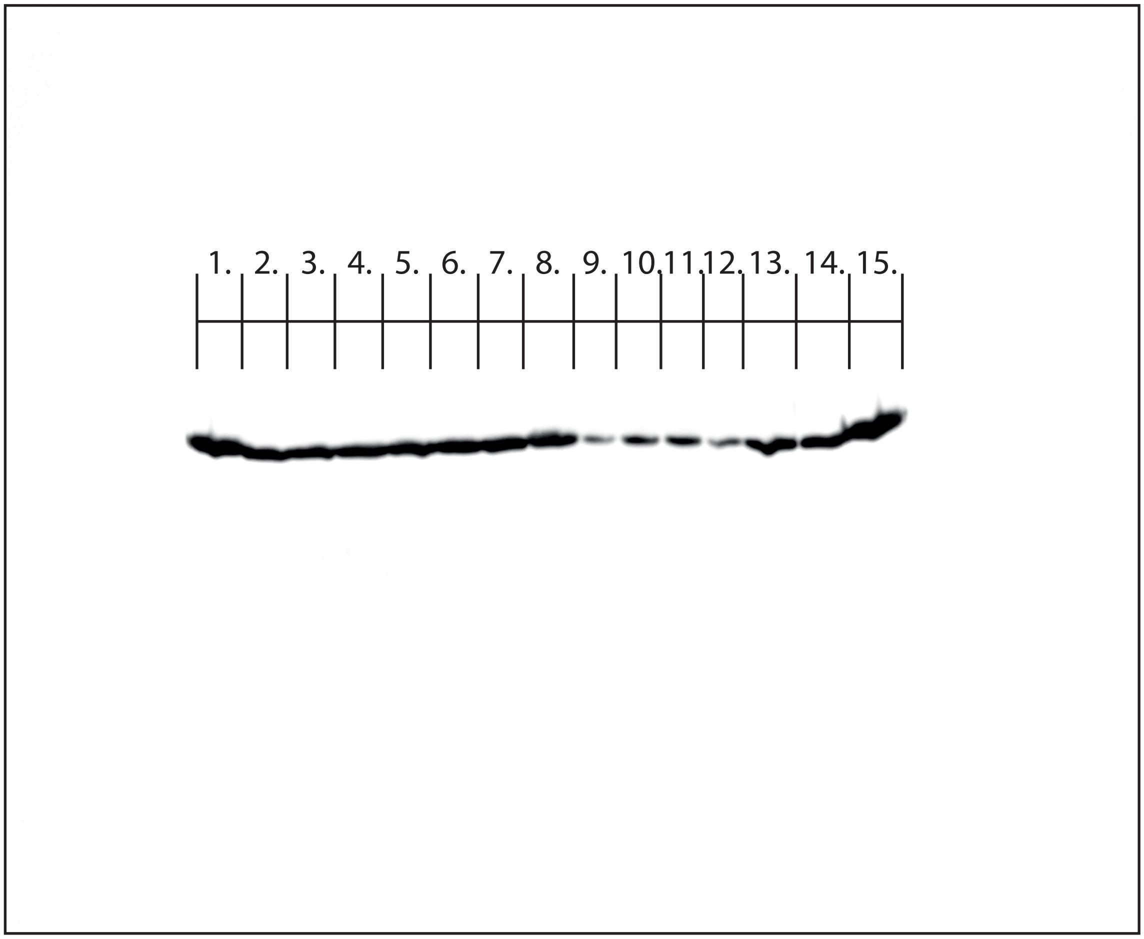

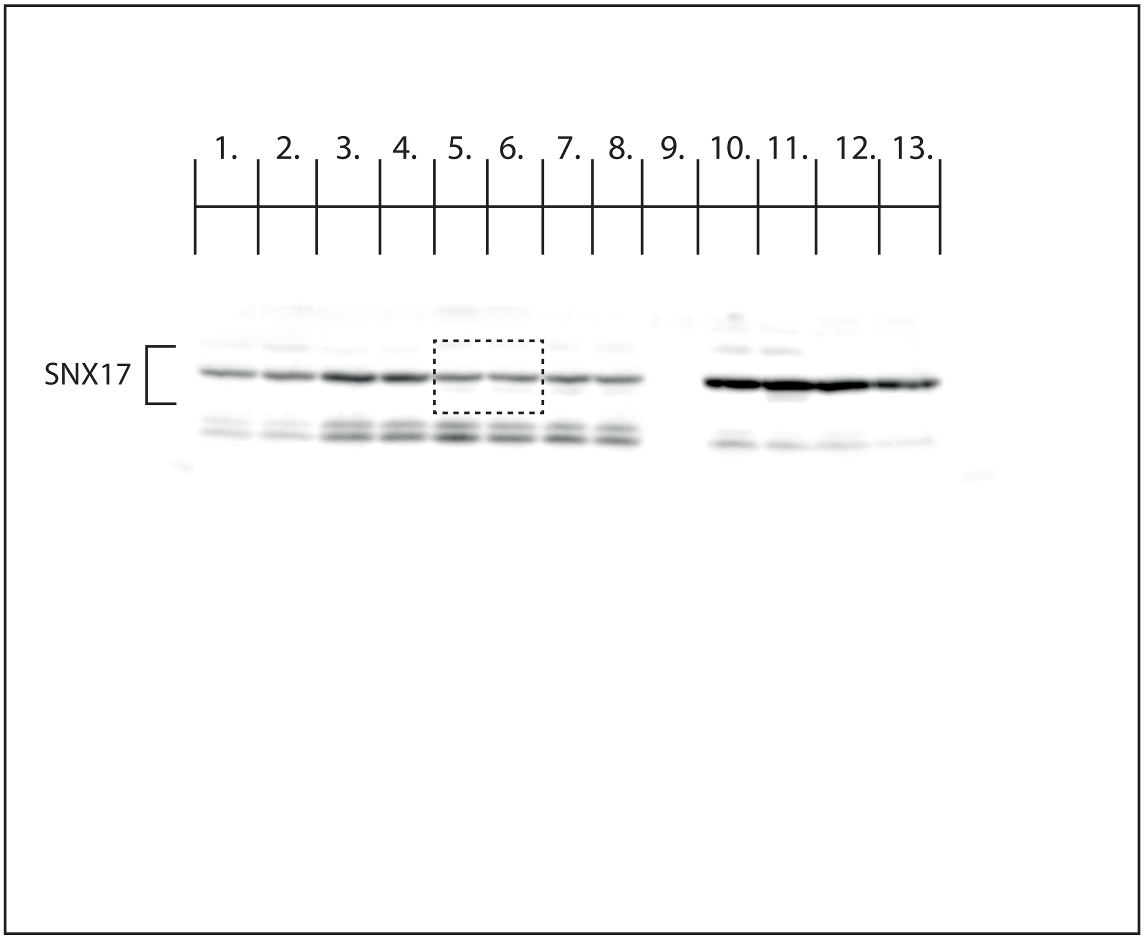


SNX17 (upper) and actin (lower)
i3 neurons, figure 5E,F

1.
2.
3.
4.
5. I3 neurons d14 wt showed in figure 5
6. neurons d14 OCRL KO showed in figure 5E
7.
8.
9. MW St
10.
11.
12.
13.

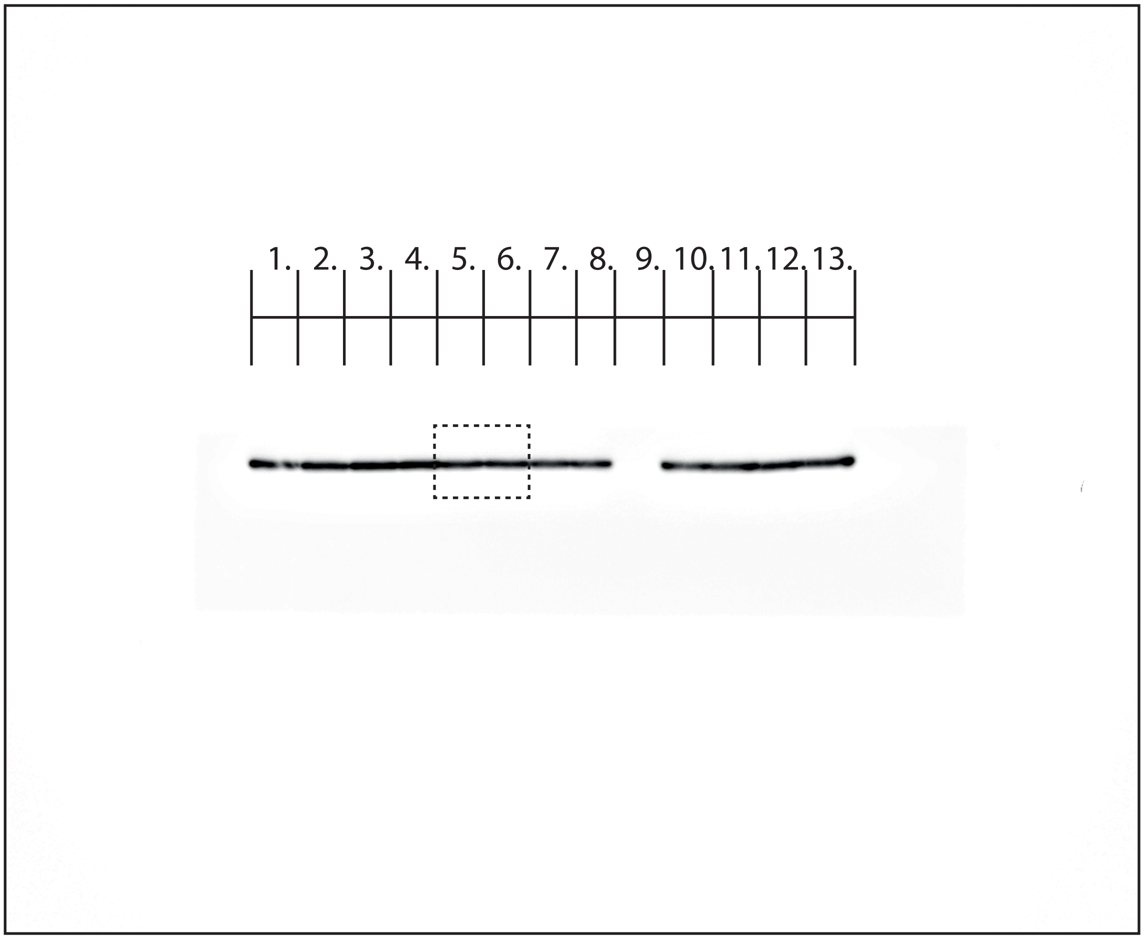

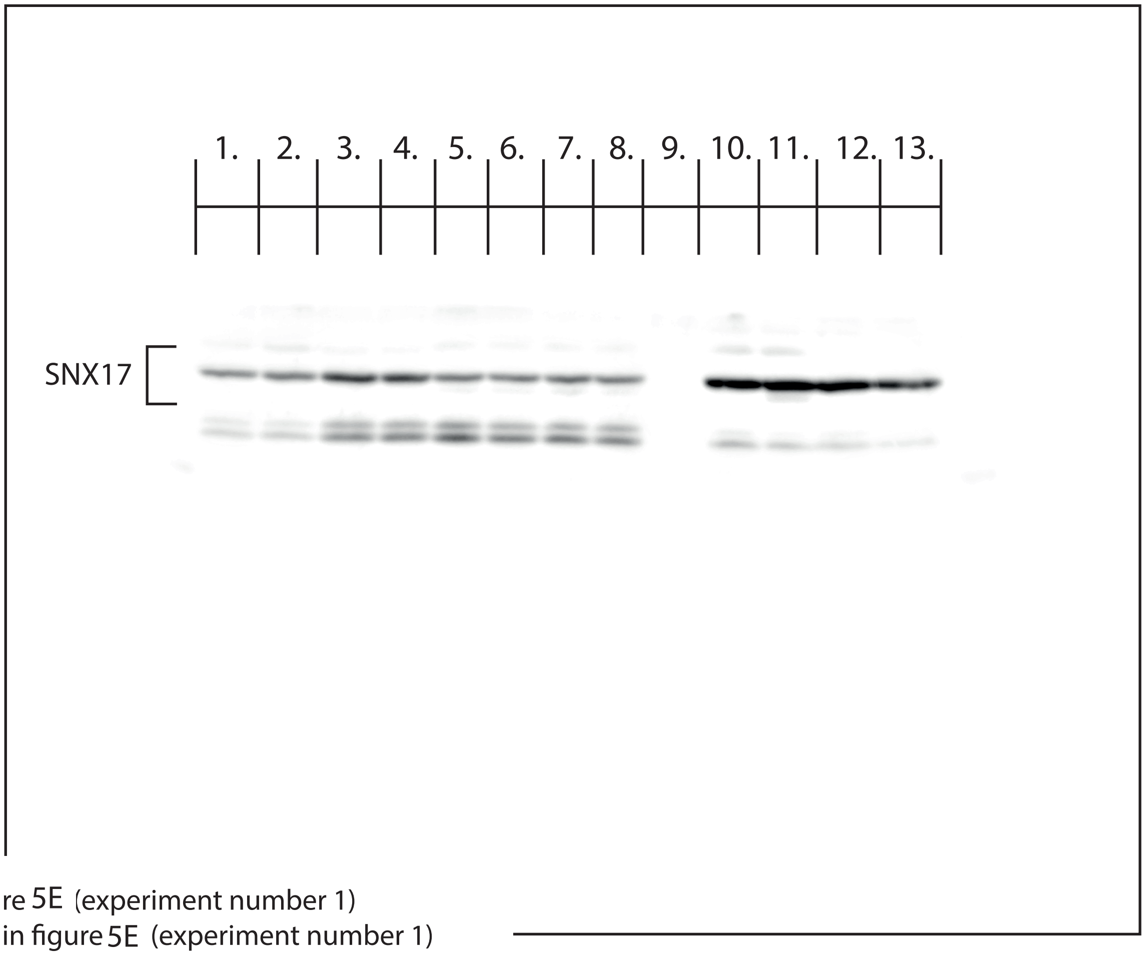


SNX17 (upper) and actin (lower) i3 neurons, figure 5E

1. I3 neurons d14 wt analized in figure 5E (experiment number 1)
2. I3 neurons d14 OCRL KO analized in figure 5E (experiment number 1)
3. I3 neurons d14 wt analized in figure 5E (experiment number 2)
4. I3 neurons d14 OCRL KO analized in figure 5E (experiment number 2)
5. I3 neurons d14 wt analized in figure 5E (experiment number 3)
6. I3 neurons d14 OCRL KO analized in figure 5E (experiment number 3)
7.
8.
9. MW St
10.
11.
12.
13.

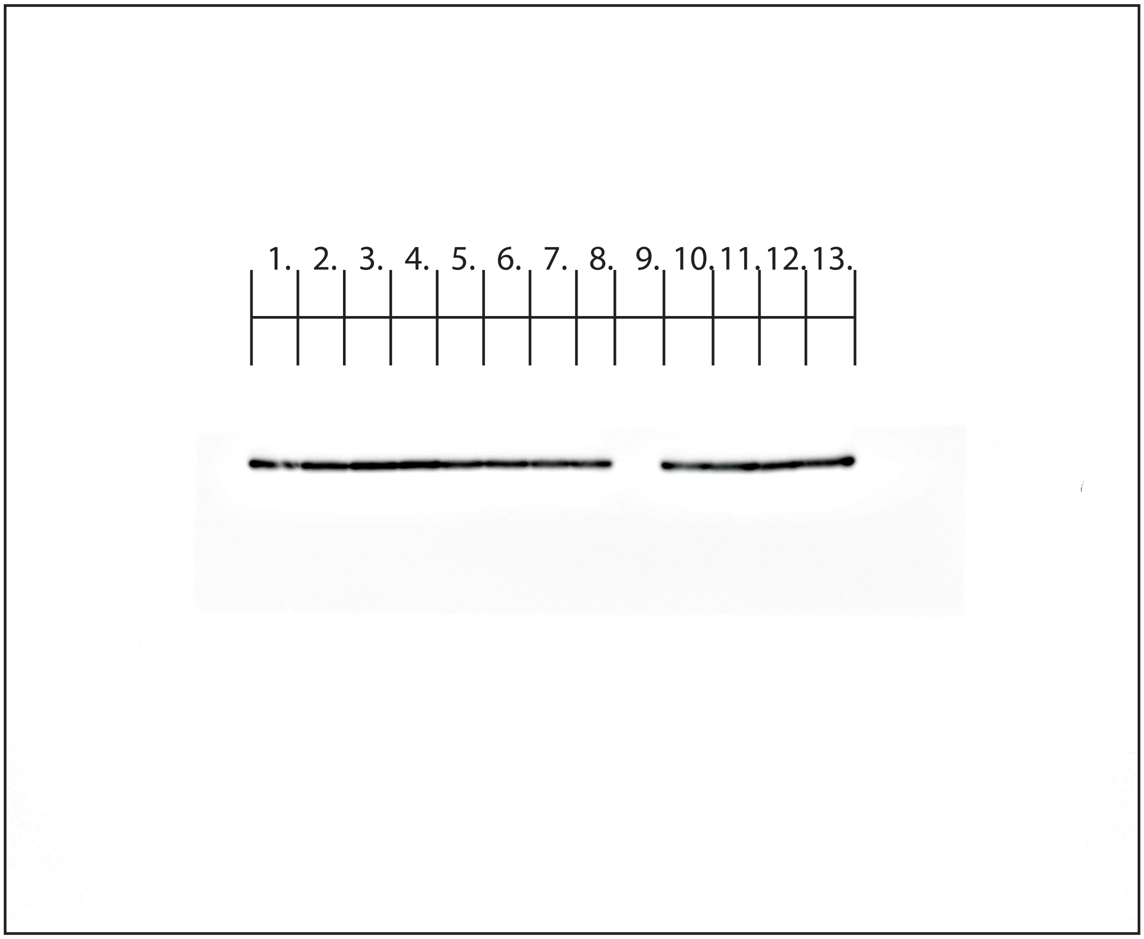

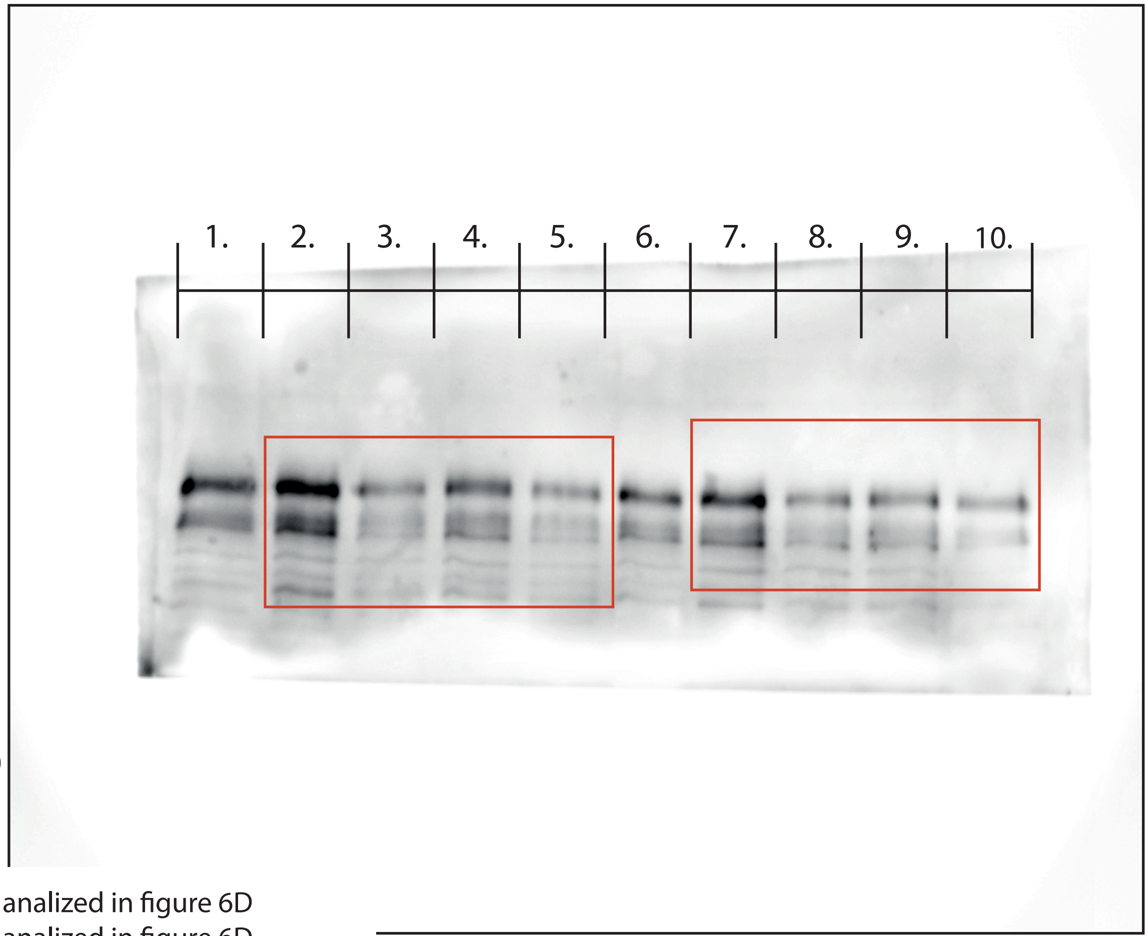


ApoER2 (upper) and tubulin (lower)
i3 neurons +/- CHX, figure 6D

1. MW St + irrelevant sample
2. I3neurons day 21, wt 0 h CHX-N2 analized in figure 6D
3. I3neurons day 21, wt 4 h CHX-N2 analized in figure 6D
4. I3neurons day 21, wt 8 h CHX-N2 analized in figure 6D
5. I3neurons day 21, wt 16 h CHX-N2 analized in figure 6D
6.
7. I3neurons day 21, KO 0 h CHX-N2 analized in figure 6D
8. I3neurons day 21, KO 4 h CHX-N2 analized in figure 6D
9. I3neurons day 21, KO 8 h CHX-N2 analized in figure 6D
10. I3neurons day 21, KO 16 h CHX-N2 analized in figure 6D

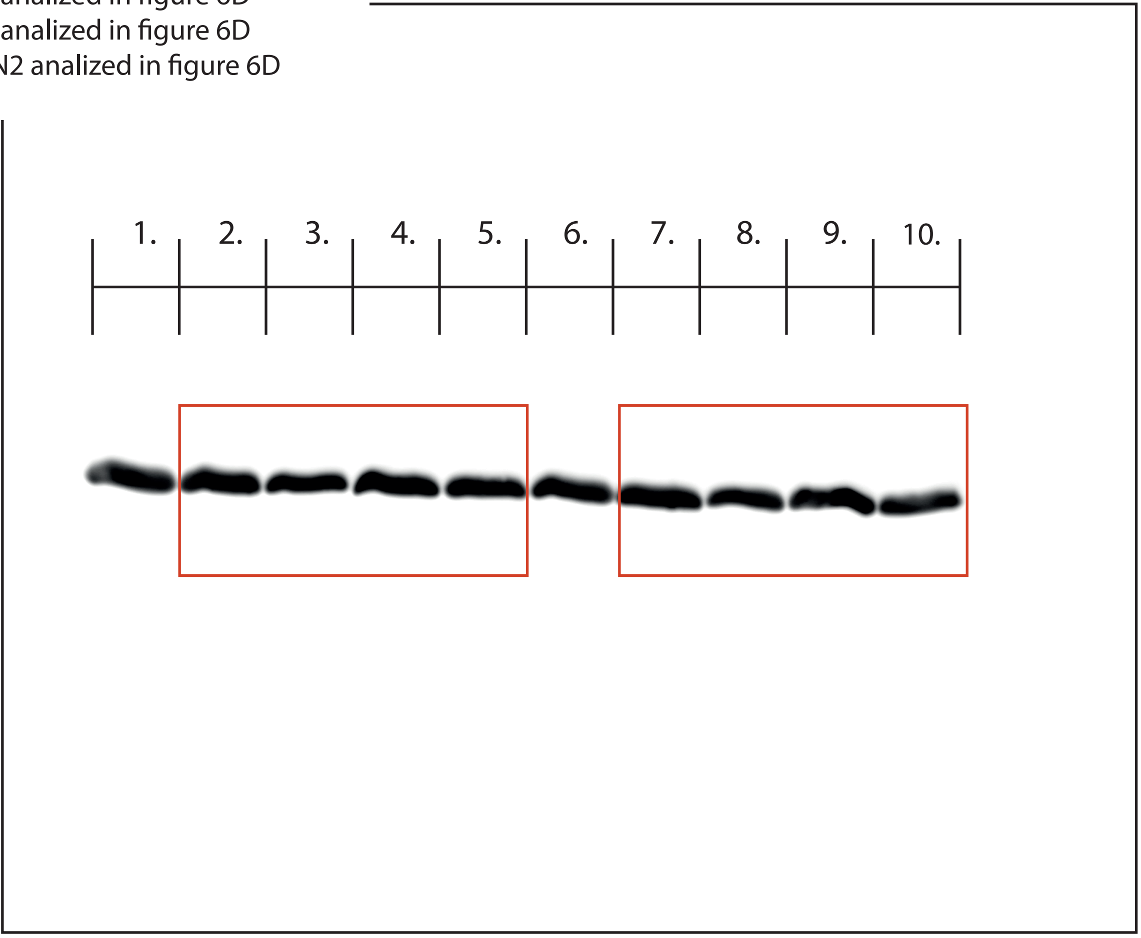

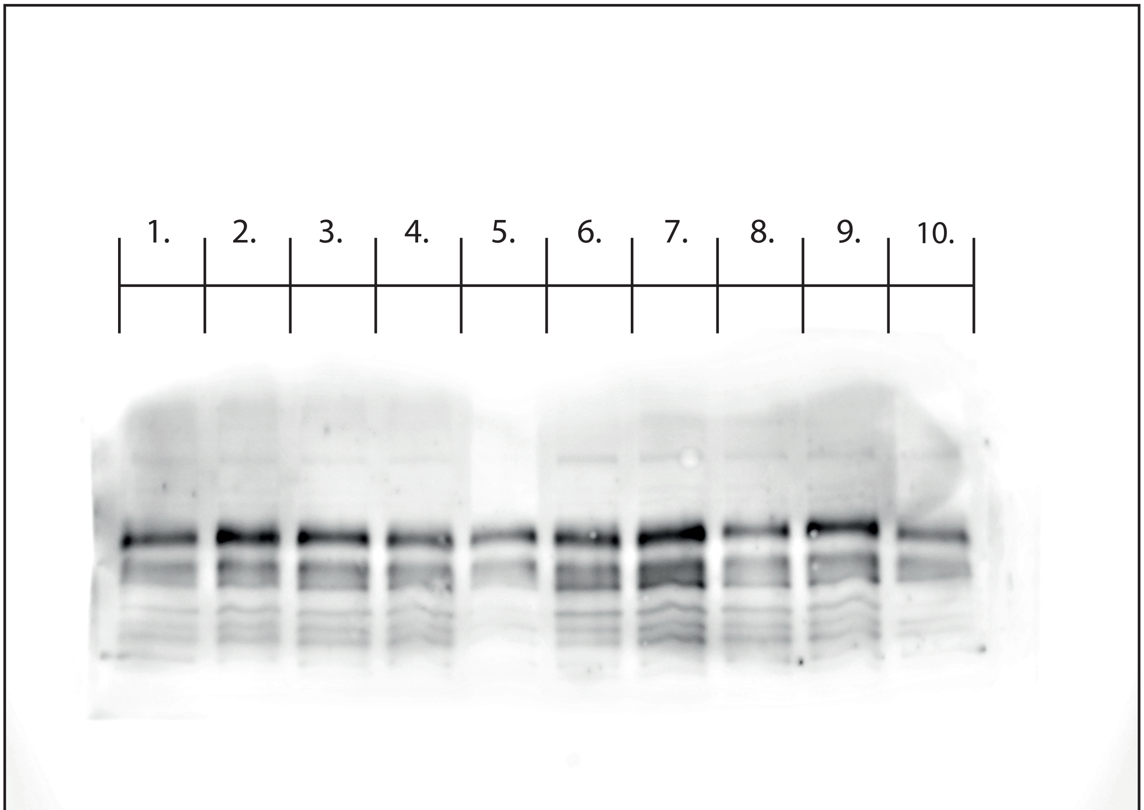


ApoER2 (upper) and tubulin (lower)
i3 neurons +/- CHX, figure 6D

1. MW St + irrelevant sample
2. I3neurons day 21, wt 0 h CHX-N1 analized in figure 6D
3. I3neurons day 21, wt 4 h CHX-N1 analized in figure 6D
4. I3neurons day 21, wt 8 h CHX-N1 analized in figure 6D
5. I3neurons day 21, wt 16 h CHX-N1 analized in figure 6D
6.
7. I3neurons day 21, KO 0 h CHX-N1 analized in figure 6D
8. I3neurons day 21, KO 4 h CHX-N1 analized in figure 6D
9. I3neurons day 21, KO 8 h CHX-N1 analized in figure 6D
10. I3neurons day 21, KO 16 h CHX-N1 analized in figure 6D

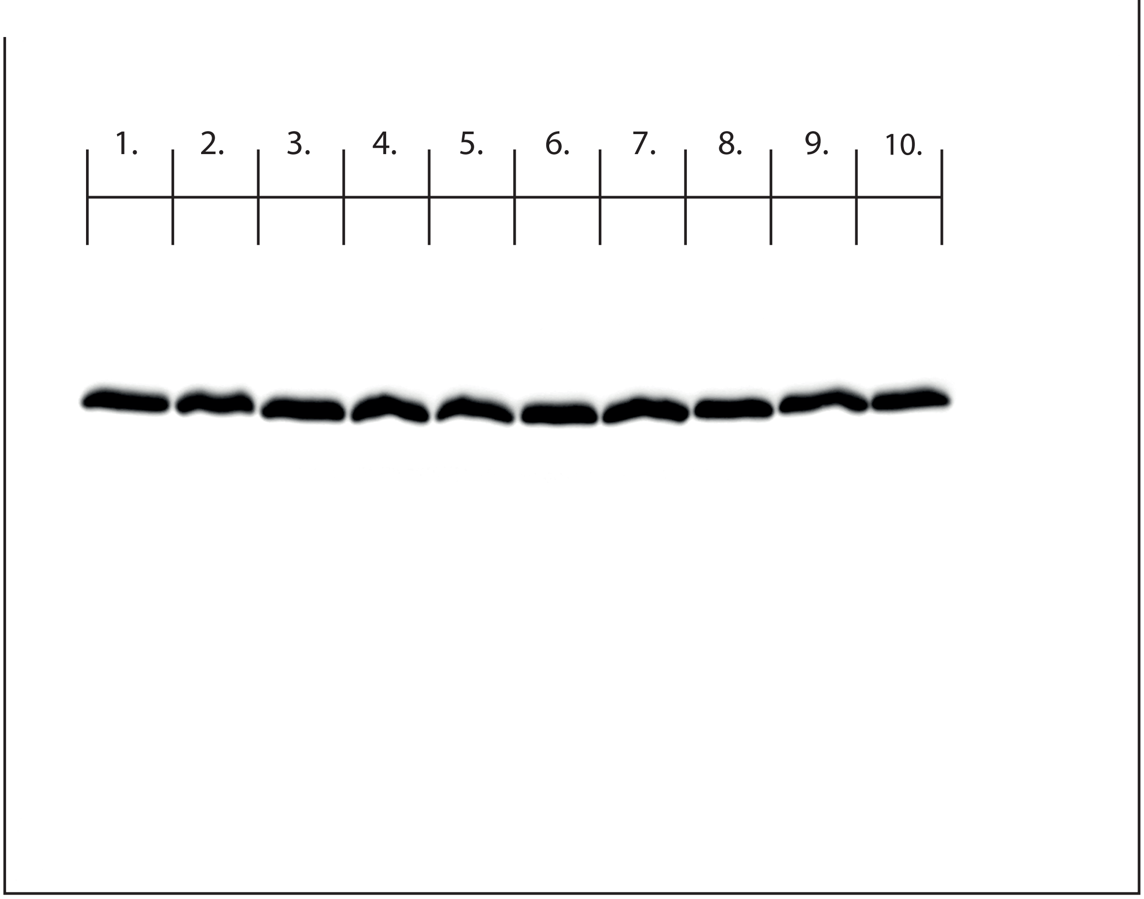

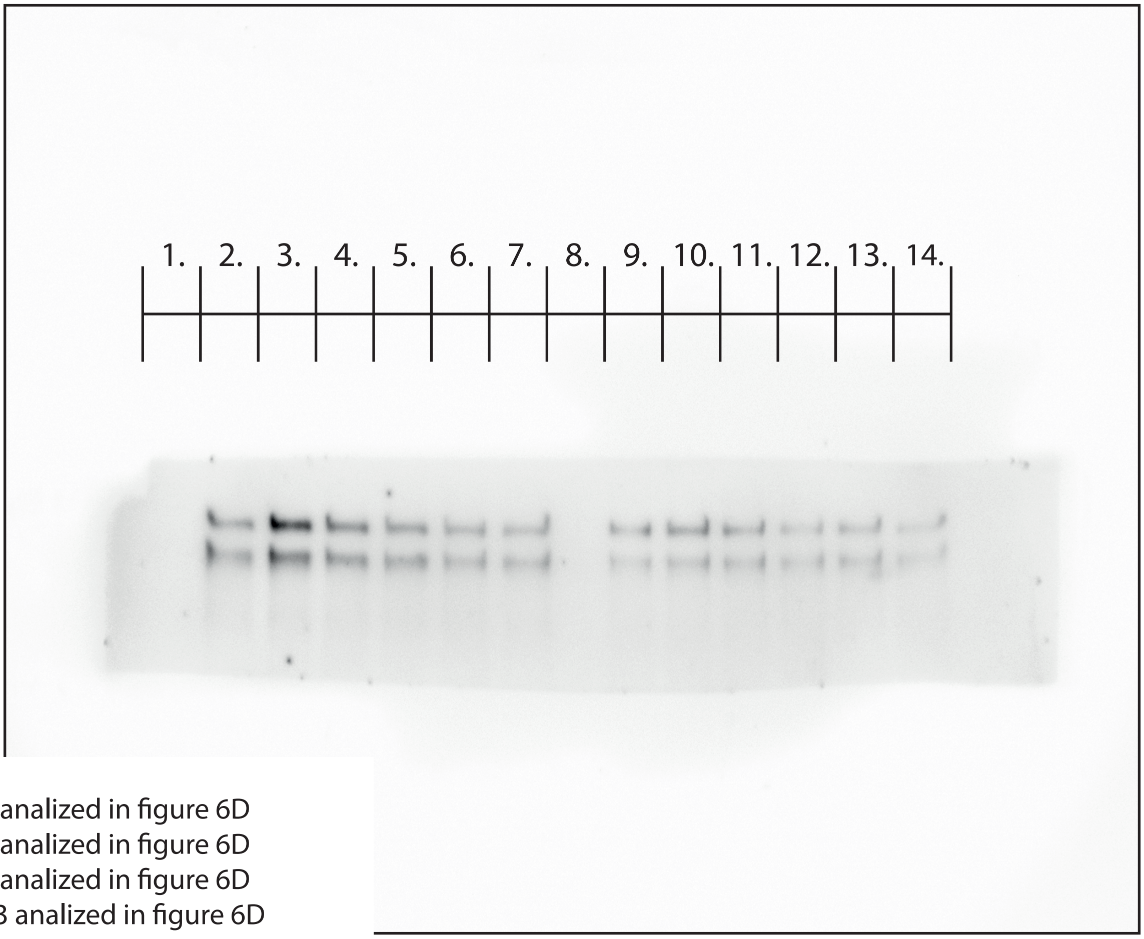


ApoER2 (upper) and tubulin (lower)
i3 neurons +/- CHX, figure 6D

1. MW St
2.
3. I3neurons day 21, wt 0 h CHX-N3 analized in figure 6D
4. I3neurons day 21, wt 4 h CHX-N3 analized in figure 6D
5. I3neurons day 21, wt 8 h CHX-N3 analized in figure 6D
6. I3neurons day 21, wt 16 h CHX-N3 analized in figure 6D
7.
8. MW St
9.
10. I3neurons day 21, KO 0 h CHX-N3 analized in figure 6D
11. I3neurons day 21, KO 4 h CHX-N3 analized in figure 6D
12. I3neurons day 21, KO 8 h CHX-N3 analized in figure 6D
13. I3neurons day 21, KO 16 h CHX-N3 analized in figure 6D
14.

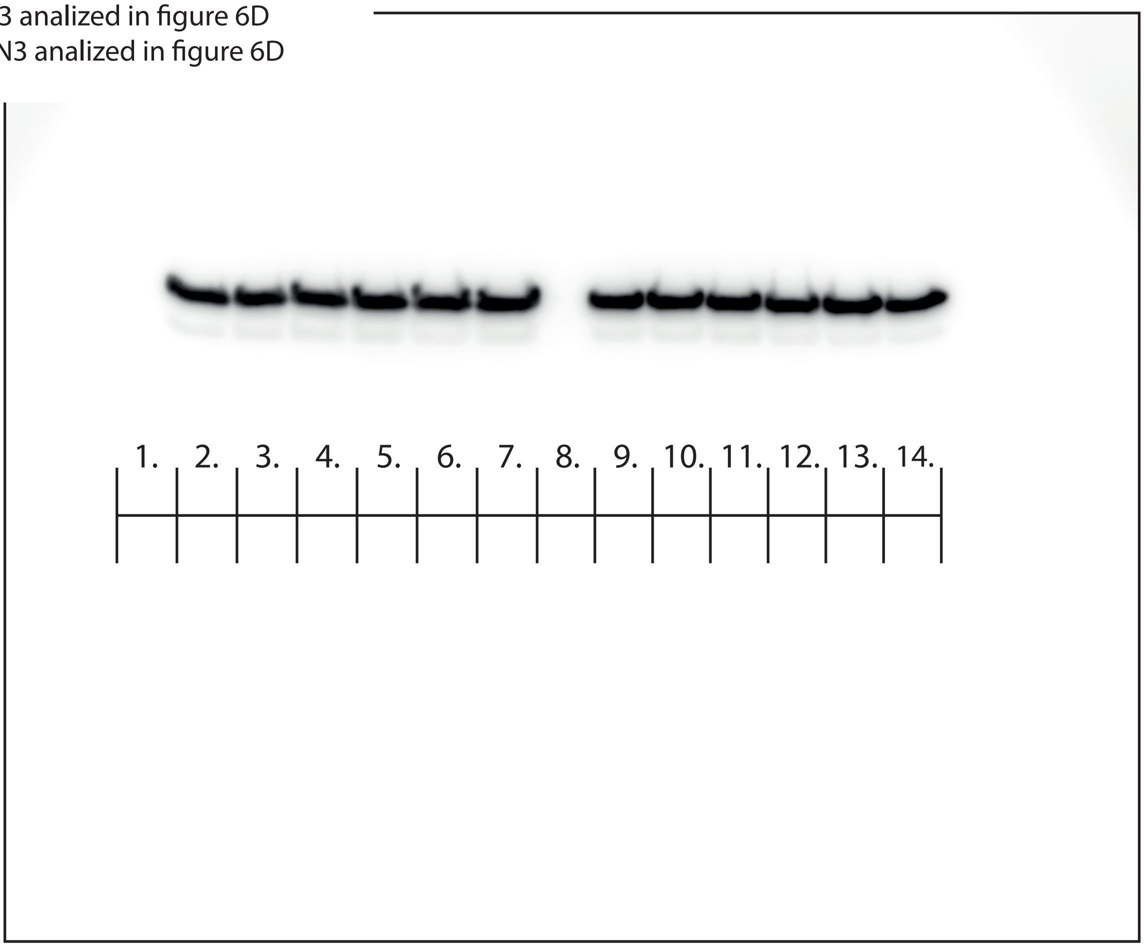

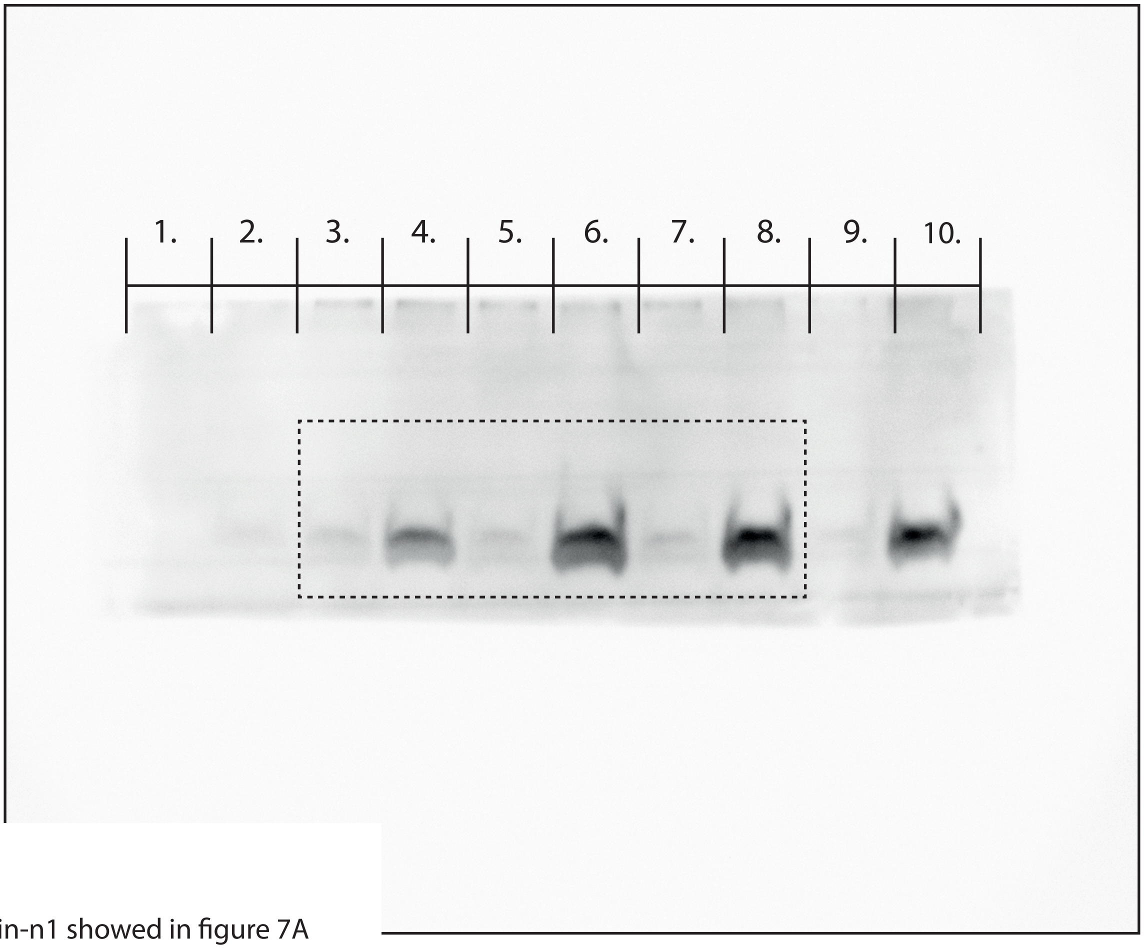


pAKT (upper) and total AKT(lower)
i3 neurons Mock or Reelin treated
figure 7A

1. MW St
2.
3. I3neurons day 21, wt mock 10 min-n1 showed in figure 7A
4. I3neurons day 21, wt reelin 10 min-n1 showed in figure 7A
5. I3neurons day 21, wt mock 20 min-n1 showed in figure 7A
6. I3neurons day 21, wt reelin 20 min-n1 showed in figure 7A
7. I3neurons day 21, wt mock 40 min-n1 showed in figure 7A
8. I3neurons day 21, wt reelin 40 min-n1 showed in figure 7A
9.
10.

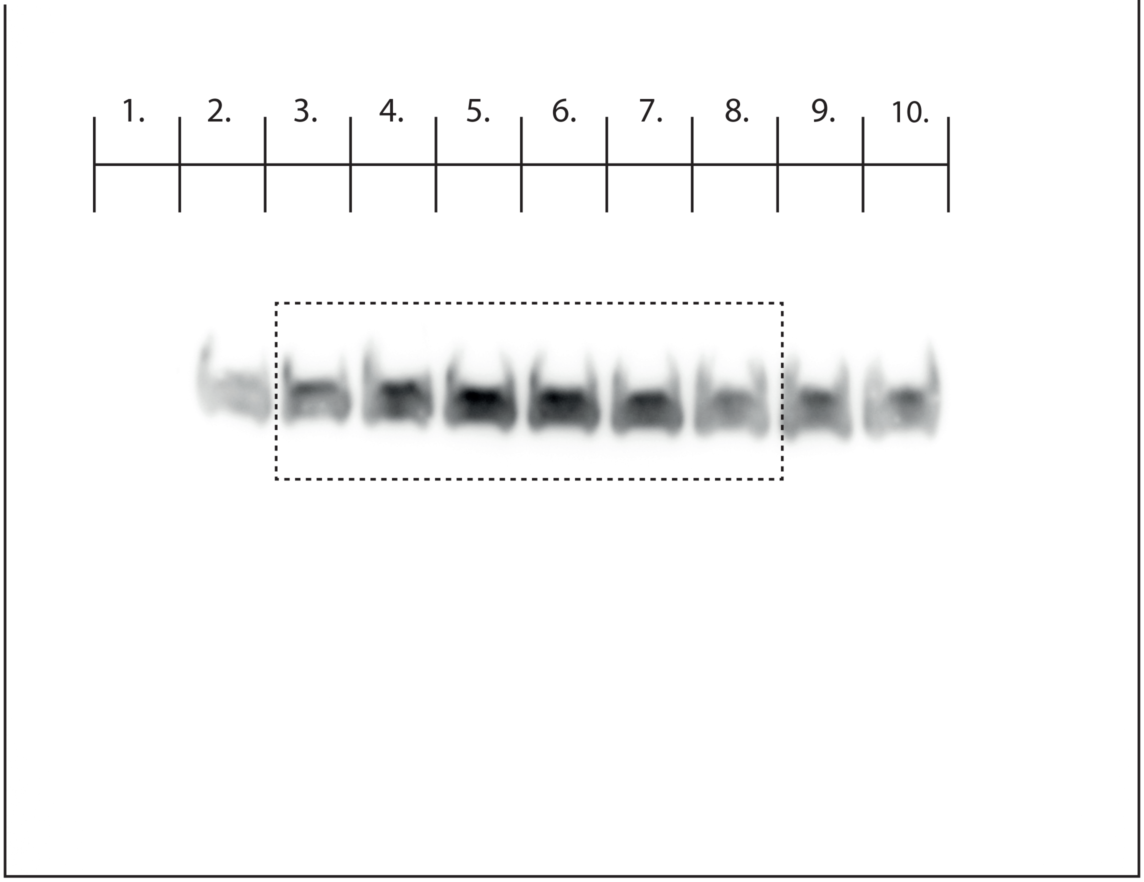

pAKT (upper) and total AKT(lower)
i3 neurons Mock or Reelin treated
figure 7A

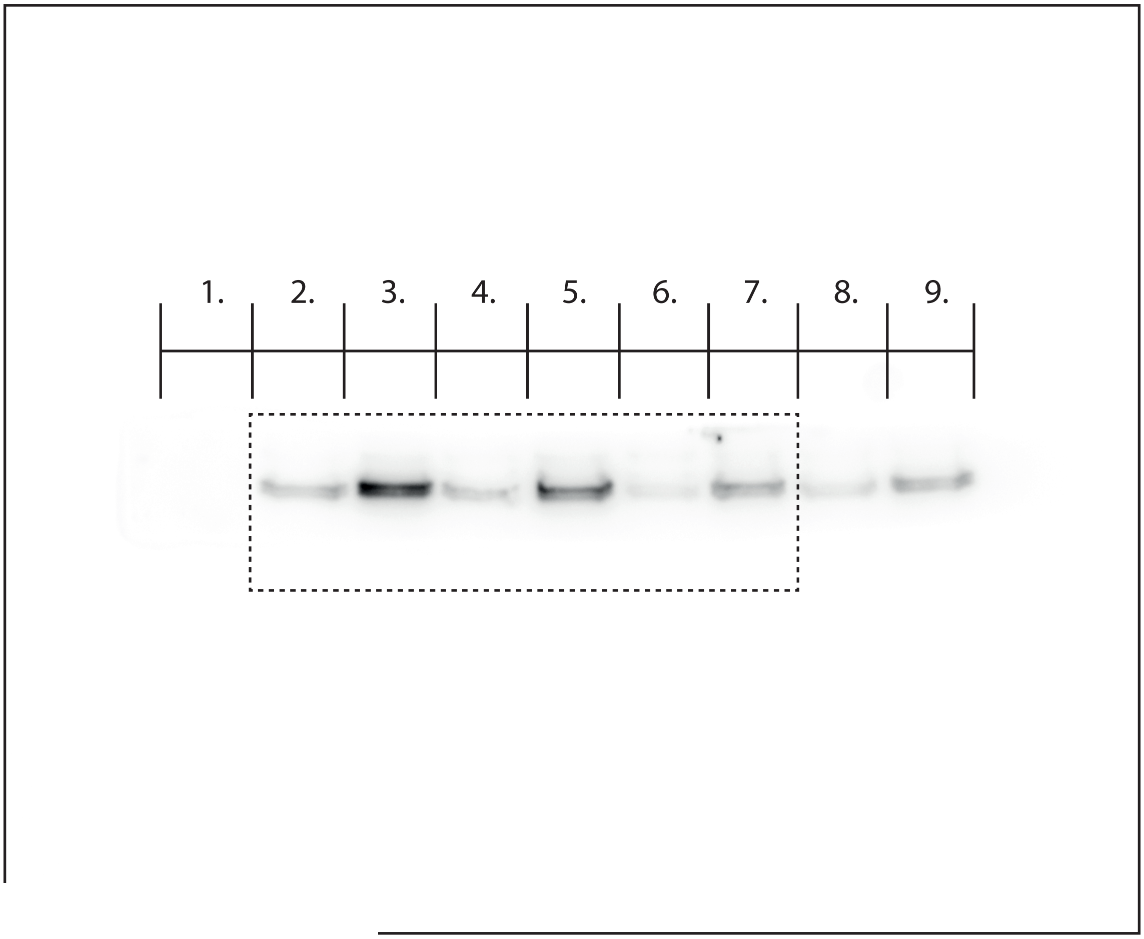


1. MW St
2. I3neurons day 21, KO mock 10 min-n3 showed in figure 7A
3. I3neurons day 21, KO reelin 10 min-n3 showed in figure 7A
4. I3neurons day 21, KO mock 20 min-n3 showed in figure 7A
5. I3neurons day 21, KO reelin 20 min-n3 showed in figure 7A
6. I3neurons day 21, KO mock 40 min-n3 showed in figure 7A
7. I3neurons day 21, KO reelin 40 min-n3 showed in figure 7A
8.
9.

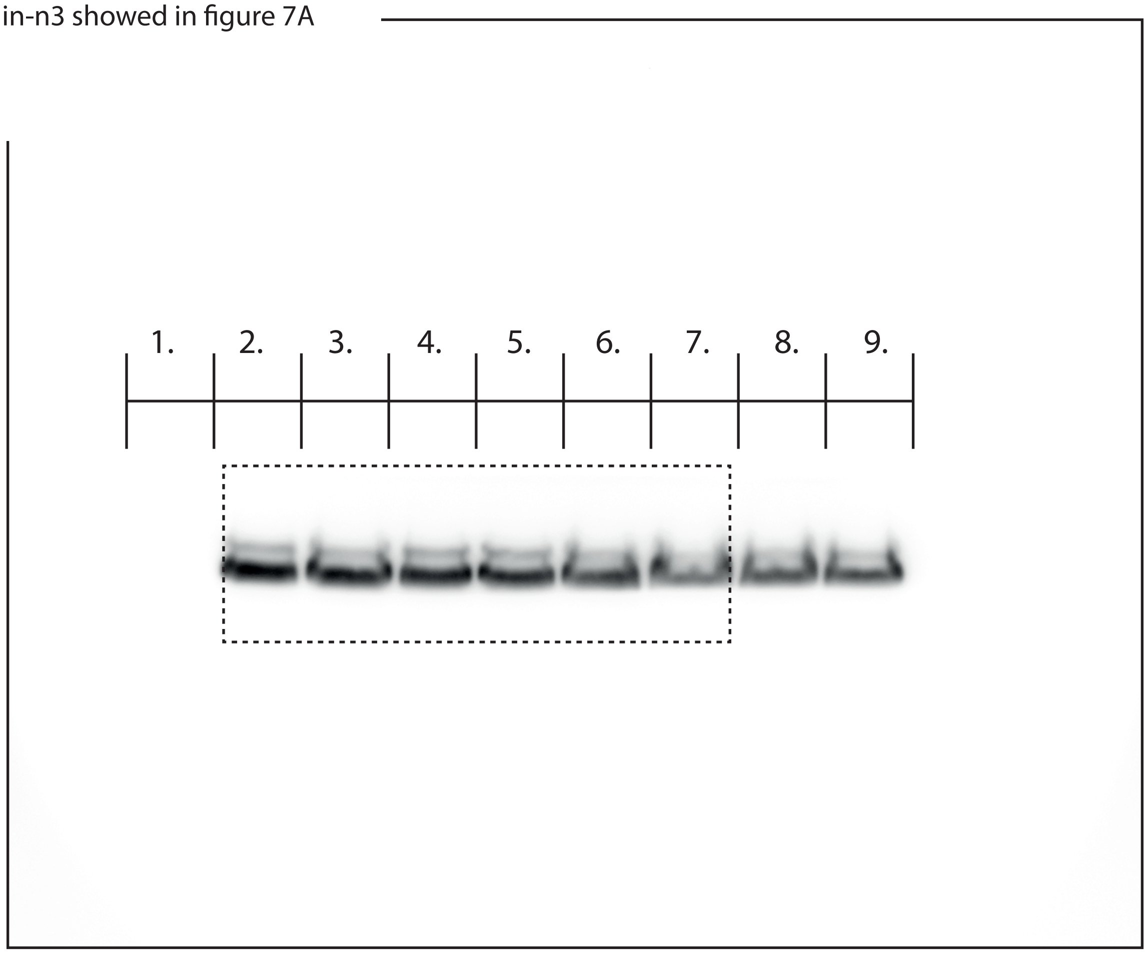

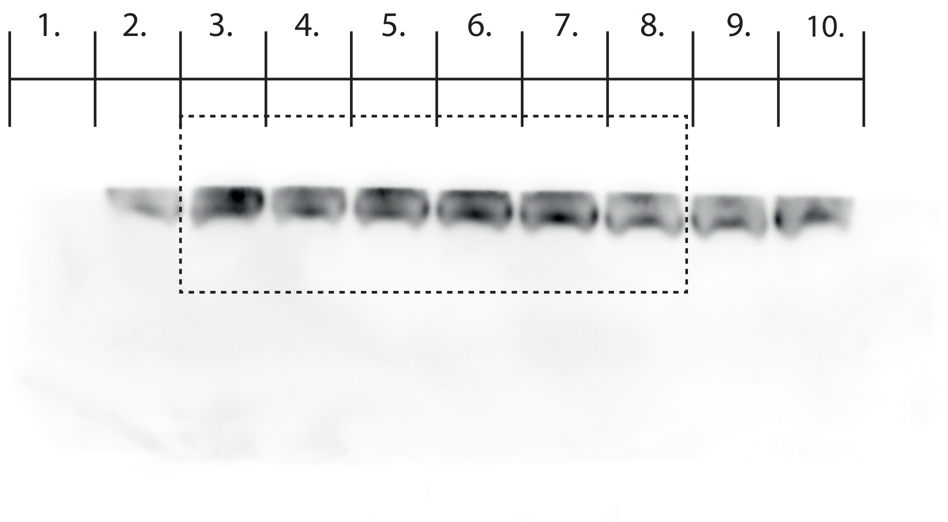


actin
i3 neurons Mock or Reelin treated
figure 7A

1. MW St
2.
3. I3neurons day 21, wt mock 10 min-n1 showed in figure 7A
4. I3neurons day 21, wt reelin 10 min-n1 showed in figure 7A
5. I3neurons day 21, wt mock 20 min-n1 showed in figure 7A
6. I3neurons day 21, wt reelin 20 min-n1 showed in figure 7A
7. I3neurons day 21, wt mock 40 min-n1 showed in figure 7A
8. I3neurons day 21, wt reelin 40 min-n1 showed in figure 7A
9.
10.

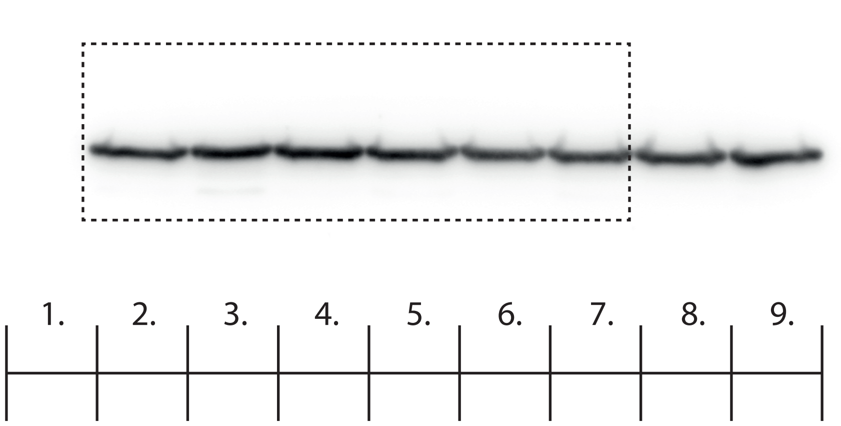


actin
i3 neurons Mock or Reelin treated
figure 7A

1. MW St
2. I3neurons day 21, KO mock 10 min-n3 showed in figure 7A
3. I3neurons day 21, KO reelin 10 min-n3 showed in figure 7A
4. I3neurons day 21, KO mock 20 min-n3 showed in figure 7A
5. I3neurons day 21, KO reelin 20 min-n3 showed in figure 7A
6. I3neurons day 21, KO mock 40 min-n3 showed in figure 7A
7. I3neurons day 21, KO reelin 40 min-n3 showed in figure 7A
8.
9.

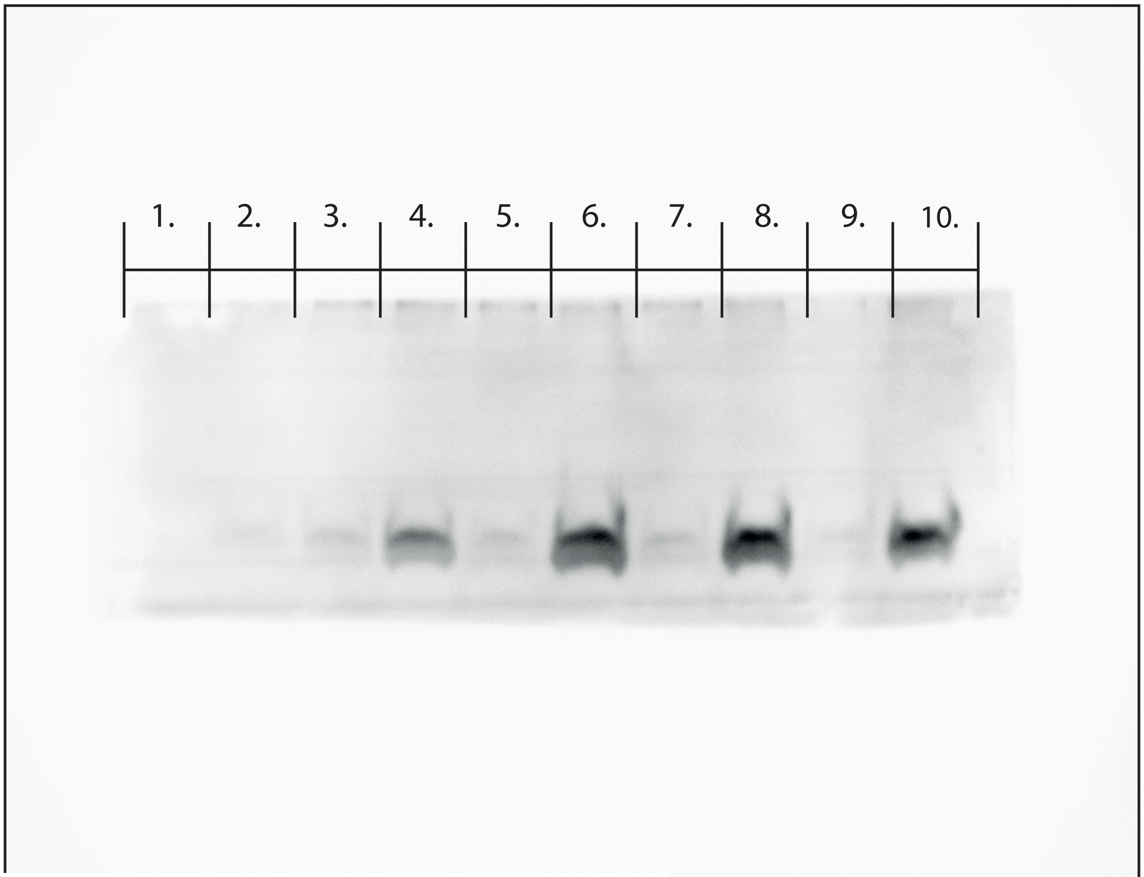


pAKT (upper) and total AKT (lower)
i3 neurons Mock or Reelin treated
figure 7B

1. MW St
2.
3. I3neurons day 21, wt mock 10 min-n1 (experiment number 1) analyzed in figure 7B
4. I3neurons day 21, wt reelin 10 min-n1 (experiment number 1) analyzed in figure 7B
5. I3neurons day 21, wt mock 20 min-n1 (experiment number 1) analyzed in figure 7B
6. I3neurons day 21, wt reelin 20 min-n1 (experiment number 1) analyzed in figure 7B
7. I3neurons day 21, wt mock 40 min-n1 (experiment number 1) analyzed in figure 7B
8. I3neurons day 21, wt reelin 40 min-n1 (experiment number 1) analyzed in figure 7B
9.
10.

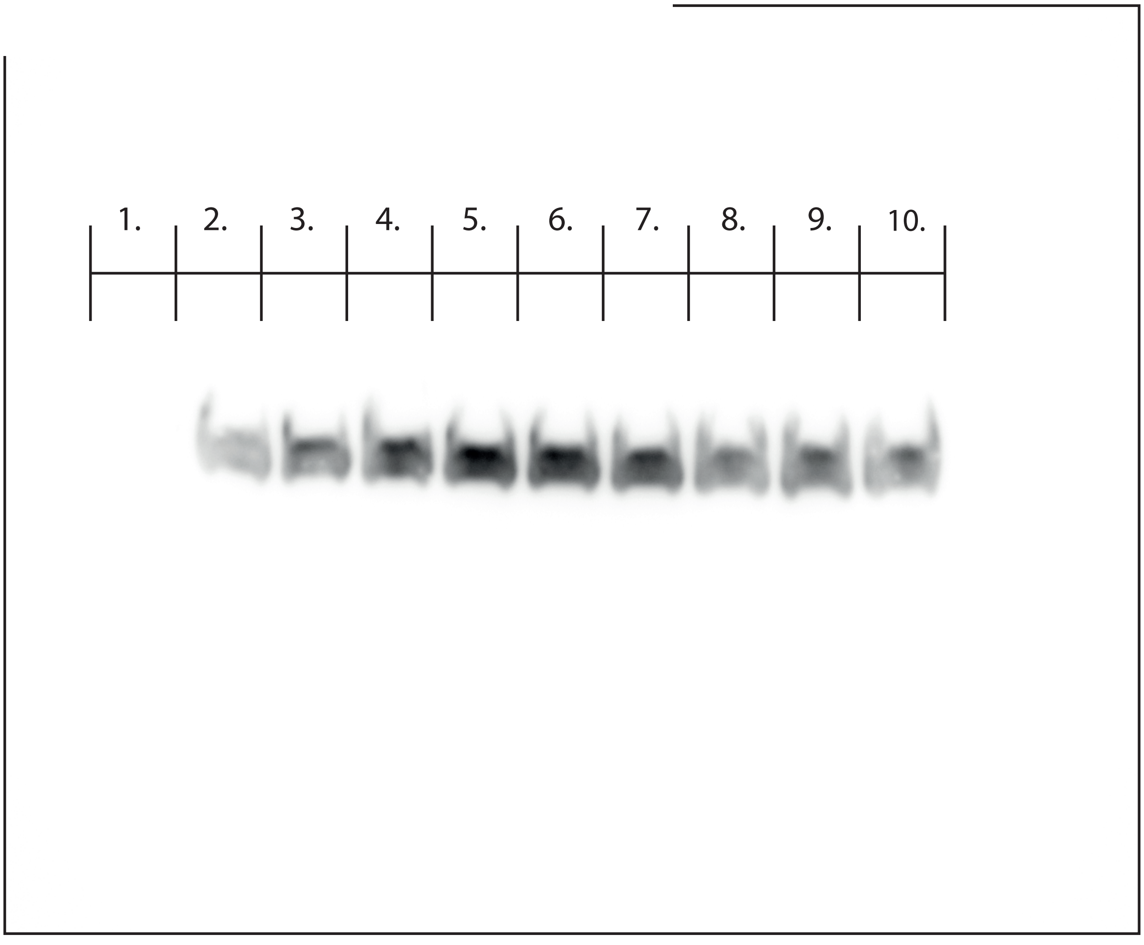

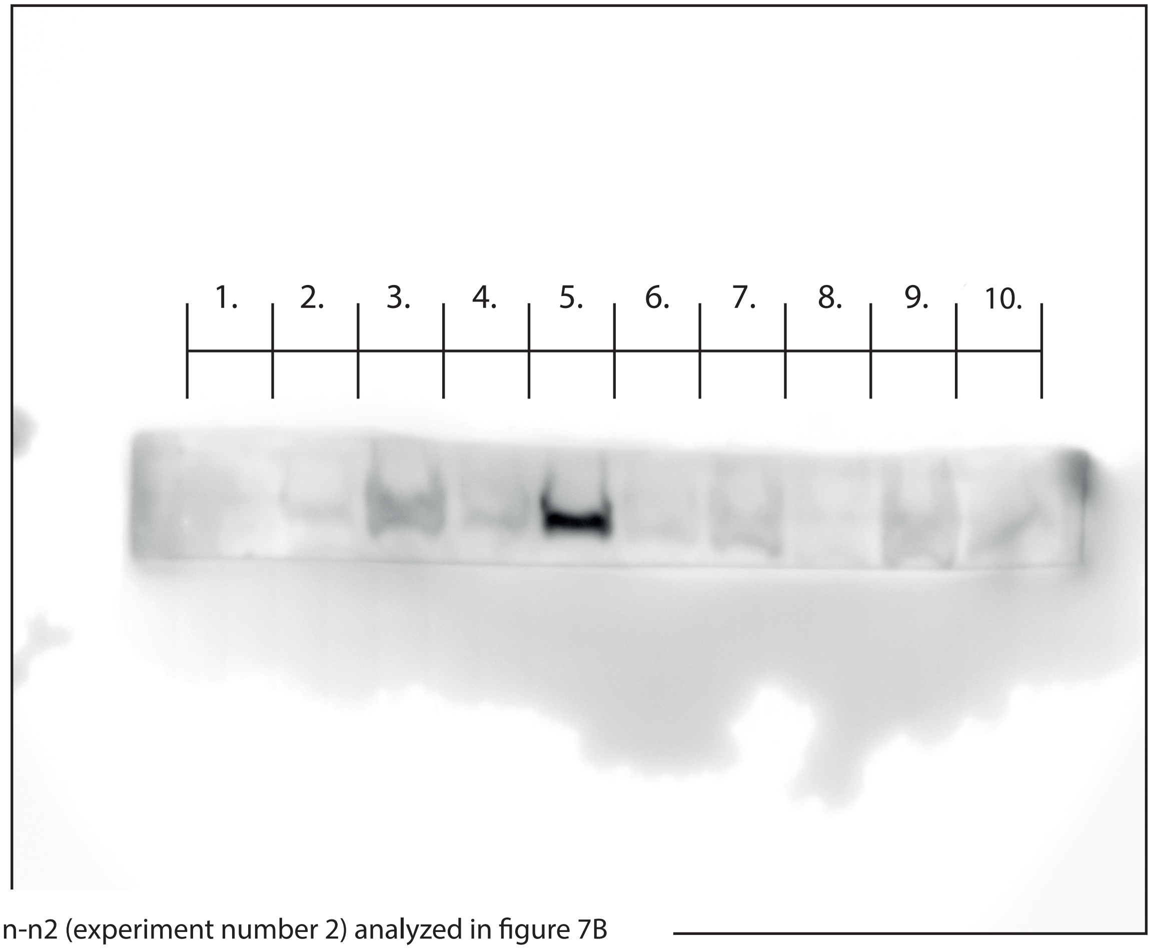


pAKT (upper) and total AKT (lower)
i3 neurons Mock or Reelin treated
figure 7B

1. MW St
2. I3neurons day 21, wt mock 10 min-n2 (experiment number 2) analyzed in figure 7B
3. I3neurons day 21, wt reelin 10 min-n2 (experiment number 2) analyzed in figure 7B
4. I3neurons day 21, wt mock 20 min-n2 (experiment number 2) analyzed in figure 7B
5. I3neurons day 21, wt reelin 20 min-n2 (experiment number 2) analyzed in figure 7B
6. I3neurons day 21, wt mock 40 min-n2 (experiment number 2) analyzed in figure 7B
7. I3neurons day 21, wt reelin 40 min-n2 (experiment number 2) analyzed in figure 7B
8.
9.
10.

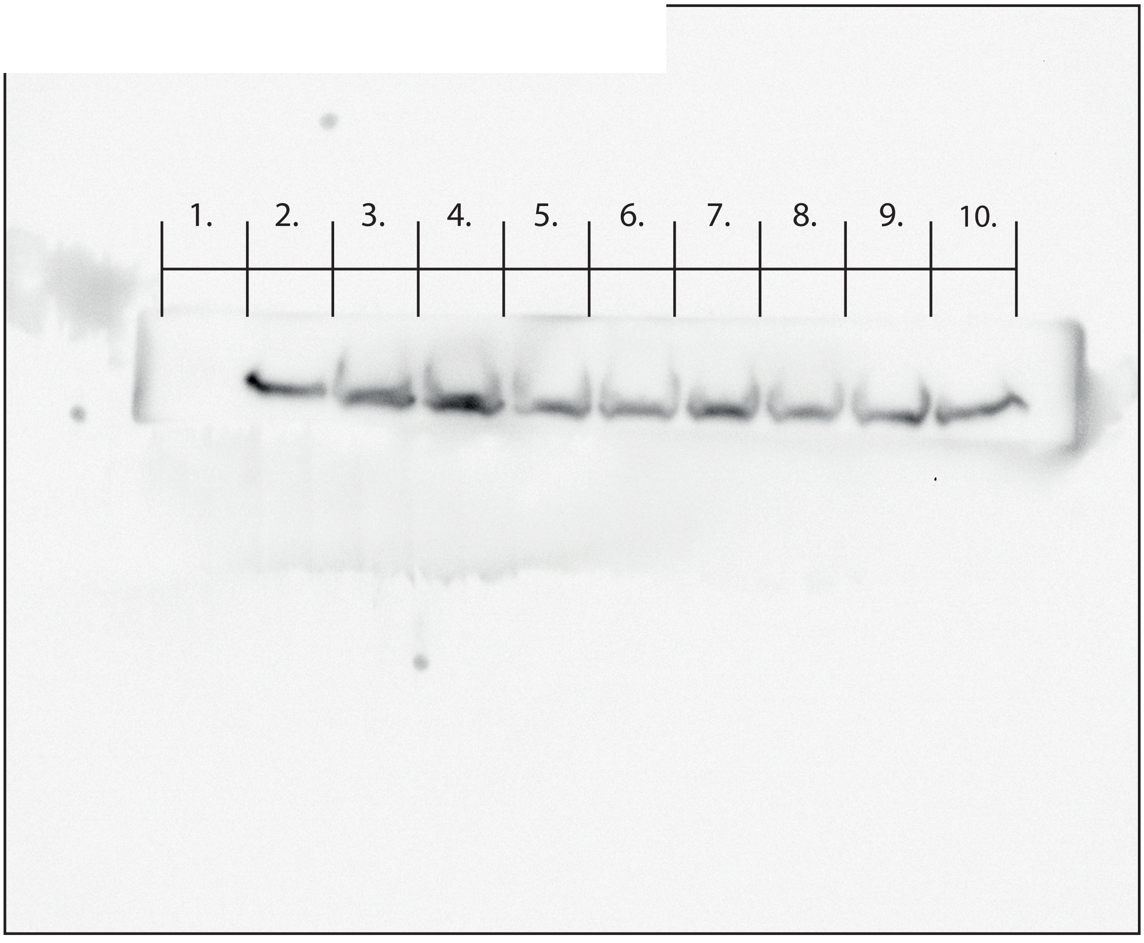

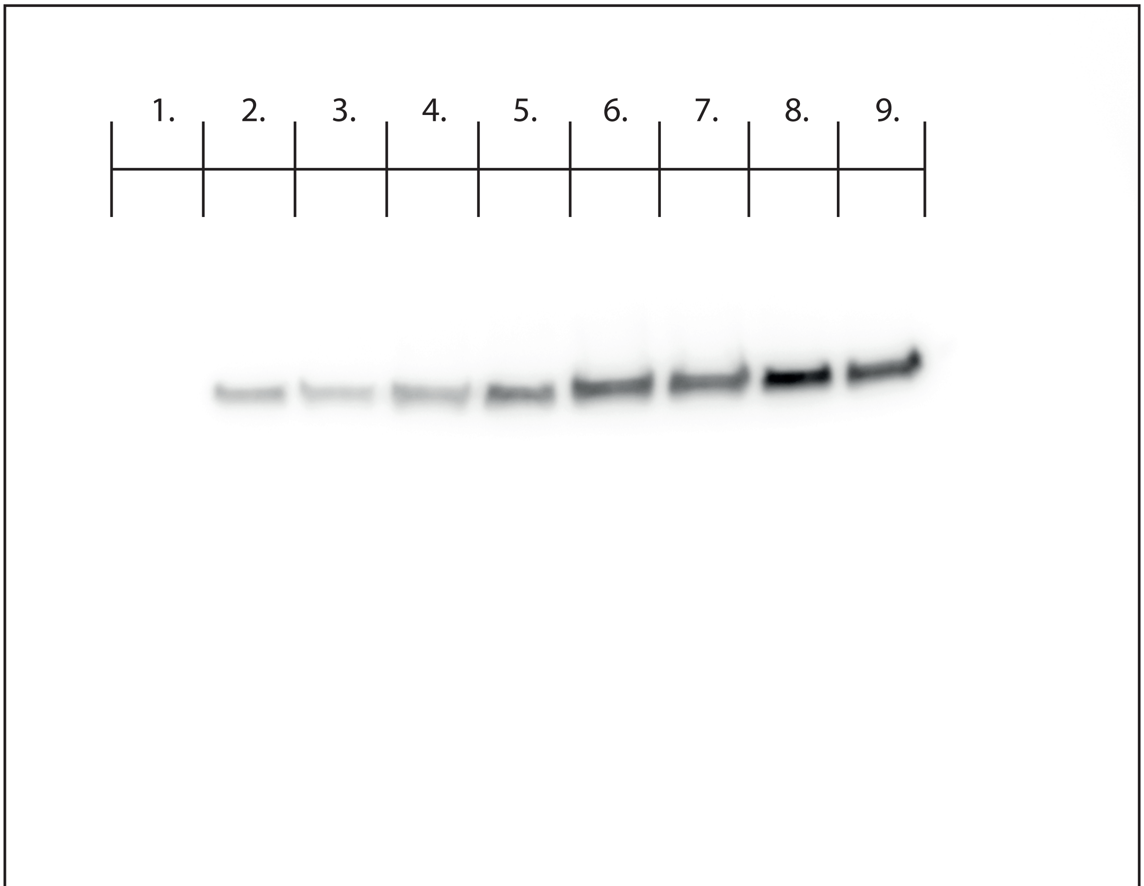


pAKT (upper) and total AKT (lower)
i3 neurons Mock or Reelin treated
figure 7B

1. MW St
2. I3neurons day 21, wt mock 10 min-n3 (experiment number 3) analyzed in figure 7B
3. I3neurons day 21, wt mock 20 min-n3 (experiment number 3) analyzed in figure 7B
4. I3neurons day 21, wt mock 40 min-n3 (experiment number 3) analyzed in figure 7B
5.
6. I3neurons day 21, wt reelin 10 min-n3 (experiment number 3) analyzed in figure 7B
7. I3neurons day 21, wt reelin 20 min-n3 (experiment number 3) analyzed in figure 7B
8. I3neurons day 21, wt reelin 40 min-n3 (experiment number 3) analyzed in figure 7B
9.

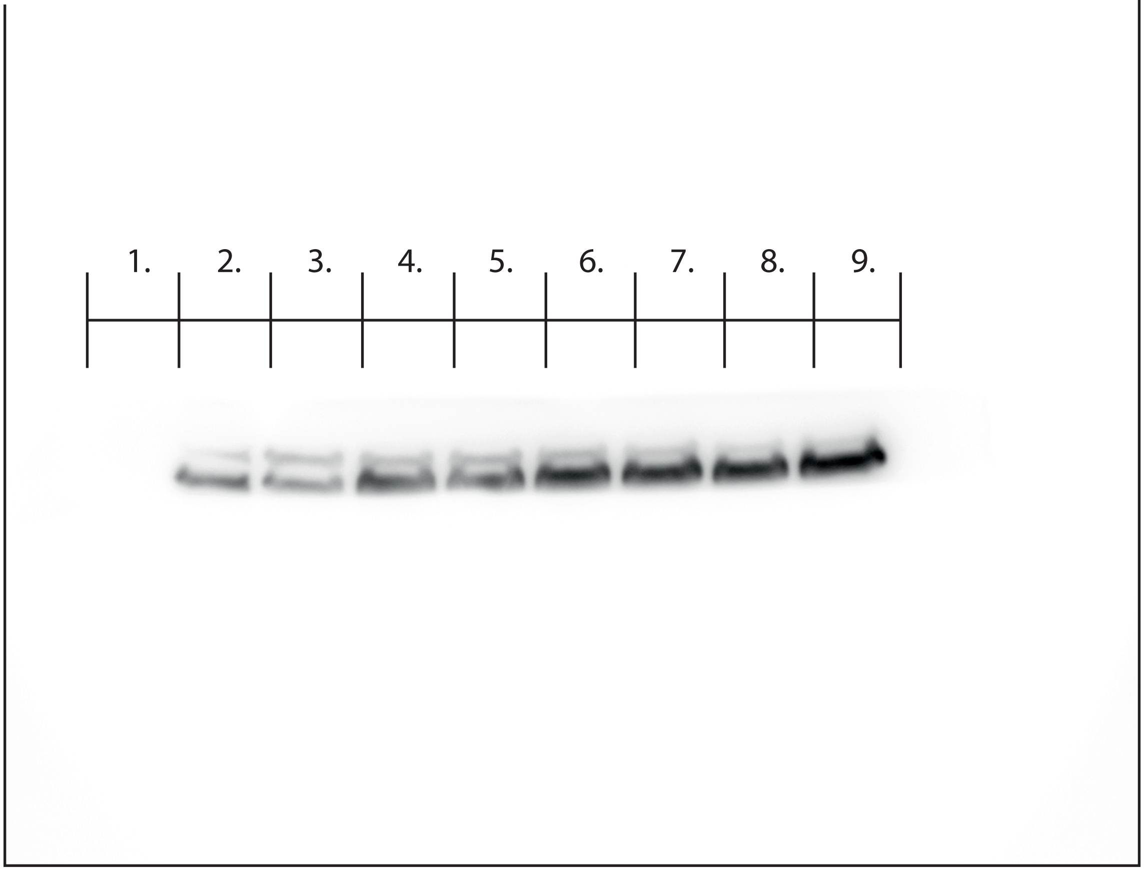

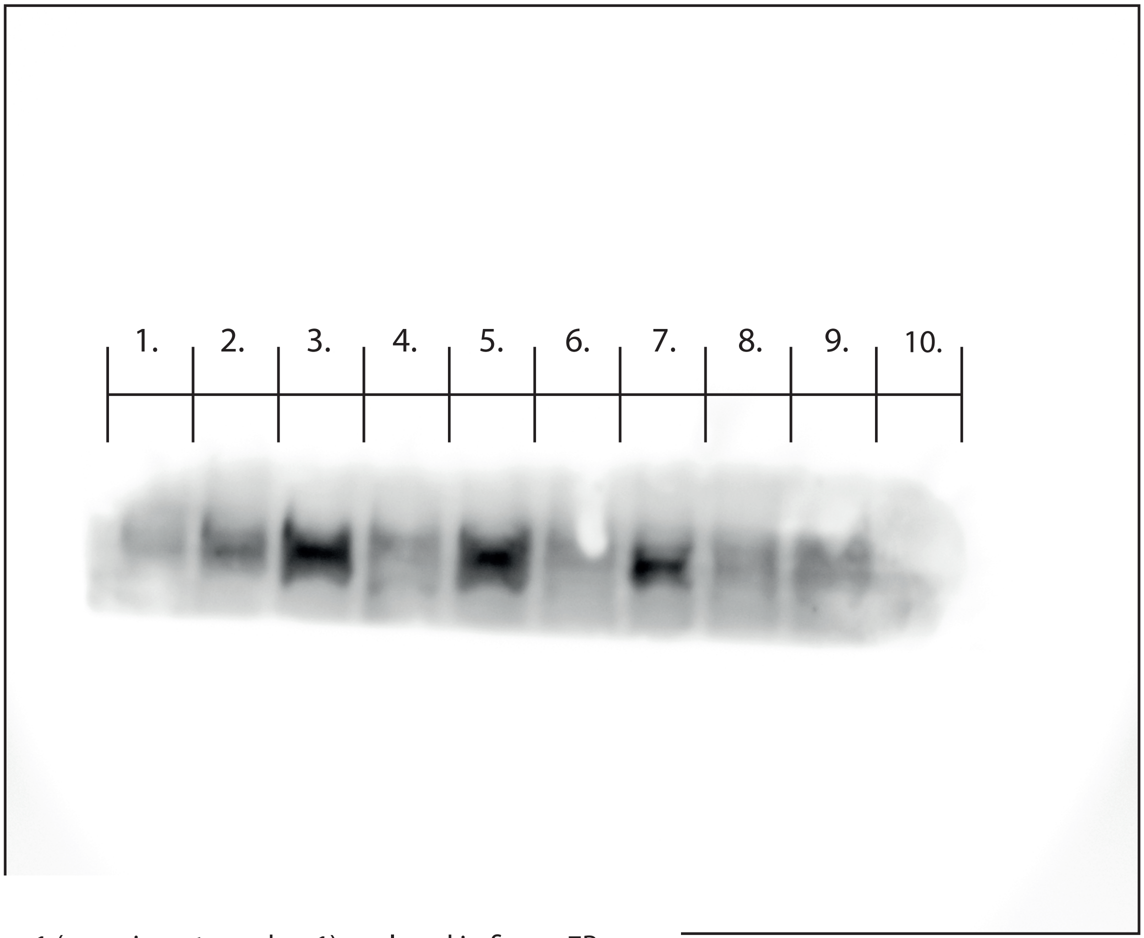


pAKT (upper) and total AKT (lower)
i3 neurons Mock or Reelin treated
figure 7B

1. MW St
2. I3neurons day 21, KO mock 10 min-n1 (experiment number 1) analyzed in figure 7B
3. I3neurons day 21, KO reelin 10 min-n1 (experiment number 1) analyzed in figure 7B
4. I3neurons day 21, KO mock 20 min-n1 (experiment number 1) analyzed in figure 7B
5. I3neurons day 21, KO reelin 20 min-n1 (experiment number 1) analyzed in figure 7B
6. I3neurons day 21, KO mock 40 min-n1 (experiment number 1) analyzed in figure 7B
7. I3neurons day 21, KO reelin 40 min-n1 (experiment number 1) analyzed in figure 7B
8.
9.
10.

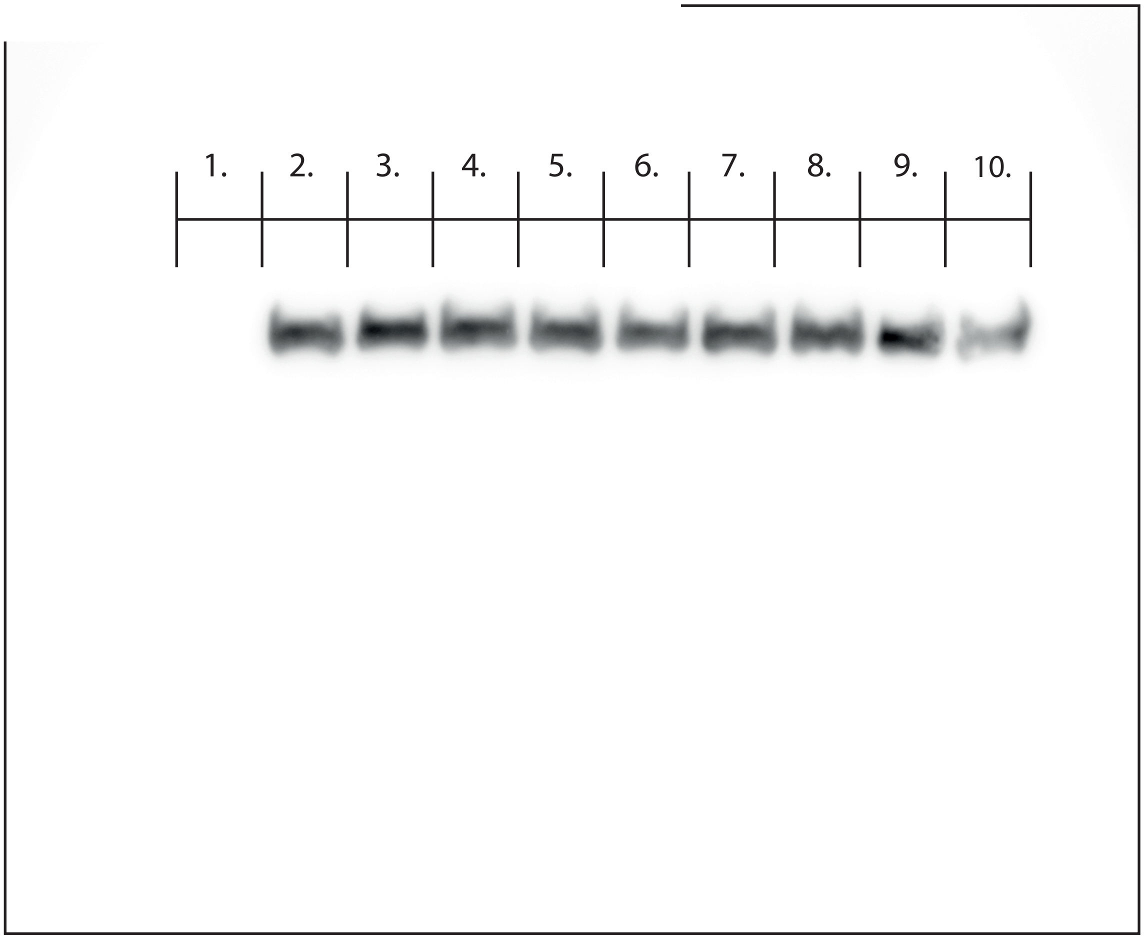

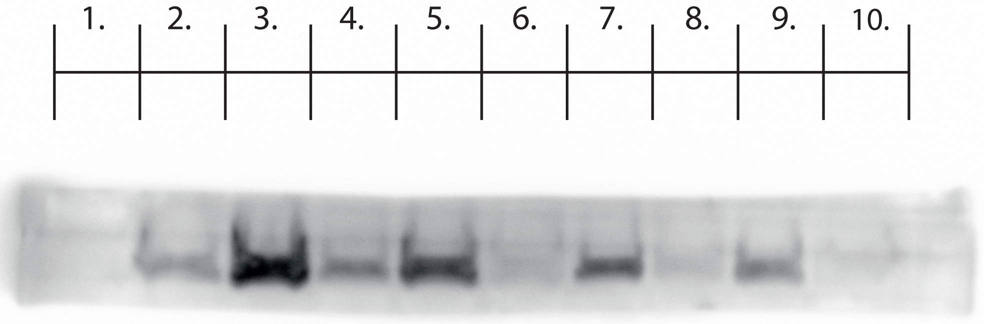


pAKT (upper) and total AKT (lower)
i3 neurons Mock or Reelin treated
figure 7B

1. MW St
2. I3neurons day 21, KO mock 10 min-n2 (experiment number 2) analyzed in figure 7B
3. I3neurons day 21, KO reelin 10 min-n2 (experiment number 2) analyzed in figure 7B
4. I3neurons day 21, KO mock 20 min-n2 (experiment number 2) analyzed in figure 7B
5. I3neurons day 21, KO reelin 20 min-n2 (experiment number 2) analyzed in figure 7B
6. I3neurons day 21, KO mock 40 min-n2 (experiment number 2) analyzed in figure 7B
7. I3neurons day 21, KO reelin 40 min-n2 (experiment number 2) analyzed in figure 7B
8.
9.
10.

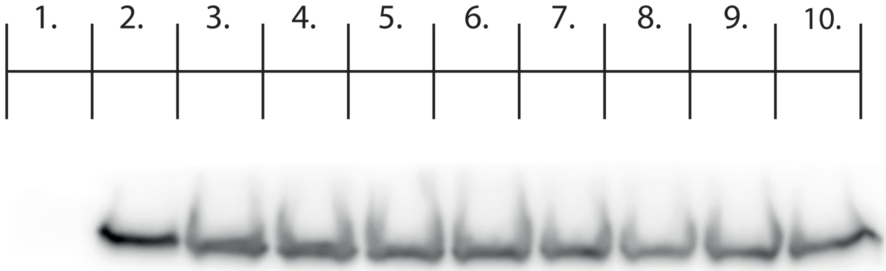

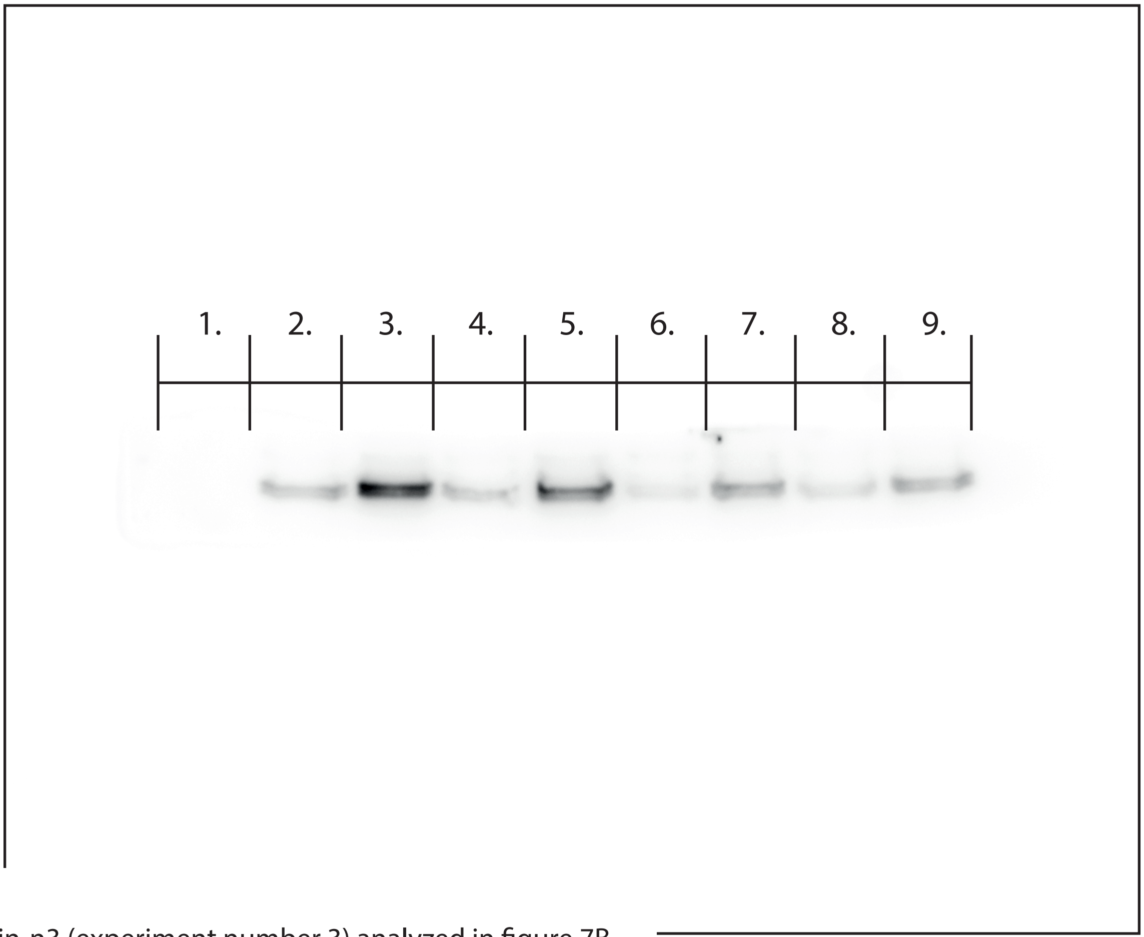


pAKT (upper) and total AKT (lower)
i3 neurons Mock or Reelin treated
figure 7B

1. MW St
2. I3neurons day 21, KO mock 10 min-n3 (experiment number 3) analyzed in figure 7B
3. I3neurons day 21, KO reelin 10 min-n3 (experiment number 3) analyzed in figure 7B
4. I3neurons day 21, KO mock 20 min-n3 (experiment number 3) analyzed in figure 7B
5. I3neurons day 21, KO reelin 20 min-n3 (experiment number 3) analyzed in figure 7B
6. I3neurons day 21, KO mock 40 min-n3 (experiment number 3) analyzed in figure 7B
7. I3neurons day 21, KO reelin 40 min-n3 (experiment number 3) analyzed in figure 7B
8.
9.

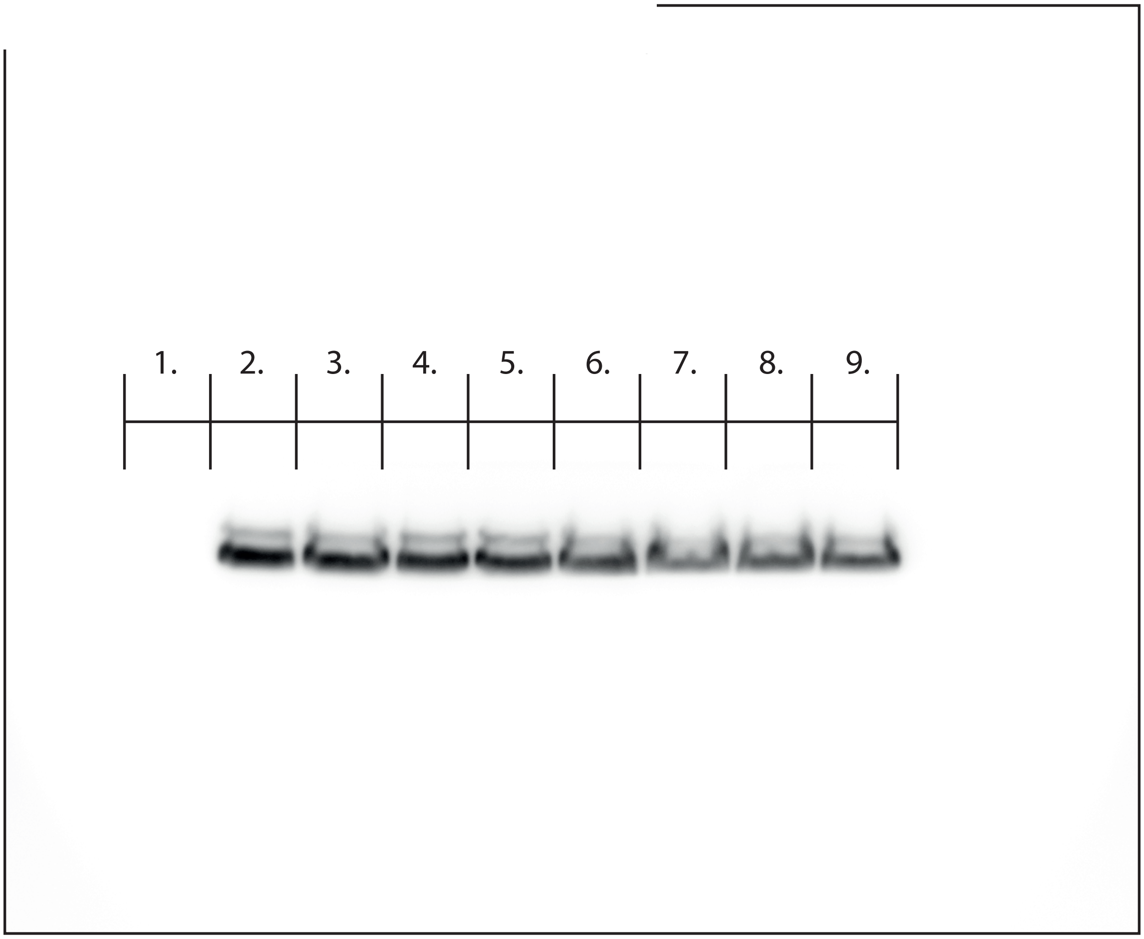

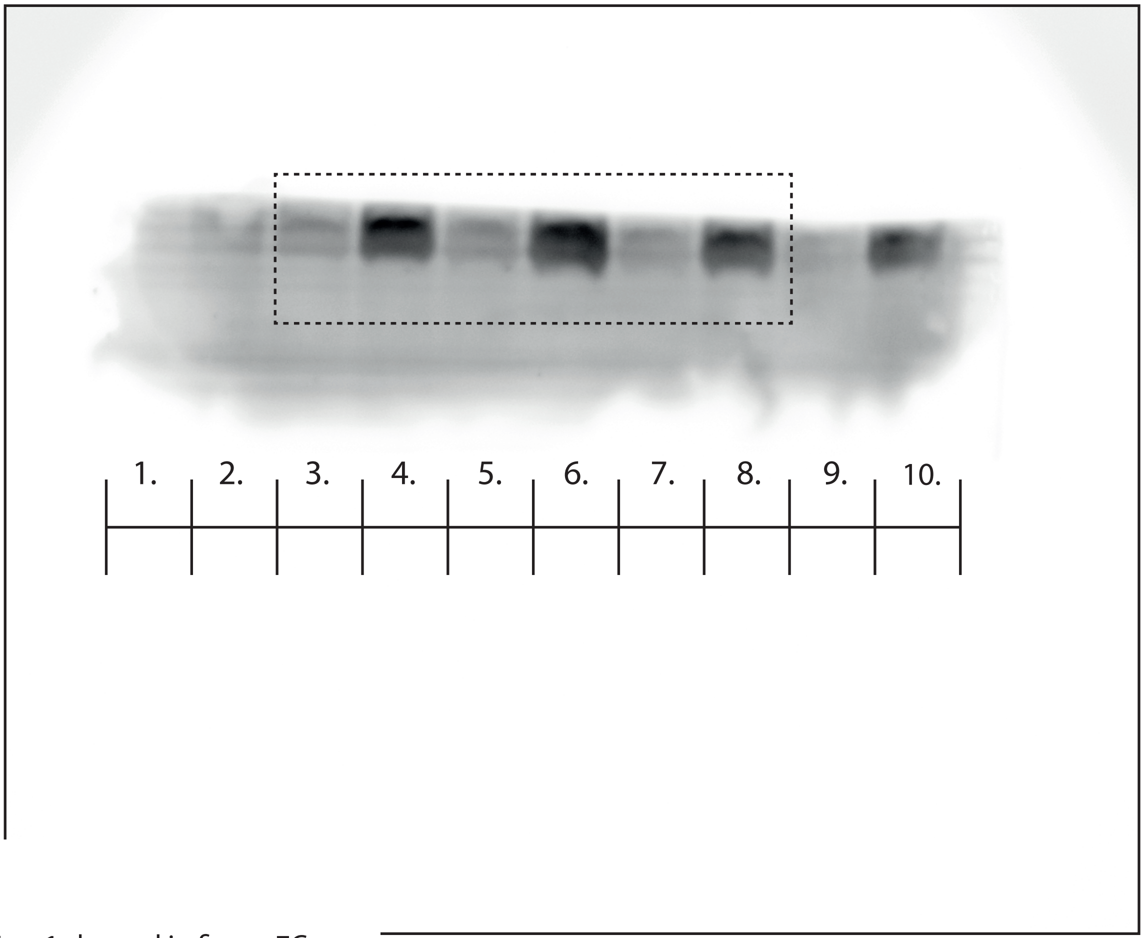


pERK (upper) and total ERK (lower)
i3 neurons Mock or Reelin treated
figure 7C

1. MW St
2.
3. I3neurons day 21, wt mock 10 min-n1 showed in figure 7C
4. I3neurons day 21, wt reelin 10 min-n1 showed in figure 7C
5. I3neurons day 21, wt mock 20 min-n1 showed in figure 7C
6. I3neurons day 21, wt reelin 20 min-n1 showed in figure 7C
7. I3neurons day 21, wt mock 40 min-n1 showed in figure 7C
8. I3neurons day 21, wt reelin 40 min-n1 showed in figure 7C
9.
10.

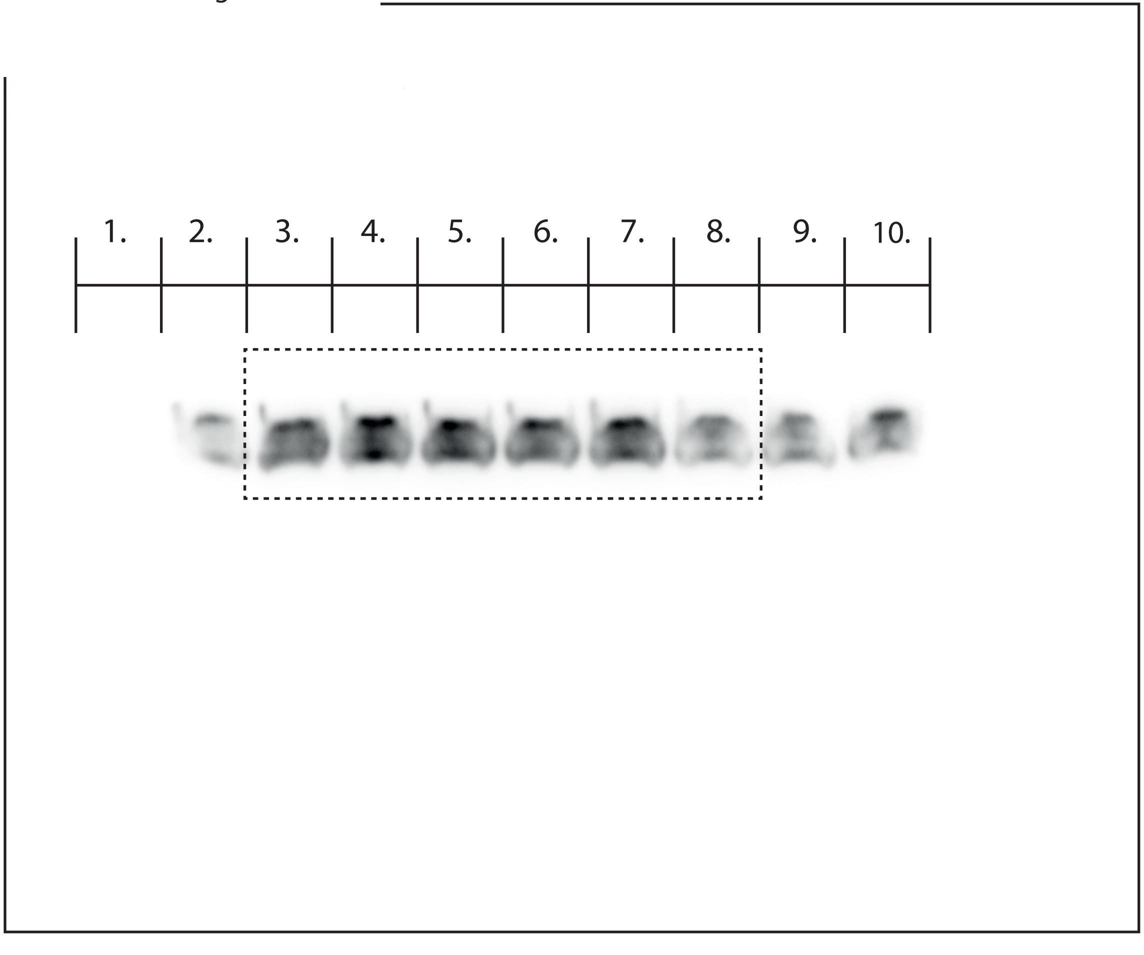

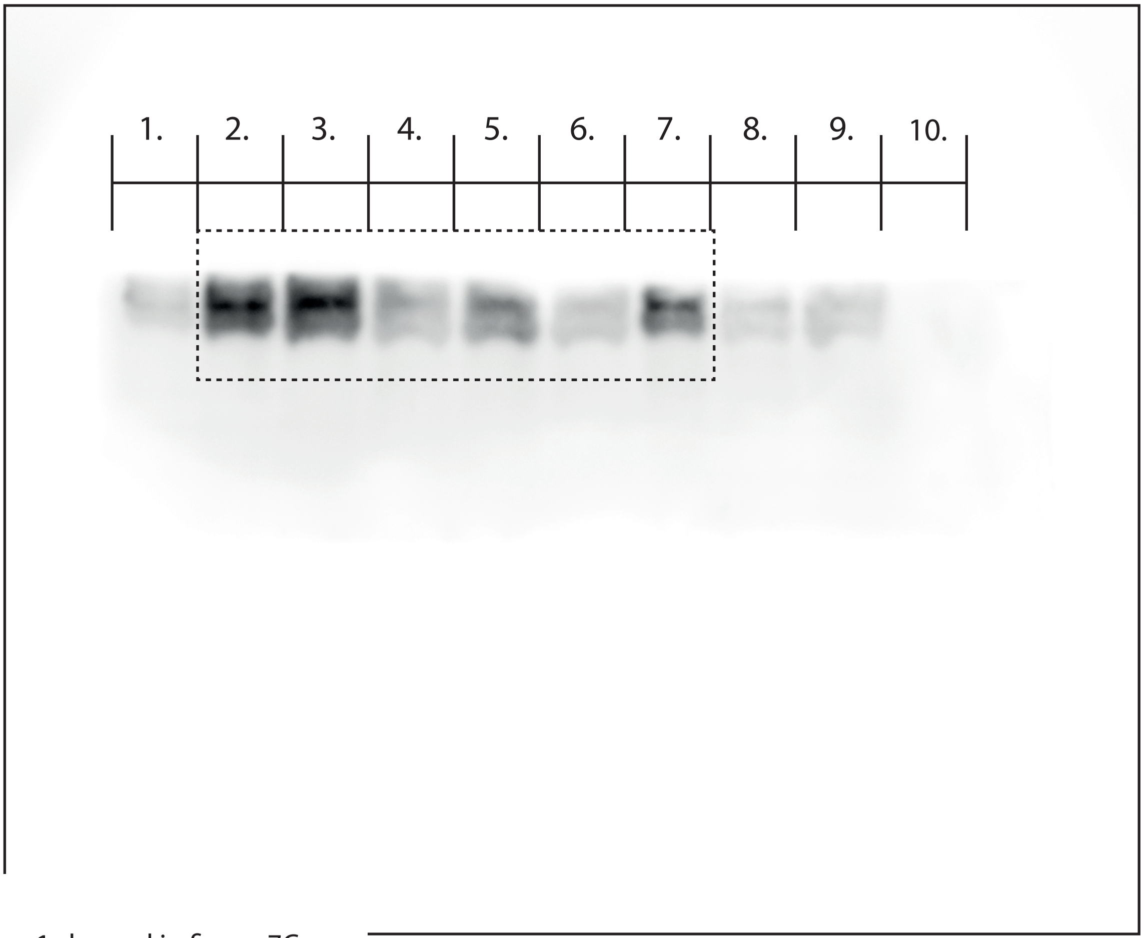


pERK (upper) and total ERK (lower)
i3 neurons Mock or Reelin treated
figure 7C

1. MW St
2. I3neurons day 21, KO mock 10 min-n1 showed in figure 7C
3. I3neurons day 21, KO reelin 10 min-n1 showed in figure 7C
4. I3neurons day 21, KO mock 20 min-n1 showed in figure 7C
5. I3neurons day 21, KO reelin 20 min-n1 showed in figure 7C
6. I3neurons day 21, KO mock 40 min-n1 showed in figure 7C
7. I3neurons day 21, KO reelin 40 min-n1 showed in figure 7C
8.
9.
10.

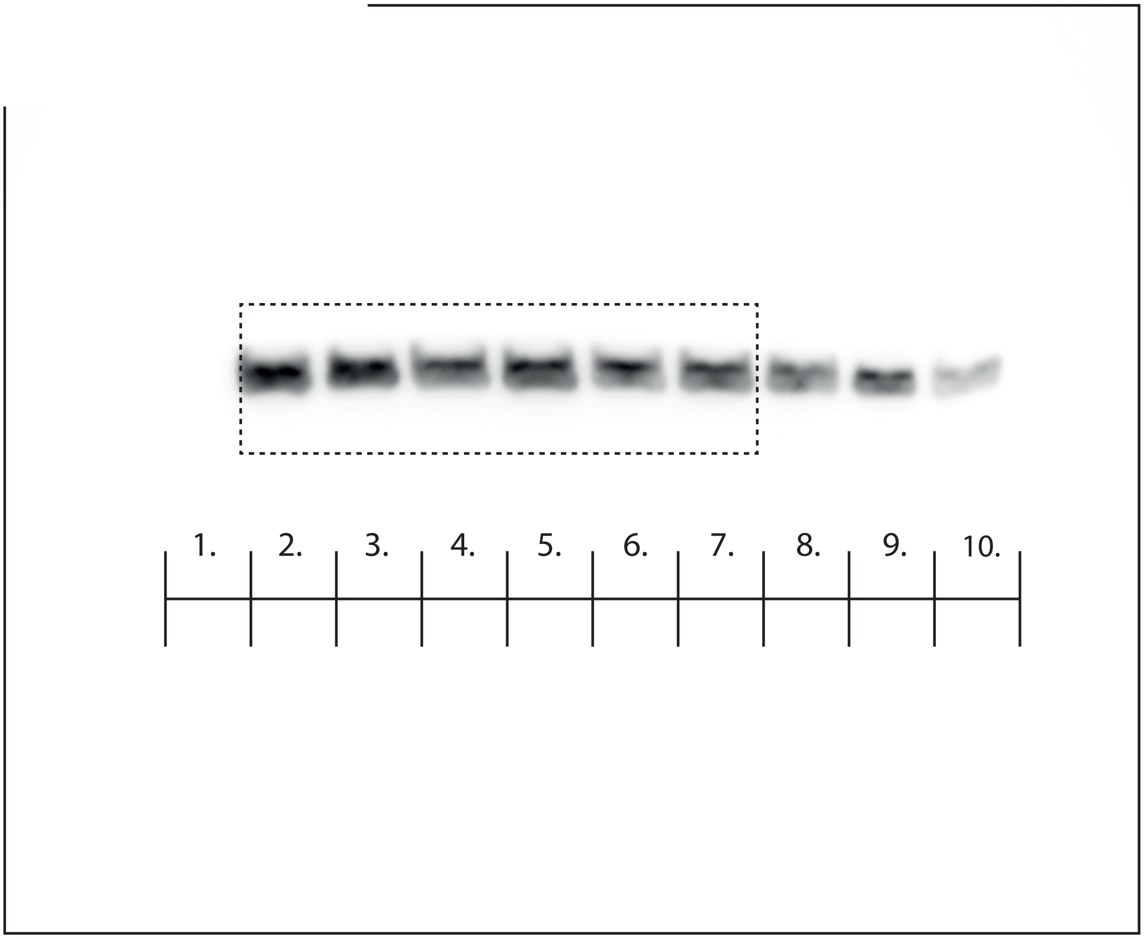

Tubulin
i3 neurons Mock or Reelin treated
figure 7C

1. MW St
2.
3. I3neurons day 21, wt mock 10 min-n1 showed in figure 7C
4. I3neurons day 21, wt reelin 10 min-n1 showed in figure 7C
5. I3neurons day 21, wt mock 20 min-n1 showed in figure 7C
6. I3neurons day 21, wt reelin 20 min-n1 showed in figure 7C
7. I3neurons day 21, wt mock 40 min-n1 showed in figure 7C
8. I3neurons day 21, wt reelin 40 min-n1 showed in figure 7C
9.
10.

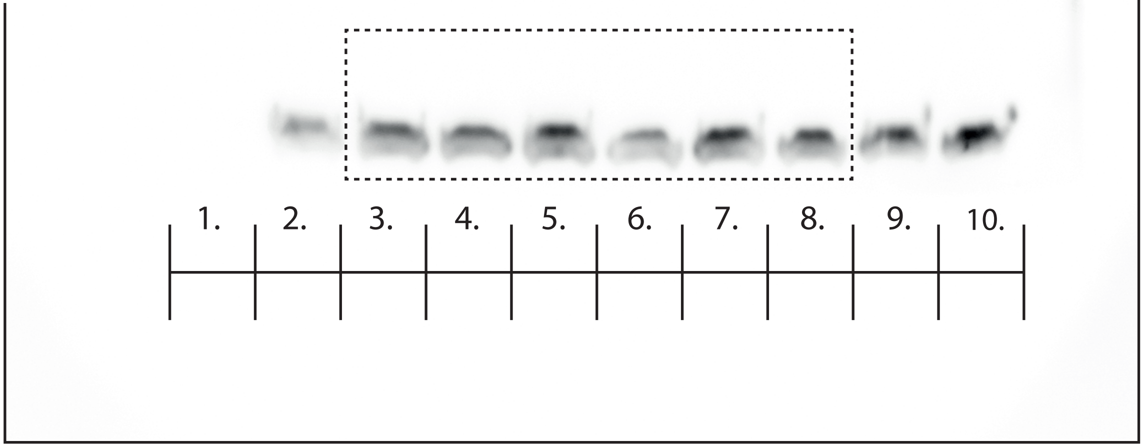


Tubulin
i3 neurons Mock or Reelin treated
figure 7C

1. MW St
2. I3neurons day 21, KO mock 10 min-n1 showed in figure 7C
3. I3neurons day 21, KO reelin 10 min-n1 showed in figure 7C
4. I3neurons day 21, KO mock 20 min-n1 showed in figure 7C
5. I3neurons day 21, KO reelin 20 min-n1 showed in figure 7C
6. I3neurons day 21, KO mock 40 min-n1 showed in figure 7C
7. I3neurons day 21, KO reelin 40 min-n1 showed in figure 7C
8.
9.
10.

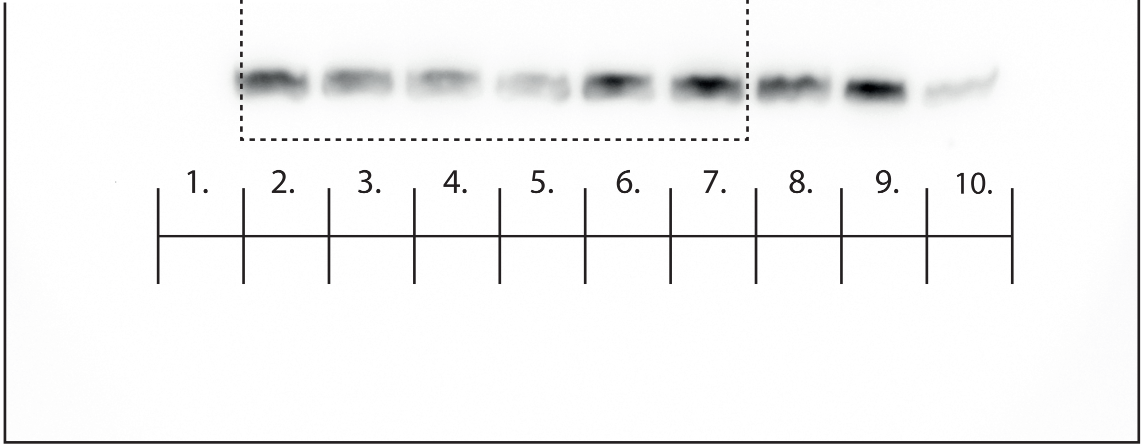

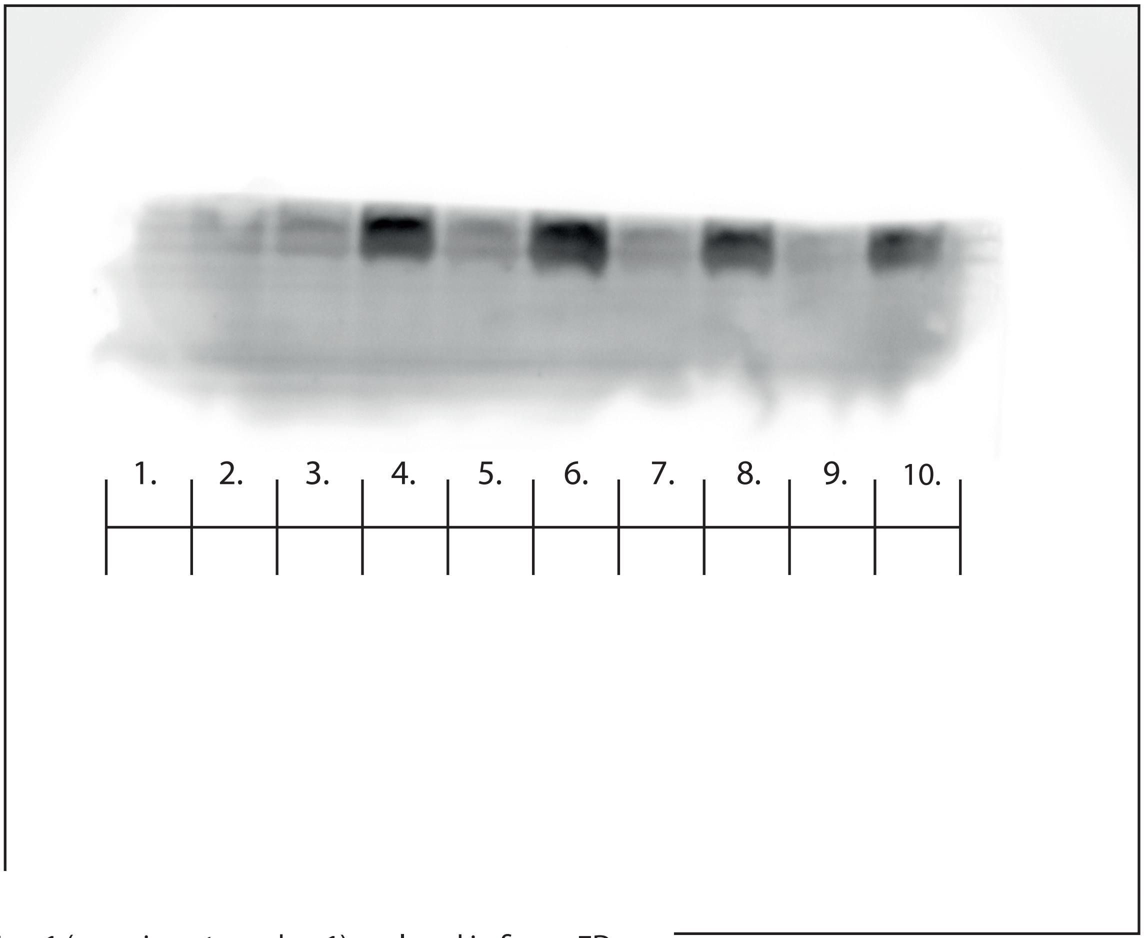


pERK (upper) and total ERK (lower)
i3 neurons Mock or Reelin treated
figure 7D

1. MW St
2.
3. I3neurons day 21, wt mock 10 min-n1 (experiment number 1) analyzed in figure 7D
4. I3neurons day 21, wt reelin 10 min-n1 (experiment number 1) analyzed in figure 7D
5. I3neurons day 21, wt mock 20 min-n1 (experiment number 1) analyzed in figure 7D
6. I3neurons day 21, wt reelin 20 min-n1 (experiment number 1) analyzed in figure 7D
7. I3neurons day 21, wt mock 40 min-n1 (experiment number 1) analyzed in figure 7D
8. I3neurons day 21, wt reelin 40 min-n1 (experiment number 1) analyzed in figure 7D
9.
10.

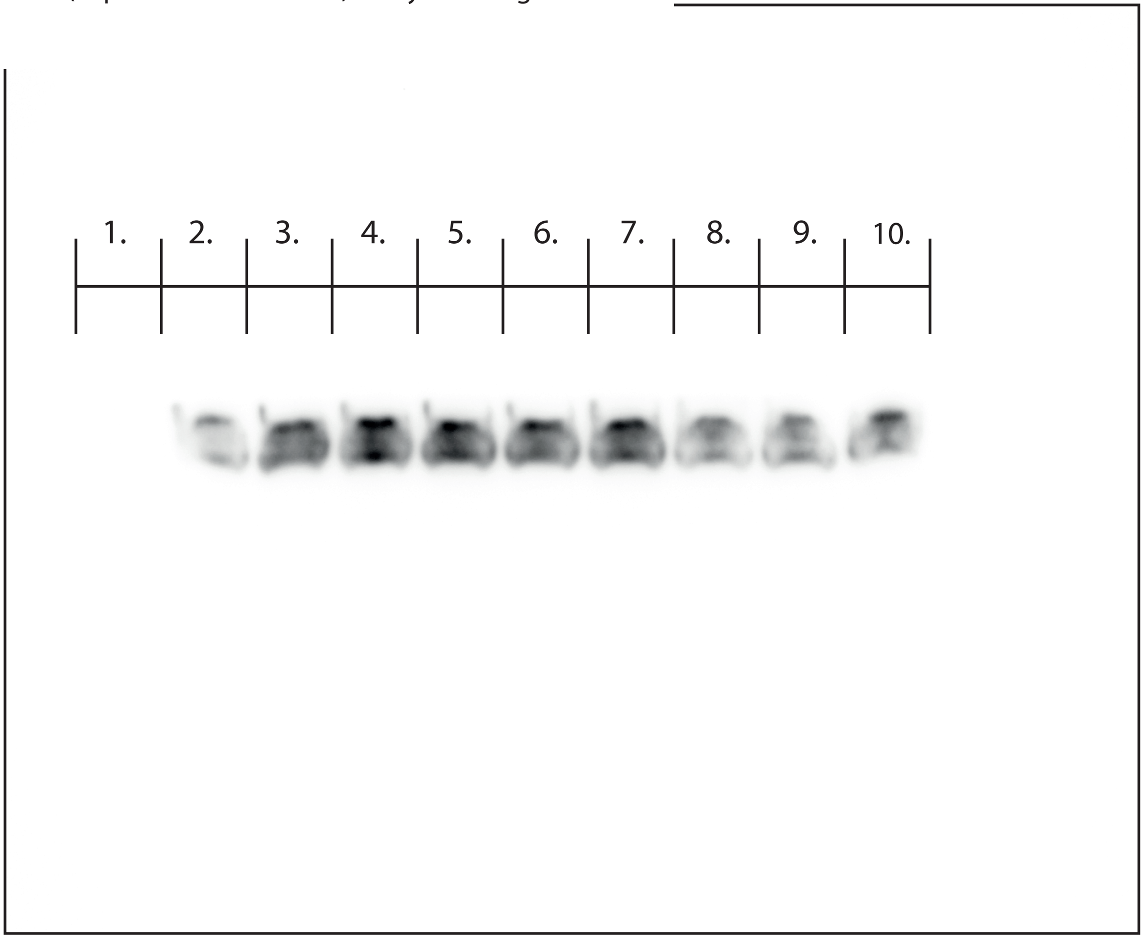

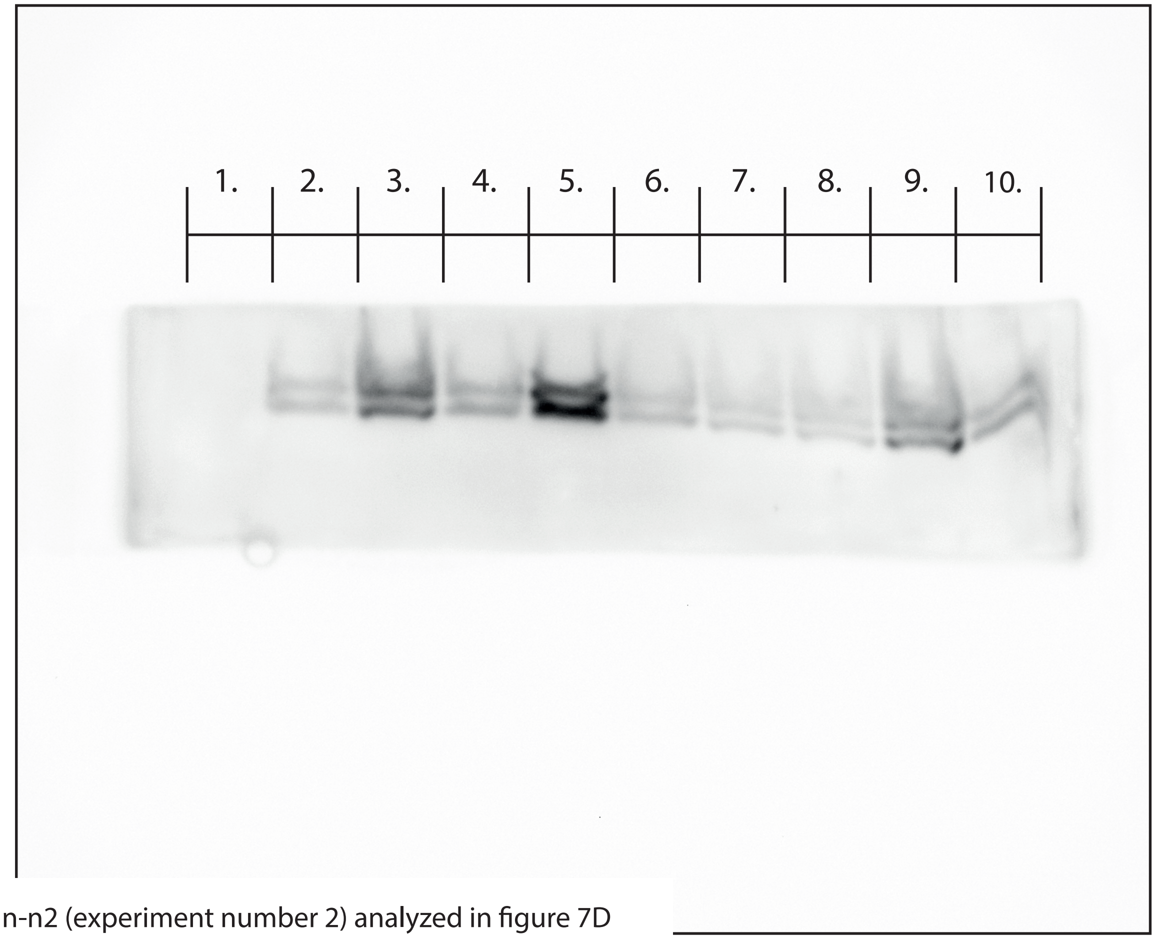


pERK (upper) and total ERK (lower)
i3 neurons Mock or Reelin treated
figure 7D

1. MW St
2. I3neurons day 21, wt mock 10 min-n2 (experiment number 2) analyzed in figure 7D
3. I3neurons day 21, wt reelin 10 min-n2 (experiment number 2) analyzed in figure 7D
4. I3neurons day 21, wt mock 20 min-n2 (experiment number 2) analyzed in figure 7D
5. I3neurons day 21, wt reelin 20 min-n2 (experiment number 2) analyzed in figure 7D
6. I3neurons day 21, wt mock 40 min-n2 (experiment number 2) analyzed in figure 7D
7. I3neurons day 21, wt reelin 40 min-n2 (experiment number 2) analyzed in figure 7D
8.
9.
10.

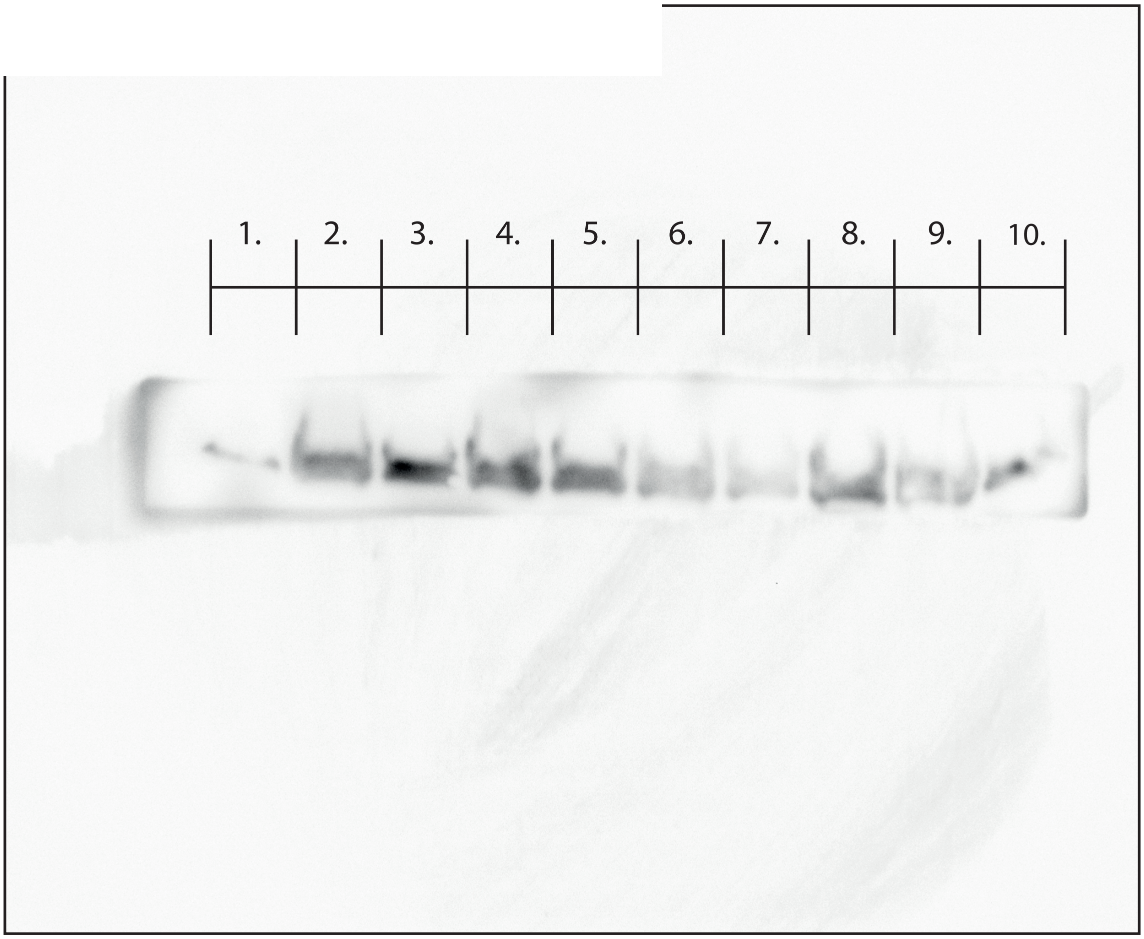

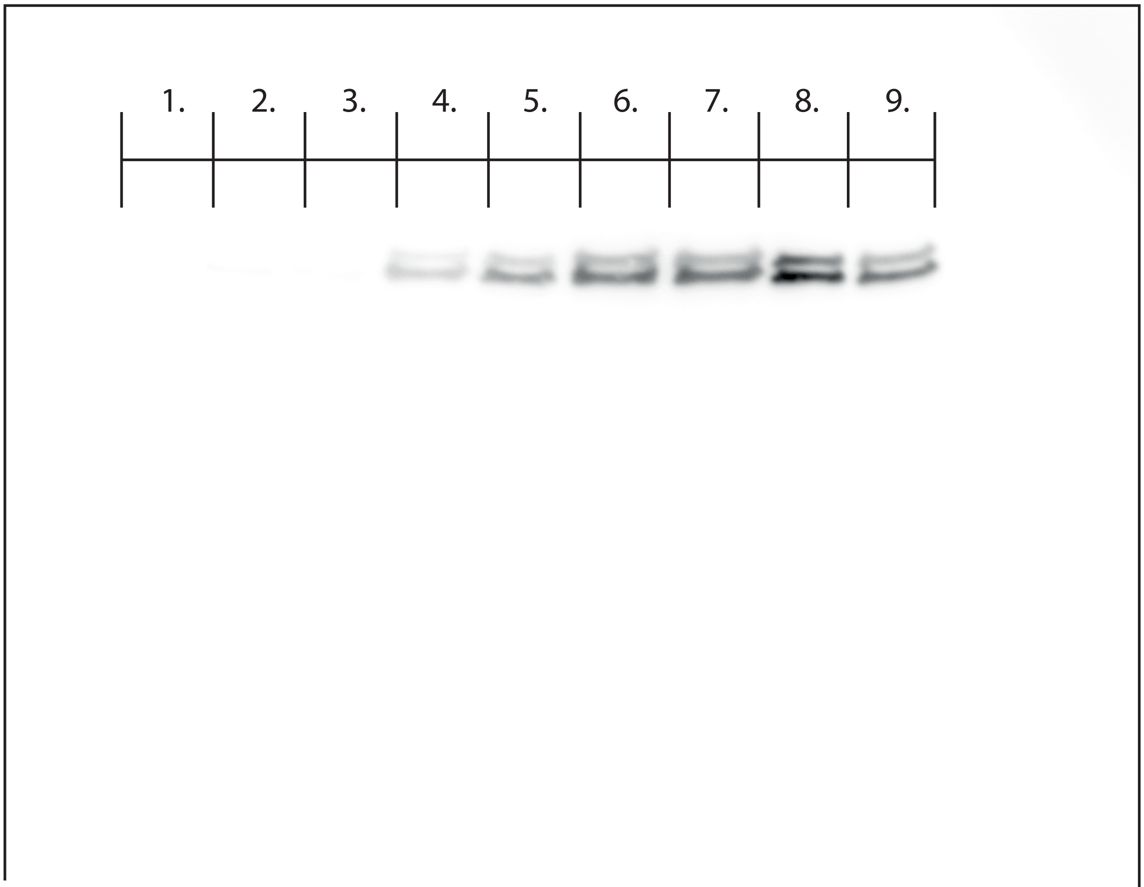


pERK (upper) and total ERK (lower)
i3 neurons Mock or Reelin treated
figure 7D

1. MW St
2. I3neurons day 21, wt mock 10 min-n3 (experiment number 3) analyzed in figure 7D
3. I3neurons day 21, wt reelin 10 min-n3 (experiment number 3) analyzed in figure 7D
4. I3neurons day 21, wt mock 20 min-n3 (experiment number 3) analyzed in figure 7D
5. I3neurons day 21, wt reelin 20 min-n3 (experiment number 3) analyzed in figure 7D
6. I3neurons day 21, wt mock 40 min-n3 (experiment number 3) analyzed in figure 7D
7. I3neurons day 21, wt reelin 40 min-n3 (experiment number 3) analyzed in figure 7D
8.
9.

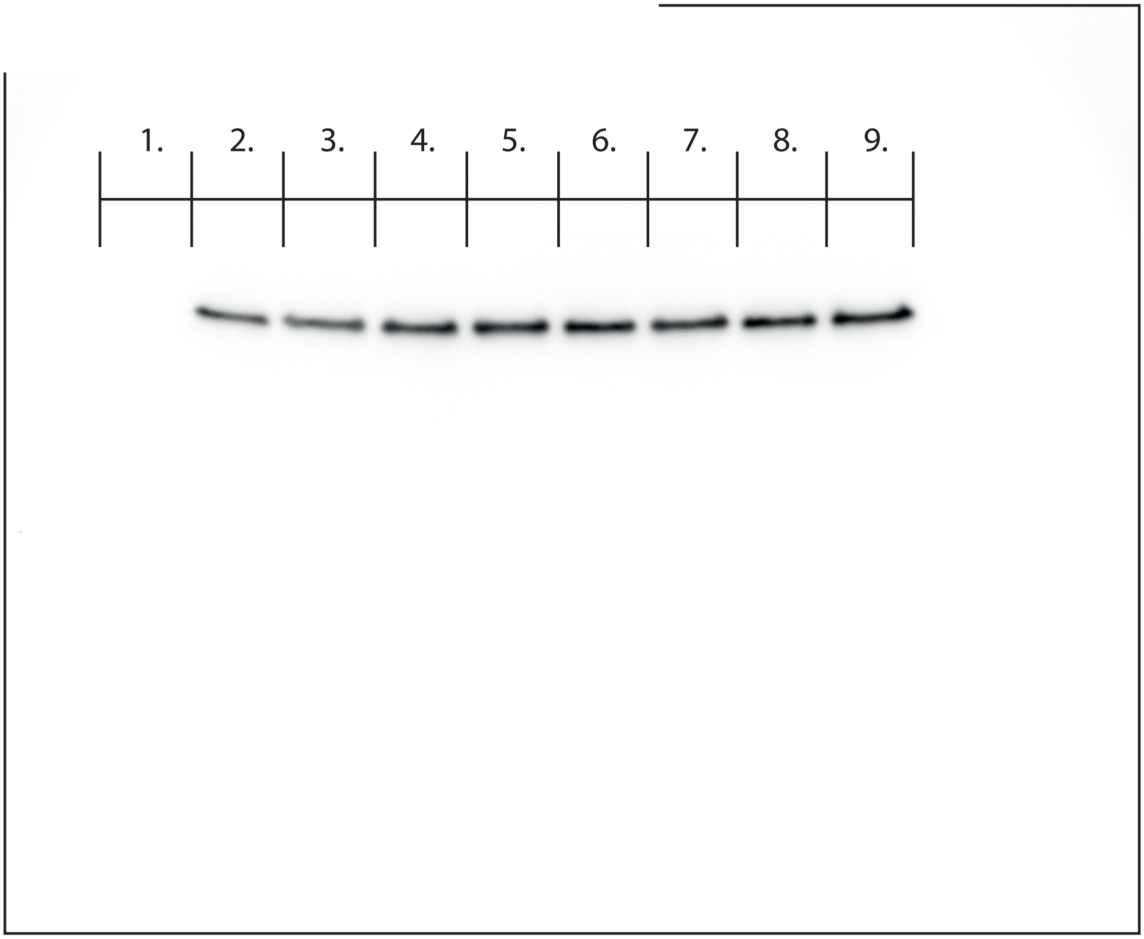

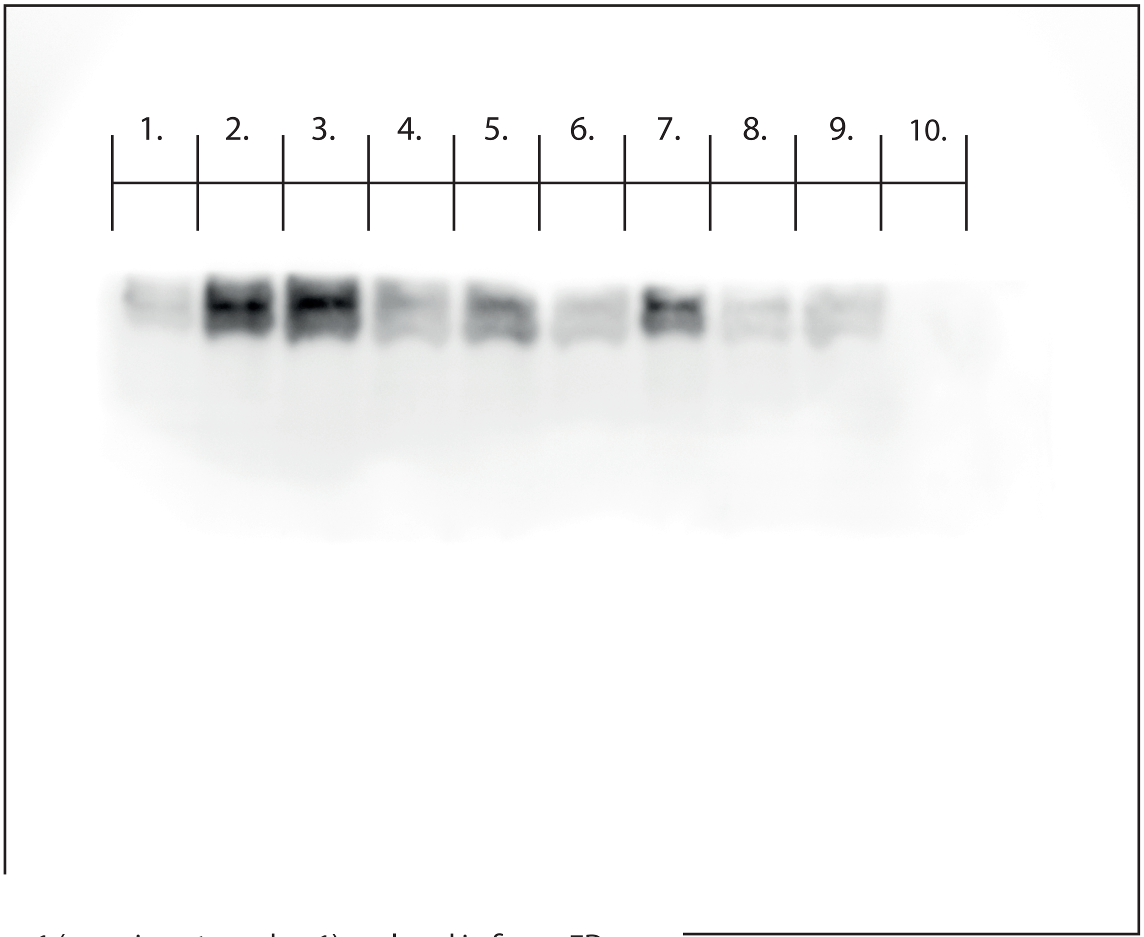


pERK (upper) and total ERK (lower)
i3 neurons Mock or Reelin treated
figure 7D

1. MW St
2. I3neurons day 21, KO mock 10 min-n1 (experiment number 1) analyzed in figure 7D
3. I3neurons day 21, KO reelin 10 min-n1 (experiment number 1) analyzed in figure 7D
4. I3neurons day 21, KO mock 20 min-n1 (experiment number 1) analyzed in figure 7D
5. I3neurons day 21, KO reelin 20 min-n1 (experiment number 1) analyzed in figure 7D
6. I3neurons day 21, KO mock 40 min-n1 (experiment number 1) analyzed in figure 7D
7. I3neurons day 21, KO reelin 40 min-n1 (experiment number 1) analyzed in figure 7D
8.
9.
10.

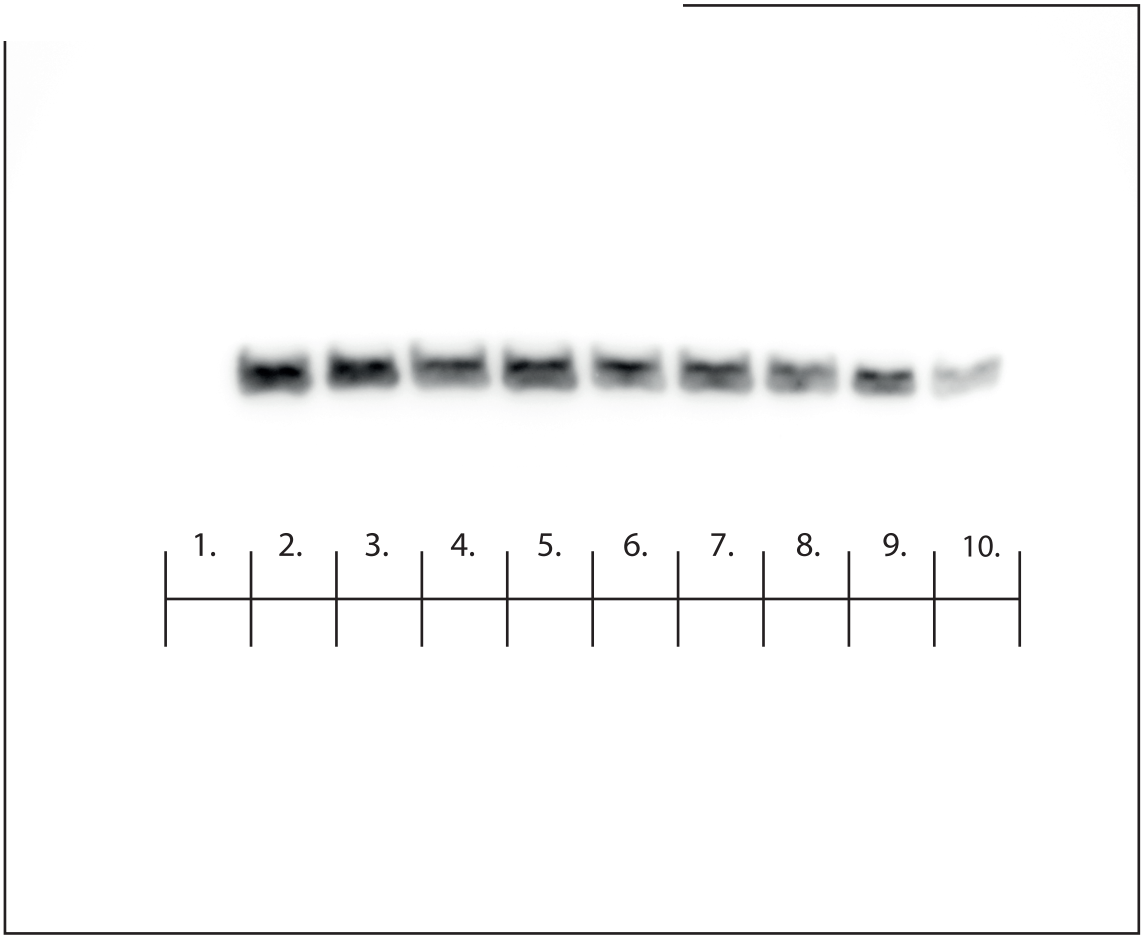

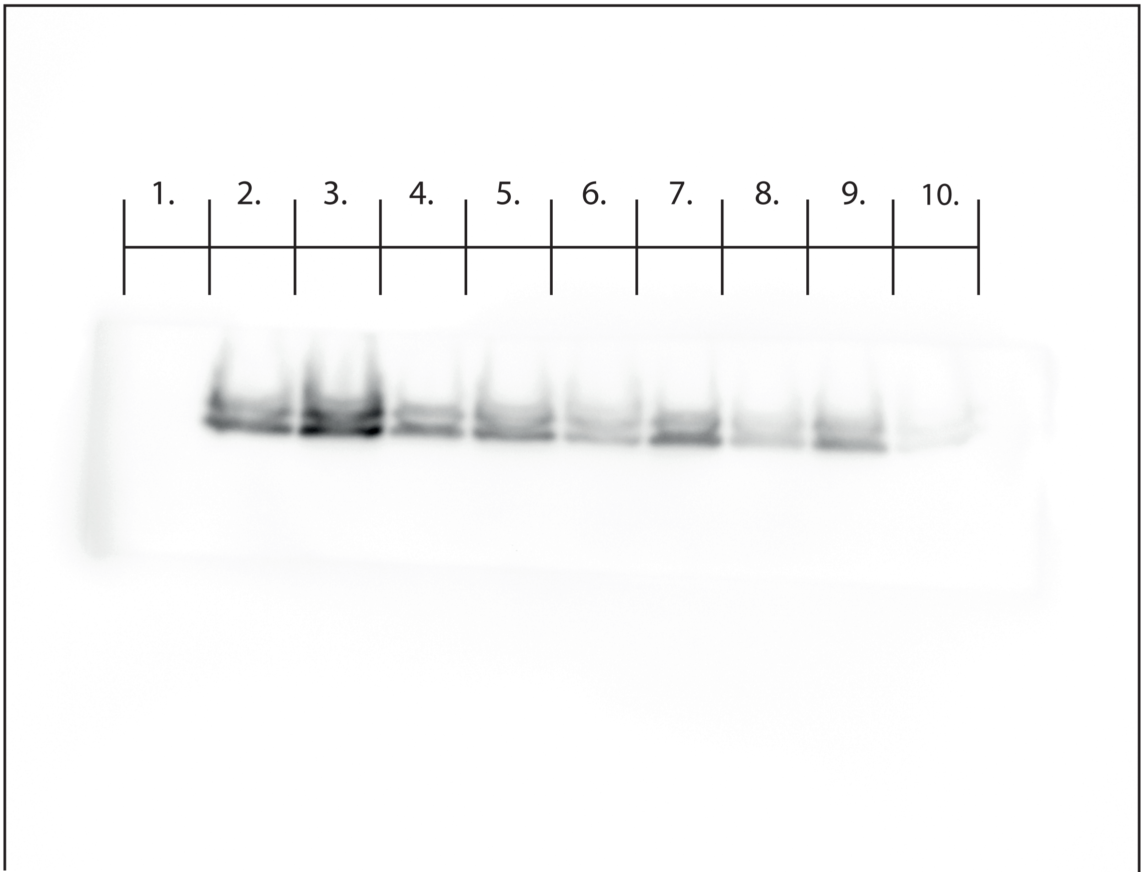


pERK (upper) and total ERK (lower)
i3 neurons Mock or Reelin treated
figure 7D

1. MW St
2. I3neurons day 21, KO mock 10 min-n2 (experiment number 2) analyzed in figure 7D
3. I3neurons day 21, KO reelin 10 min-n2 (experiment number 2) analyzed in figure 7D
4. I3neurons day 21, KO mock 20 min-n2 (experiment number 2) analyzed in figure 7D
5. I3neurons day 21, KO reelin 20 min-n2 (experiment number 2) analyzed in figure 7D
6. I3neurons day 21, KO mock 40 min-n2 (experiment number 2) analyzed in figure 7D
7. I3neurons day 21, KO reelin 40 min-n2 (experiment number 2) analyzed in figure 7D
8.
9.
10.

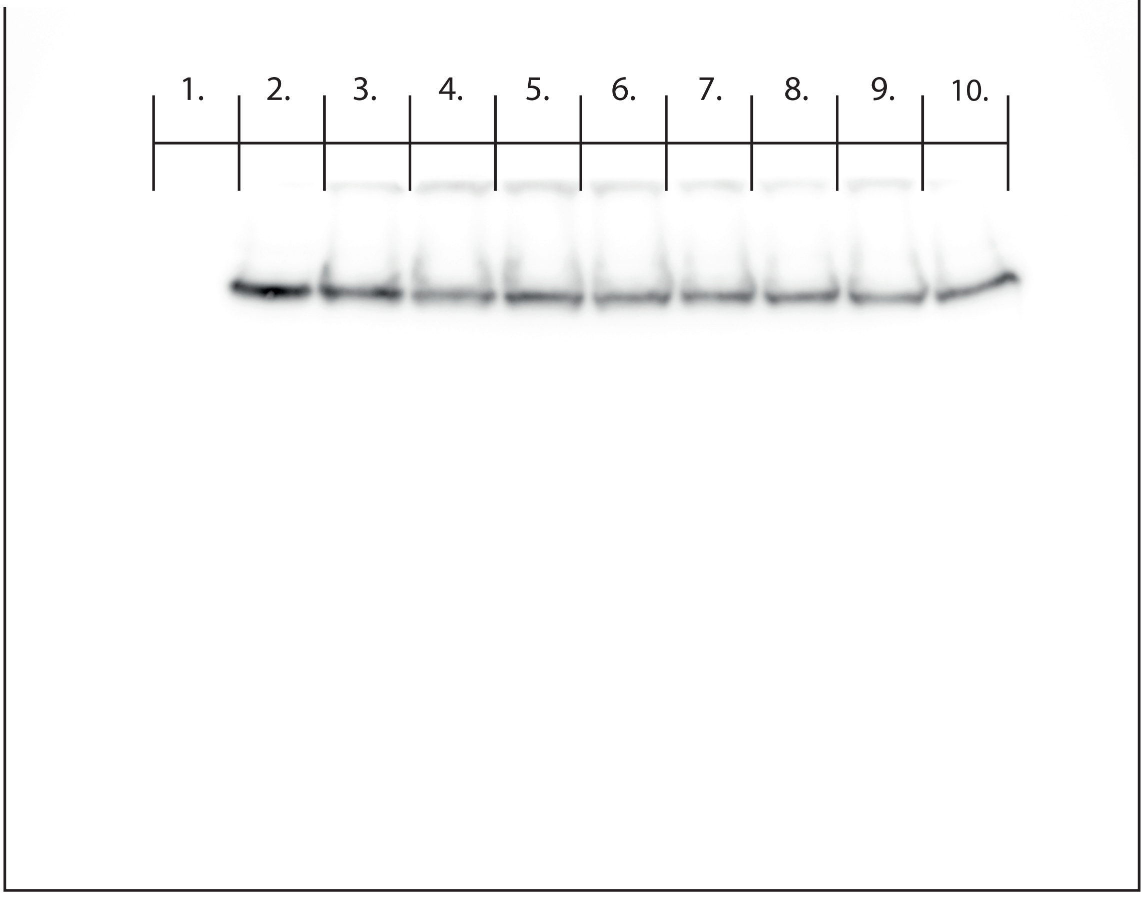

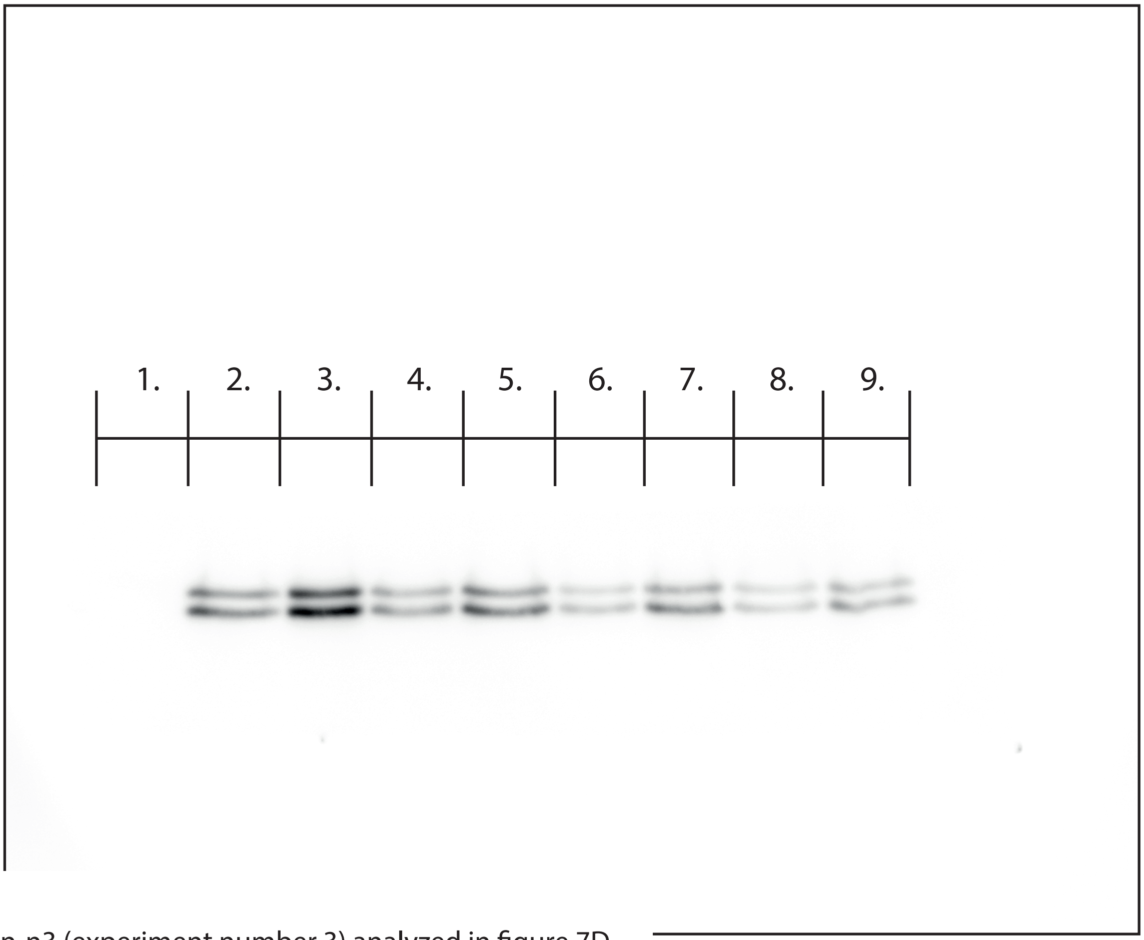


pERK (upper) and total ERK (lower)
i3 neurons Mock or Reelin treated
figure 7D

1. MW St
2. I3neurons day 21, KO mock 10 min-n3 (experiment number 3) analyzed in figure 7D
3. I3neurons day 21, KO reelin 10 min-n3 (experiment number 3) analyzed in figure 7D
4. I3neurons day 21, KO mock 20 min-n3 (experiment number 3) analyzed in figure 7D
5. I3neurons day 21, KO reelin 20 min-n3 (experiment number 3) analyzed in figure 7D
6. I3neurons day 21, KO mock 40 min-n3 (experiment number 3) analyzed in figure 7D
7. I3neurons day 21, KO reelin 40 min-n3 (experiment number 3) analyzed in figure 7D
8.
9.

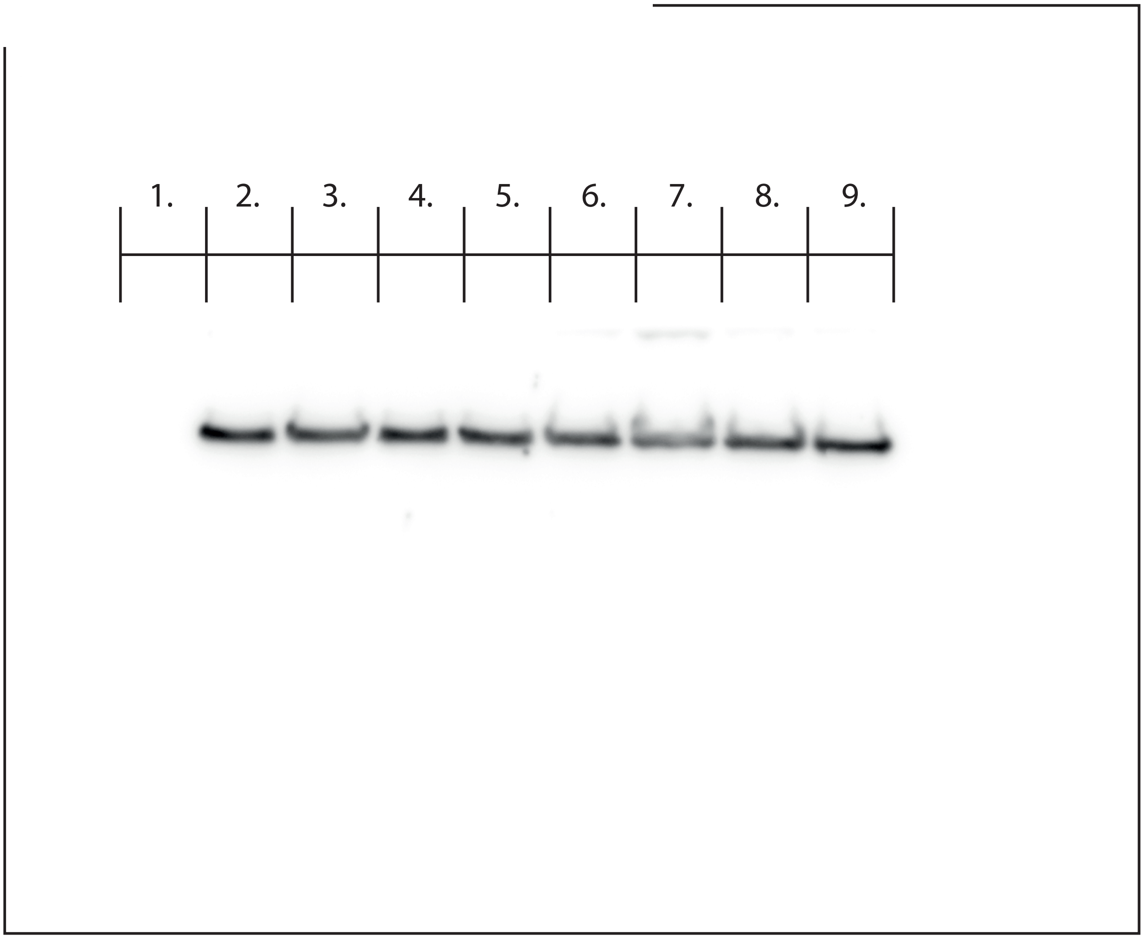

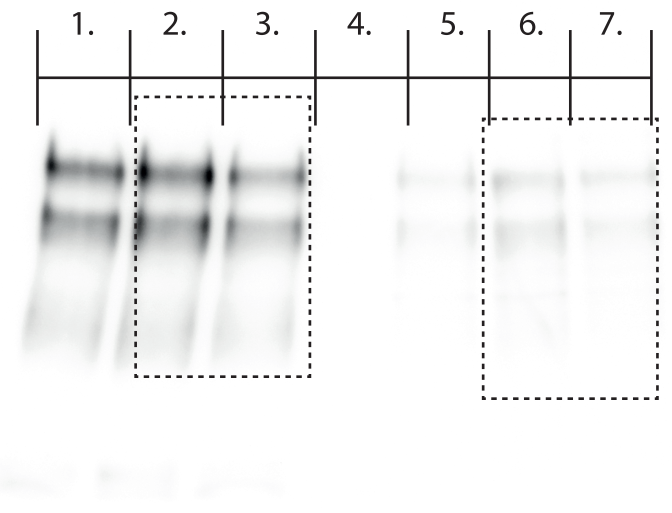


ApoER2
i3 neurons 21 days
figure S2A and B

1.
2. surface i3 neurons wt showed in figure S1E and analyzed in figure S1F (experiment number 1)
3. surface i3 neurons OCRL KO showed in figure S1E and analyzed in figure S1F (experiment number 1)
4. MW St
5.
6. input (2,5%) i3 neurons wt showed (with increased contrast) in figure S1E and analyzed in figure S1F - (experiment number 1)
7. input (2,5%) i3 neurons OCRL KO showed (with increased contrast) in figure S1E and analyzed in figure S1F - (experiment number 1)

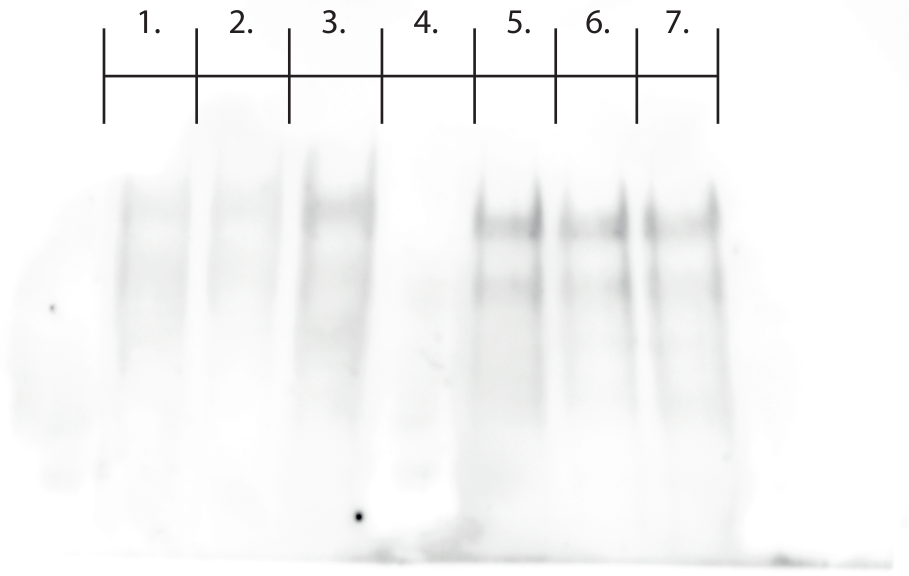


ApoER2
i3 neurons 21 days
figure S2B

1.
2. input (2,5%) i3 neurons OCRL KO analyzed in figure S1F (experiment number 2)
3. input (2,5%) i3 neurons wt analyzed in figure S1F (experiment number 2)
4. MW St
5.
6. surface i3 neurons OCRL KO analyzed in figure S1F - (experiment number 2)
7. surface i3 neurons wt analyzed in figure S1F - (experiment number 2)

New experiments in H4 wt vs KO for figure 5C and D

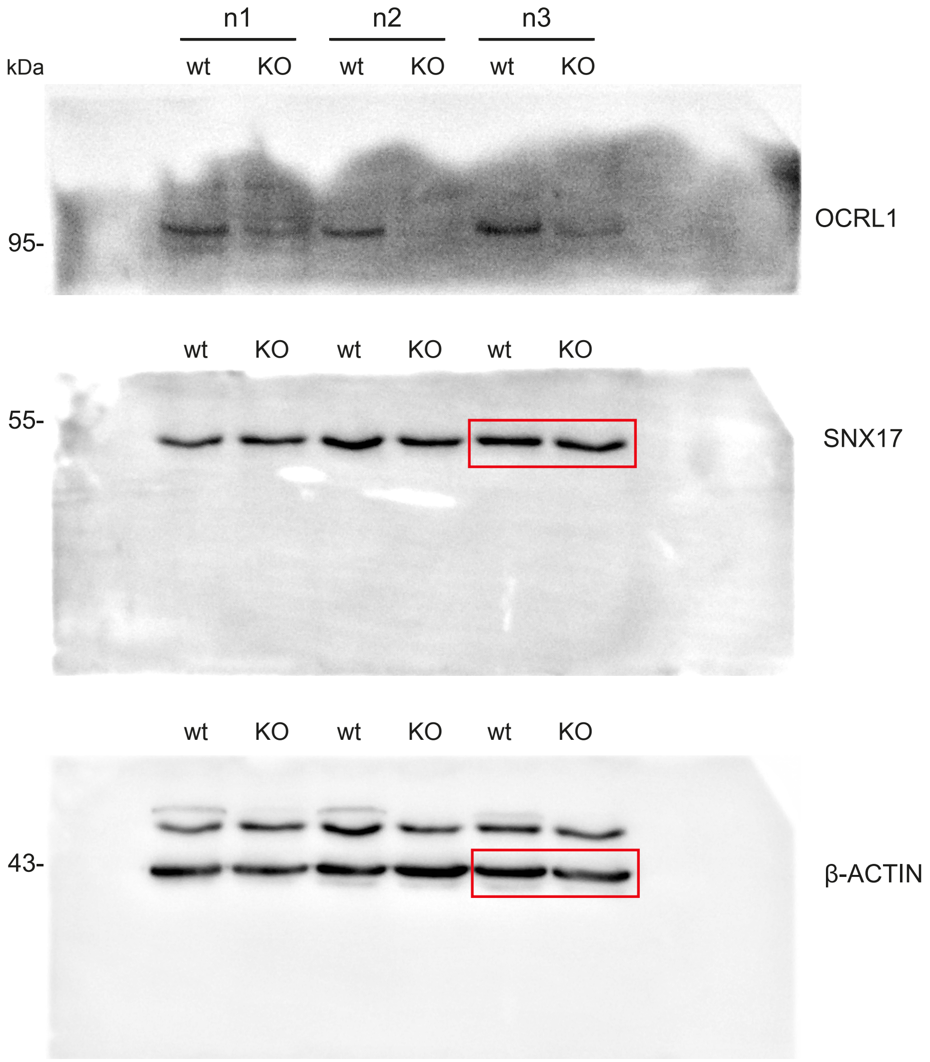

Supplement: Supplementary file 1 [file biomolecules-14-00799-s001.zip › File S1--Western Blotting Figures.pdf]
